# Globally diverse *Mycobacterium tuberculosis* resistance acquisition: a retrospective geographical and temporal analysis of whole genome sequences

**DOI:** 10.1016/s2666-5247(20)30195-6

**Published:** 2021-01-27

**Authors:** Yasha Ektefaie, Avika Dixit, Luca Freschi, Maha R Farhat

**Affiliations:** Department of BioEngineering, University of California Berkeley, Berkeley, CA, USA; Department of Biomedical Informatics, Harvard Medical School, Boston, MA, USA; Department of Biomedical Informatics, Harvard Medical School, Boston, MA, USA; Division of Infectious Disease, Department of Pediatrics, Boston Children’s Hospital, Boston, MA, USA; Department of Biomedical Informatics, Harvard Medical School, Boston, MA, USA; Department of Biomedical Informatics, Harvard Medical School, Boston, MA, USA; Division of Pulmonary and Critical Care, Department of Medicine, Massachusetts General Hospital, Boston, MA, USA

## Abstract

**Background:**

*Mycobacterium tuberculosis* whole genome sequencing (WGS) data can provide insights into temporal and geographical trends in resistance acquisition and inform public health interventions. We aimed to use a large clinical collection of *M tuberculosis* WGS and resistance phenotype data to study how, when, and where resistance was acquired on a global scale.

**Methods:**

We did a retrospective analysis of WGS data. We curated a set of clinical *M tuberculosis* isolates with high-quality sequencing and culture-based drug susceptibility data (spanning four lineages and 52 countries in Africa, Asia, the Americas, and Europe) using public databases and literature curation. For inclusion, sequence quality criteria and country of origin data were required. We constructed geographical and lineage specific *M tuberculosis* phylogenies and used Bayesian molecular dating with BEAST, version 1.10.4, to infer the most recent common susceptible ancestor age for 4869 instances of resistance to ten drugs.

**Findings:**

Between Jan 1, 1987, and Sept 12, 2014, of 10 299 *M tuberculosis* clinical isolates, 8550 were curated, of which 6099 (71%) from 15 countries met criteria for molecular dating. The number of independent resistance acquisition events was lower than the number of resistant isolates across all countries, suggesting ongoing transmission of drug resistance. Ancestral age distributions supported the presence of old resistance, 20 years or more before, in most countries. A consistent order of resistance acquisition was observed globally starting with resistance to isoniazid, but resistance ancestral age varied by country. We found a direct correlation between gross domestic product per capita and resistance age (*r*^2^=0·47; p=0·014). Amplification of fluoroquinolone and second-line injectable resistance among multidrug-resistant isolates is estimated to have occurred very recently (median ancestral age 4·7 years [IQR 1·9–9·8] before sample collection). We found the sensitivity of commercial molecular diagnostics for second-line resistance to vary significantly by country (p<0·0003).

**Interpretation:**

Our results highlight that both resistance transmission and amplification are contributing to disease burden globally but vary by country. The observation that wealthier nations are more likely to have old resistance (most recent common susceptible ancestor >20 years before isolation) suggests that programmatic improvements can reduce resistance amplification, but that fit resistant strains can circulate for decades subsequently implies the need for continued surveillance.

## Introduction

The global epidemic of tuberculosis is responsible for more deaths than any other infection due to a single pathogen.^1^ The emergence of multidrug-resistant and extensively drug-resistant tuberculosis presents a major obstacle to efforts to accelerate tuberculosis decline. Halting the transmission of drug-resistant tuberculosis has been a major focus of studies addressing the emergence of drug-resistant tuberculosis.^2^ But the epidemic is ultimately defined by local factors that remain understudied in many parts of the world.^3^ The study of geographical and temporal heterogeneity of the drug-resistant tuberculosis epidemic can provide insights into these local factors as key drivers of multidrug-resistant tuberculosis prevalence and persistence in the community, including programmatic and bacterial factors. This understanding is key to future disease control and prevention of antibiotic resistance development.

Over the past decade, increased uptake of molecular and whole genome sequencing (WGS) technologies, and their application to *Mycobacterium tuberculosis* clinical isolates, has offered novel insights into pathogen biology and diversity in the context of human infection.^4–7^ The application of WGS has allowed us to better understand the genetic determinants of drug resistance within *M tuberculosis*.^8^ The detection of these genetic determinants using molecular technologies that include WGS is now increasingly adopted for tuberculosis resistance diagnosis in many parts of the world^9^ and is beginning to replace the more biohazardous and time consuming culture-based drug susceptibility tests. The study of isolates sampled from epidemiological outbreaks or from the same host over time has allowed the estimation of *M tuberculosis*’ molecular clock rate, or temporal rate of accumulation of fixed genome-level variation.^9,10^ The application of this rate to new WGS data from isolates collected for surveillance has helped improve transmission inference and molecular dating of specific evolutionary events such as resistance acquisition or lineage divergence.^10–12^

We aimed to use a large clinical collection of *M tuberculosis* WGS and resistance phenotype data to study how, when, and where resistance was acquired on a global scale. We also assessed the distribution of unexplained *M tuberculosis* phenotypic resistance across 20 countries, to evaluate the accuracy and geographical heterogeneity of molecular detection of common *M tuberculosis* genetic resistance determinants, and discuss implications for drug-resistant tuberculosis control.

## Methods

### Study design

We did a retrospective analysis of WGS data, in which we curated a set of clinical *M tuberculosis* isolates with high-quality sequencing and culture-based drug susceptibility data. Using a Bayesian implementation of coalescent theory, we estimated and compared dates of resistance acquisition for multidrug-resistant and extensively drug-resistant isolates across 15 different countries and unexplained phenotypic resistance across 23 countries (appendix p 5). We used the recency of resistance acquisition as a measure of fitness of the circulating strains in their respective environments and studied the effect of gross domestic product per capita, as a proxy for tuberculosis control programme funding, on the recency of resistance acquisition at a macro level. Further details are available in the appendix (p 1). We compiled a 10 299 *M tuberculosis* WGS dataset with culture-based drug susceptibility test (phenotypic) data using public data bases (Patric^13^ and ReSeqTB^14^) and literature curation.^9,11,12,15^ A summary table with the phenotypic data is available online.

Drugs assessed were isoniazid, ethambutol, rifamycins (rifampicin or rifabutin), streptomycin, pyrazinamide, fluoroquinolones (includes moxifloxacin, ciprofloxacin, ofloxacin), second-line injectables (includes kanamycin, amikacin, capreomycin), ethionamide–prothionamide, and cycloserine. Para-aminosalicylic acid was not analysed due to the paucity of data. Isolates not tested for susceptibility to both isoniazid and rifamycins were excluded from the assessment of drug resistance frequency by country and lineage. Isolates resistant to both isoniazid and rifamycins were labelled multidrug-resistant. Those resistant to isoniazid, rifamycins, fluoroquinolones, and second-line injectables were labelled extensively drug-resistant.

### Procedures

Isolates were separated into 179 groups corresponding to a single drug, lineage, and source country, referred to hereafter as a group. Genetic diversity was computed as the mean pairwise genetic distance within a group. To accurately date resistance acquisition, a drug-geography-lineage group was analysed only if it consisted of at least ten isolates, 20% or more of isolates were susceptible, and one or more isolates were resistant. To exclude isolates that only represented outbreak settings and did not carry more long-term information about resistance, we excluded groups with a genetic diversity score more than one SD less than the mean genetic diversity measured across all groups. Sensitivity analyses on these thresholds, the phylogeny construction, and the estimation of the age of the most recent susceptible common ancestor (MRSCA) in the years before isolation of the clinical sample are detailed in the appendix (p 2).

We compared the expected sensitivity and specificity of mutations captured by commercial diagnostics (Hain Line-probe, Hain LifeScience, Nehren, Germany and GeneXpert, Cepheid, Sunnyvale, USA; summarised from the literature in the appendix p 6) and those based on more extensive lists of mutations in drug-resistant genes, which can be captured using targeted sequencing or WGS. We used three mutations lists for WGS: (1) a set of 267 common resistance-associated mutations that we previously determined using randomForests^16^ designated RF-select WGS test; (2) a set of mutations determined using direct association^8^ designated DA-select WGS test; and (3) any non-synonymous mutation or non-coding mutation in known drug-resistant regions (appendix p 7) in an all WGS test. This all WGS test was included to identify a ceiling on sensitivity and as a point of comparison for the select WGS test. We excluded previously described neutral or lineage-associated mutations.^9^

### Role of the funding source

The funders of the study had no role in study design, data collection, data analysis, data interpretation, or writing of the report. All authors had full access to all of the data in the study and the final responsibility to submit for publication.

## Results

**Figure 1:**
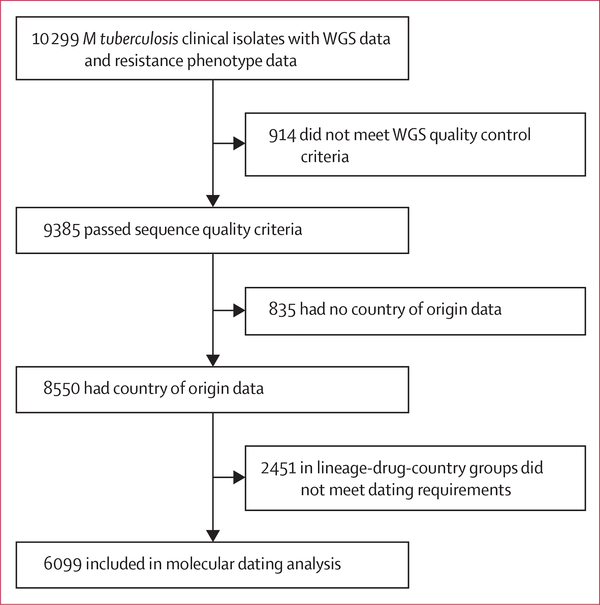
Study profile The process of identification and exclusion of genomic data included in the study is shown. *M tuberculosis=Mycobacterium tuberculosis*. WGS=whole genome sequencing.

**Figure 2:**
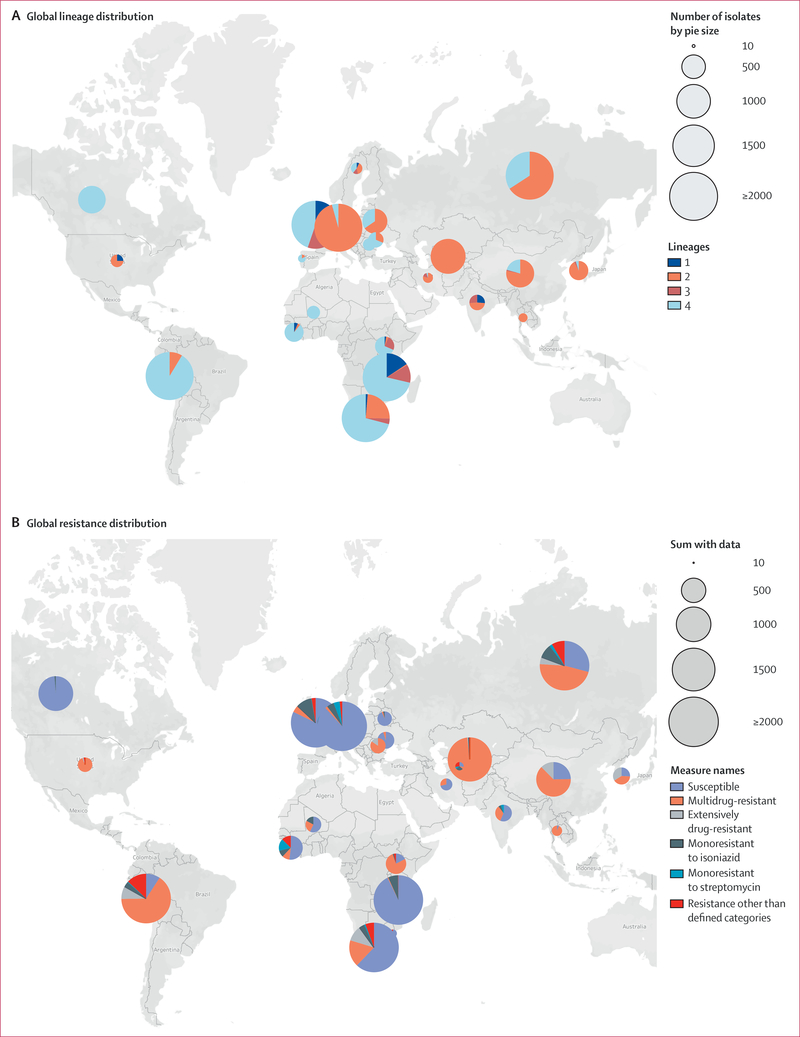
Global distribution of *Mycobacterium tuberculosis* in the study sample For global lineage distribution (n=8477), pie charts represent the proportion of each lineage among isolates available from each country. For global resistance distribution (n=7834), pie charts show the distribution of resistance patterns by country. Pie size is proportional to the number of isolates from each country (appendix pp 8–9). Counts from countries represented by fewer than ten isolates (n=75) not shown.

Between Jan 1, 1987, and Sept 12, 2014, of the 10 299 *M tuberculosis* clinical isolates with WGS and culture-based drug susceptibility tests data available, 9385 (91%) passed sequence quality criteria, and of these, 8550 had country of origin data (figure 1). The four major *M tuberculosis* lineages, 1–4, were well represented (figure 2A, appendix p 8). The non-Europe, America, and Africa lineages (lineages 1–3) comprised 1580 (40%) of 3956 European isolates and 86 (7%) of 1297 North and South American isolates.

Of the 8550 isolates, 641 (7%) did not have isoniazid or rifamycin drug susceptibility test data or originated from countries represented by fewer than ten isolates. Of the remaining 7909 isolates, 5022 (63%) were pan-susceptible (ie, susceptible to all drugs tested), 2887 (37%) were resistant to one or more drugs (drug-resistant), and of these, 1937 (67%) were resistant to isoniazid and rifamycins (multidrug-resistant) and 288 (10%) were multidrug-resistant and resistant to a second-line injectable and a fluoroquinolone (extensively drug-resistant). The 8550 isolates originated from 52 countries. Of these, 23 countries were represented by more than ten isolates with resistance data, 21 of 23 were found to have multidrug-resistant isolates, and nine of 21 had extensive drug-resistance (figure 2B, appendix p 9). The multidrug-resistant tuberculosis proportion in our sample overlapped with that of the WHO findings in four (19%) countries,^17^ was higher in 14 (67%) countries, and lower in three (14%) countries (appendix p 13). Multidrug resistance rates by lineage were 3% (n=439) for lineage 1, 48% (n=1085) for lineage 2, 4% (n=760) for lineage 3, and 23% (n=3358) for lineage 4.

Of the 8550 isolates, 2451 (29%) appeared in groups that did not meet our dating requirements. The remaining 6009 included 1547 isolates resistant to one or more drugs and were grouped into 179 country, lineage, and drug combinations (appendix p 14). We estimated 4860 MRSCA dates for ten drugs across these 179 groups. The number of independent resistance acquisition events (ie, unique MRSCA dates) was consistently lower than the total number of dated resistant isolates, suggesting ongoing transmission of drug-resistant isolates (appendix p 20). We estimated a lower bound on the burden of resistance due to transmission ranging by country from 14% or more to 52% or more pooled across drugs (appendix p 20). The proportion of resistance attributed to transmission was highest among the ten drugs at 43% or more for isoniazid and 46% or more for rifamycin pooled across countries (appendix p 20).

**Figure 3:**
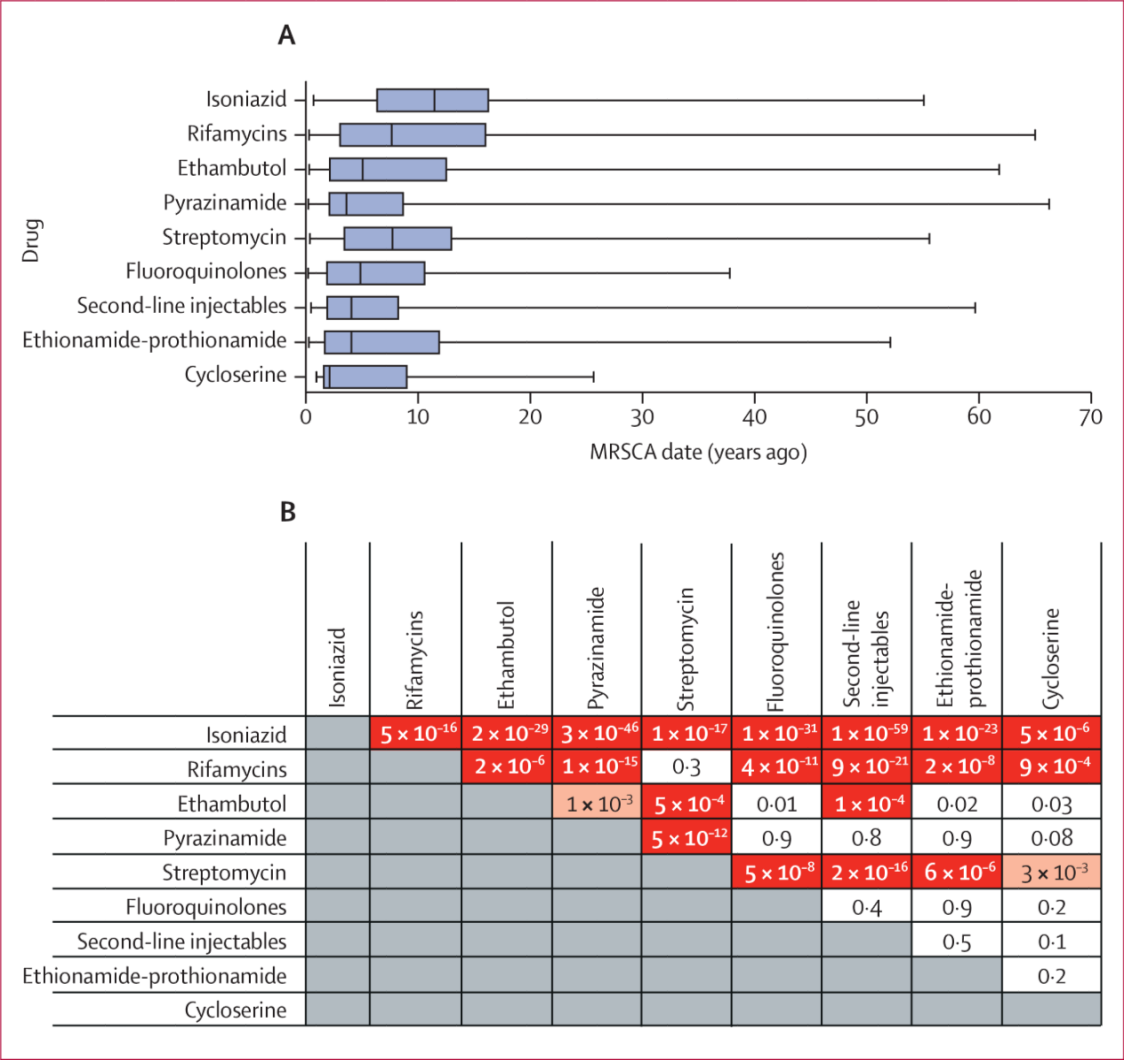
MRSCA distribution by drug (n=4844) and pairwise Wilcoxon rank sum tests comparing MRSCA ages by drug category Boxplots showing range of MRSCA distribution globally for nine tuberculosis drugs (A) and pairwise Wilcoxon rank sum tests comparing MRSCA ages by drug category (B). Red indicates p<0·001 (Bonferroni threshold); pink indicates p<0·01; and white indicates p≥0·01. MRSCA=most recent susceptible common ancestor.

We examined the relative order of phenotypic resistance acquisition pooled across all countries and lineages. We found that resistance to isoniazid developed before resistance to other drugs (figure 3A, B). Median MRSCA for isoniazid was 11·4 years (IQR 6·3–16·2) before isolation versus 7·6 years (3·0–16·0) for rifamycins (Wilcoxon rank-sum test p<0·0001). Median MRSCA ages for rifamycin resistance (7·6 years [IQR 3·0–16·0]) and streptomycin resistance (7·7 years [3·4–13·0]) were second oldest and not significantly different from each other (Wilcoxon rank-sum test p=0·31). We examined if there was a second older date peak in the streptomycin MRSCA histogram (appendix p 67)—around when streptomycin was used as monotherapy in the 1940s to the 1950s—and found that only 0·6% of streptomycin MRSCA dates were 50 years or more before. The dating supported that ethambutol resistance followed the acquisition of rifamycin resistance (Wilcoxon rank-sum test p<0·0001) at a median MRSCA age of 5·0 years (IQR 2·1–12·5) before, and that this acquisition of ethambutol resistance was followed by resistance to pyrazinamide, ethionamide–prothionamide, fluoroquinolones, second-line injectables, or cycloserine (figure 3A), among which MRSCA ages did not significantly differ (figure 3B, appendix p 23). We found no significant correlation between the global median MRSCA dates and the drug’s first date of introduction into clinical use with *r*^2^=0·04 (linear regression, *F* test with 1 df p=0·60, appendix p 24).

**Figure 4:**
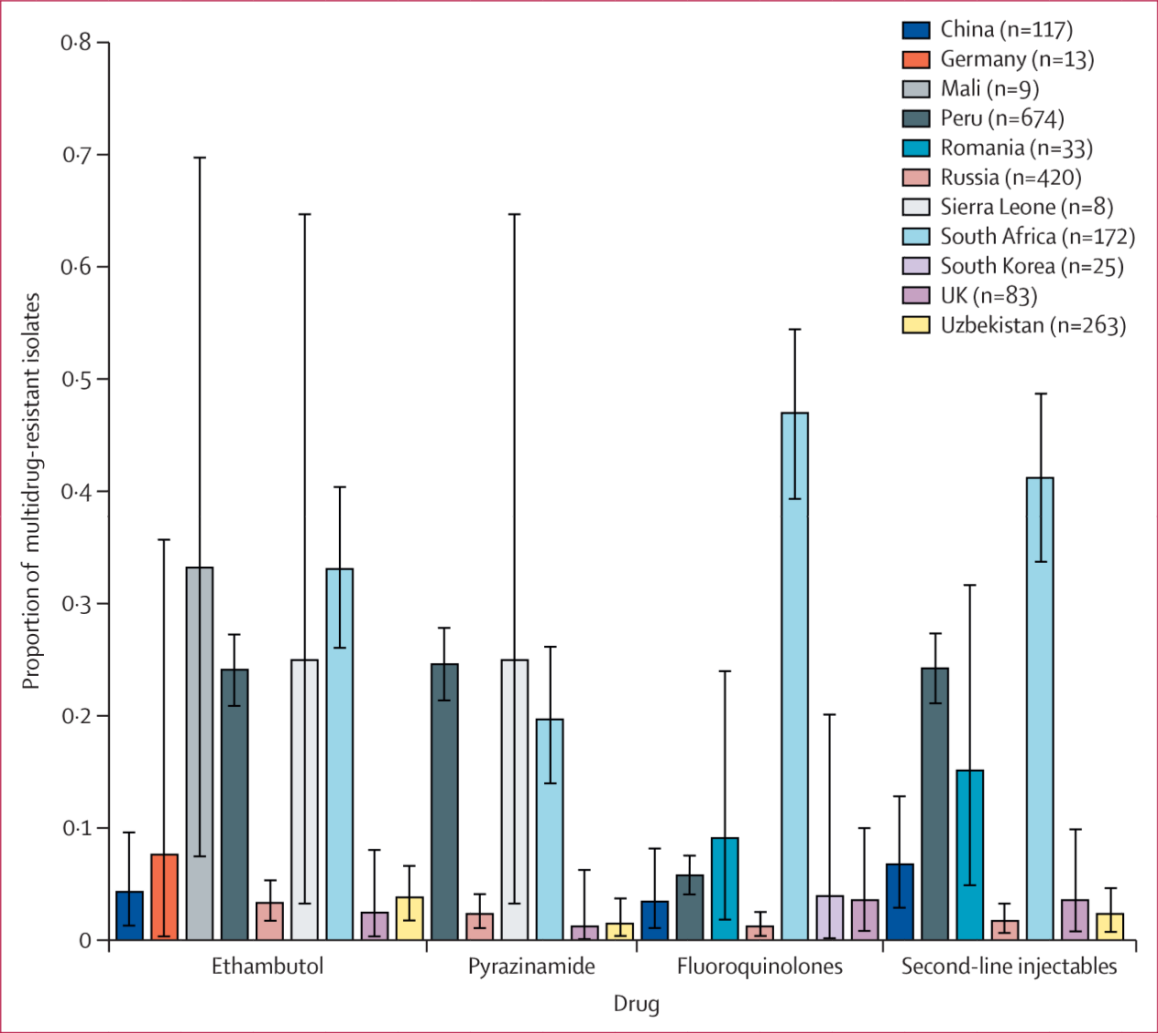
Proportion of multidrug-resistant isolates with recent amplification of resistance Resistance to ethambutol, pyrazinamide, fluoroquinolones, or second-line injectables by country (MRSCA age estimate <5 years ago) are shown. The key lists the number of multidrug-resistant isolates analysed from each country. Error bars indicate 95% CI. Four countries displayed a measurable proportion of recent fluoroquinolone and second-line injectable amplification (95% CI does not include 0): China, Peru, Romania, and South Africa. MRSCA=most recent susceptible common ancestor.

We assessed the frequency of resistance amplification within 5 years of sample isolation (hereafter referred to as recent resistance amplification) to pyrazinamide, ethambutol, fluoroquinolones, and second-line injectables among multidrug-resistance (ie, to pre-extensive drug-resistance or extensive drug-resistance) within 5 years of sample isolation. Among the 11 countries with both multidrug-resistant and pre-extensive drug-resistant or extensively drug-resistant isolates, we identified four countries (Peru, Russia, Sierra Leone, South Africa) with recent resistance amplification to pyrazinamide and ethambutol (>1% of multidrug-resistant) among any lineage. Rates of recent amplification ranged from 2% (95% CI 1–4) for pyrazinamide in Russia to 33% (26–41) for ethambutol in South Africa (figure 4). Peru, Romania, and South Africa were also measured to have recent resistance amplification to fluoroquinolones and second-line injectables (figure 4). The median MRSCA age for fluoroquinolone or second-line injectable resistance among multidrug-resistant isolates was 4·7 years (IQR 1·9–9·8) before sample collection.

**Figure 5:**
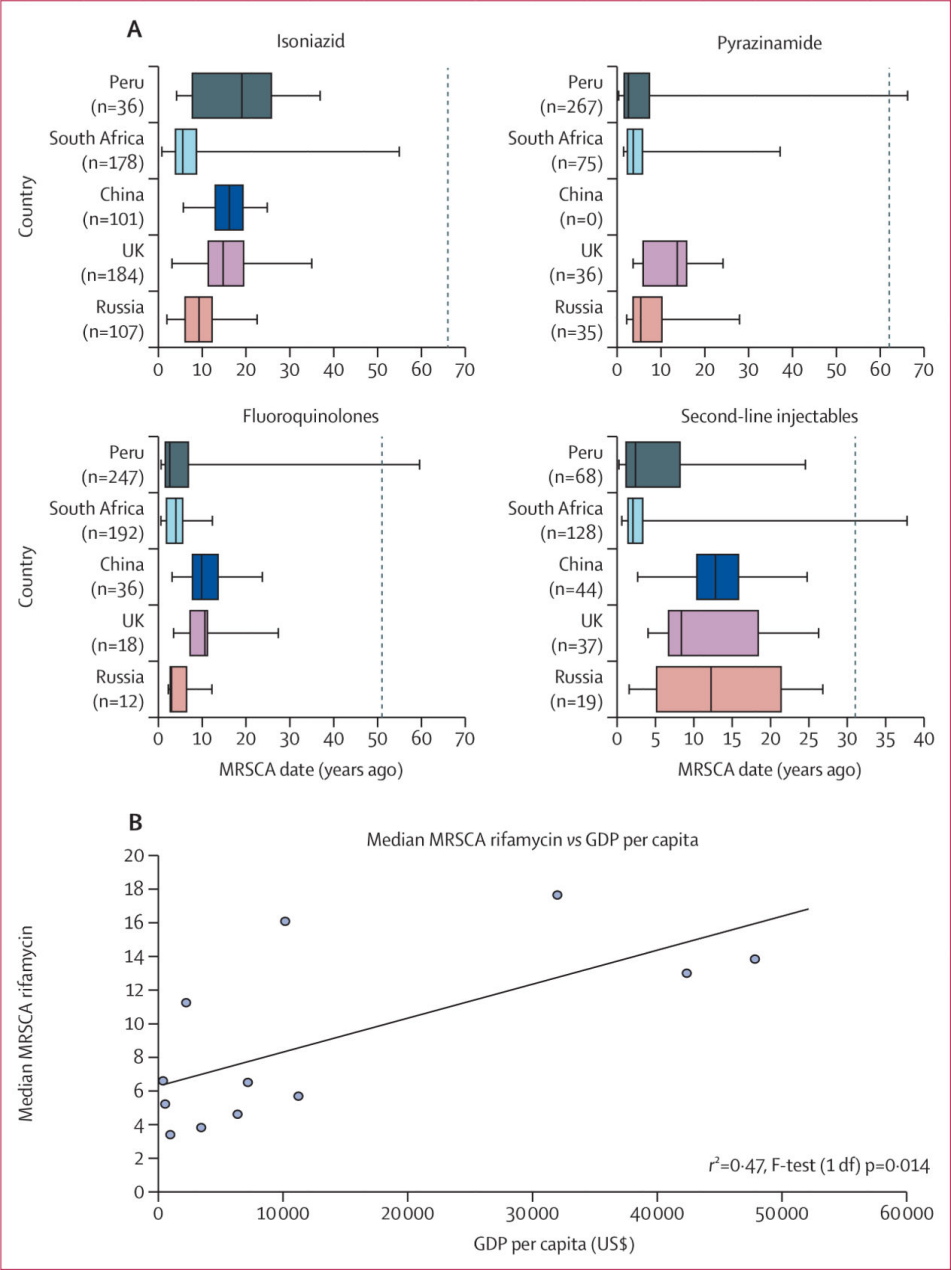
MRSCA distribution per country and median rifamycin MRSCA date versus GDP per capita for 12 countries Dotted vertical line indicates year when drug was introduced (appendix p 25). Data plotted are provided in the appendix (p 28) and include drugs other than rifamycins. MRSCA=most recent susceptible common ancestor. GDP=gross domestic product.

We found rifamycins to have the highest proportion of old resistance (MRSCA >20 years before isolation) at 17%, 197 of 1184 of the total dated rifamycin resistance across countries and lineages. Old resistance was well distributed geographically, and for rifamycins occurred in nine of 12 countries with available dating data (figure 5A, appendix p 25). Old fluoroquinolone resistance constituted 8% (24 of 311) of total dated isolates and spanned six of the seven countries with available data.

We compared the geographical distribution of MRSCA ages restricted to four key drug classes, namely isoniazid, rifamycins, second-line injectables, and fluoroquinolones, and the five countries with the largest number of resistant isolates from any lineage (figure 5A, appendix p 25). MRSCA ages did not differ between China and the UK across all four drug classes (Wilcoxon raw p>0·024). These two countries had the oldest median MRSCA across the five countries and four drug classes except for isoniazid (figure 5A, appendix p 25). South Africa most consistently had the youngest median MRSCA for the four drug classes, but its MRSCA distribution was not significantly different from that of Peru (for fluoroquinolones and second-line injectables) and Russia (for second-line injectables; Wilcoxon raw p>0·016, appendix p 26). A similar geographical and age pattern was observed for pyrazinamide and ethambutol across these five countries (appendix p 26).

We examined if geographical resistance age differences correlated with resources available for tuberculosis control programmes using the gross domestic product per capita as a proxy. We found gross domestic product to correlate significantly with an older rifamycin MRSCA date with *r*^2^=0·47 (linear regression, *F* test with 1 df p=0·014; figure 5B, appendix p 28).

We assessed the frequency of 267 resistance mutations previously determined to be important for resistance prediction^16^ and their geographical distribution among the 8550 isolates with country of origin and WGS data meeting quality criteria (figure 1). We pooled results across lineages in each country. Resistance mutation prevalence varied significantly by country. The most frequent isoniazid causing mutation,^18^ *katG* Ser315Thr, was more frequent among phenotypically isoniazid-resistant isolates by drug susceptibility tests (pheno-R) in Russia (444 [84%] of 526) than in Peru (510 [67%] of 760; Fisher’s exact test p<0·0001). The second most common isoniazid resistance mutation, −15 cytosine to thymine *fabG1-inhA* promoter, was more prevalent among isoniazid pheno-R Peruvian isolates (20%) than in Russian isolates (8%; Fisher’s exact test p<0·0001). 24 (9% [SD 11], frequency range 0–39) of 267 resistance mutations varied geographically to a larger extent than the mutation *fabG/inhA* promoter −15 cytosine to thymine (appendix p 31). The mutation Ile491Phe was described in 2017 to be common in Eswatini^19^ and is not detectable by Hain Line-probe or GeneXpert commercial molecular diagnostics. In our sample that did not contain data from Eswatini, we calculated an SD of 1% (range 0–4) for the global frequency of Ile491Phe among rifamycin pheno-R isolates.

We calculated the proportion of pheno-R isolates that can be captured by the Hain Line-probe or GeneXpert commercial molecular diagnostics due to the presence of one or more mutations in their pooled target regions for the drugs isoniazid, rifamycins, second-line injectables, and fluoroquinolones (table; appendix p 34). Second-line sensitivity differed significantly across countries (p<0·0003; appendix p 34). Fluoroquinolone sensitivity in Peru was 38% (46 of 121) and 77% (86 of 111) in South Africa (Fisher’s exact test p=1 × 10^−^⁶). A similarly low sensitivity for second-line injectable resistance was seen in Peru compared with South Africa (Fisher’s exact test p=3 × 10^−^⁴; appendix p 34).

Refining the commercial resistance mutation list to include variants characterised in diverse global *M tuberculosis* whole genome data using direct association^8^ or randomForests^16^ might improve sensitivity and specificity. We found this select-WGS approach to improve sensitivity slightly for isoniazid and second-line injectables with relatively preserved specificity (table). In addition, this approach allowed prediction for drugs not tested by commercial diagnostics—pyrazinamide, ethambutol, and streptomycin. For comparison, we assessed if including any non-silent variant in the resistance regions (excluding a select number of known lineage markers) was indeed inferior to the more informed select WGS test reported previously. We found that the all WGS test only modestly improved sensitivity and at the expense of a larger decrease in specificity (table).

## Discussion

Using 8550 clinical *M tuberculosis* sequences with culture-based drug susceptibility tests, we examined geographical trends in the drug-resistant tuberculosis epidemic. Geographically, *M tuberculosis* lineages 1–4 were each represented in the continents of Africa, Asia, and Europe, providing evidence of disease spread across borders, likely to be driven by human migration.^17^ We found multidrug-resistant tuberculosis in nearly every country represented by more than ten isolates. Extensively drug-resistant isolates were found in half of these countries and spanned all five major continents. Lineage 2 had the highest percentage of multidrug-resistant isolates in our sample followed by lineages 4, 3, and 1. Using this diverse sample, we dated more than 4869 resistance phenotypes across four lineages and 15 countries.

We found a consistent order of resistance acquisition globally among drug classes. The development of isoniazid resistance was previously found to be a sentinel event heralding the development of multidrug resistance.^20^ Our results corroborate these findings using phenotypic resistance data across a larger, geographically diverse sample. After isoniazid, we found that *M tuberculosis* acquires resistance to rifamycins or streptomycin, then ethambutol, followed by pyrazinamide, ethionamide–prothionamide, fluoroquinolones, second-line injectables, or cycloserine. We found no correlation between the age of resistance acquisition and the year of clinical introduction of the drug but there might be multiple other causes for the observed order of resistance acquisition. Differences in mutation rates across drug targets or resistance genes have been postulated but shown to be an unlikely explanation for isoniazid resistance arising first.^21^ Pharmacokinetic differences might result in higher risk for underdosing for some drugs and earlier resistance acquisition.^22^ Bacterial fitness costs are also variable across resistance mutations. For isoniazid resistance, mutations like *katG* Ser315Thr carry a low fitness cost and probably contribute to resistance arising earliest for this drug.^20^ The order of drug administration can explain dating differences between first-line (isoniazid, rifamycins, ethambutol, pyrazinamide) and second-line (ethionamide–prothionamide, fluoroquinolones, second-line injectables) or third-line (cycloserine) resistance, as second-line drugs are usually only administered after resistance to first-line drugs is ascertained. Acquisition of resistance to isoniazid first and then rifamycins might also relate to their use for treatment of latent tuberculosis infection, leading to more exposure and selection pressure overall. However, because adoption of isoniazid preventive therapy for latent tuberculosis remains low in many parts of the world, we expect it to be a lesser contributor to isoniazid and rifamycin resistance.^23^ Lastly, the observation of contemporaneous acquisition of rifamycin and streptomycin resistance is probably best explained by the effects of category II tuberculosis treatment initially recommended in 1991.^24^ Category II is no longer recommended by WHO but consists of adding streptomycin to the first-line drug regimen after treatment failure. Our dating supports that streptomycin resistance amplified among patients whose treatment was unsuccessful due to recent rifamycin resistance or multidrug-resistant acquisition, or both.

Published evidence supports the idea that most resistant cases of *M tuberculosis* result from recent resistance acquisition in the host or are related to transmission.^25^ Thus, the identification of isolates with old resistance in most countries with available data suggests high fitness for continued transmission between human hosts. This hypothesis is also supported by the finding of a lower bound of tuberculosis resistance due to transmission at 14–52% across countries with available data. As our approach cannot distinguish between resistance importation through human migration after transmission outside of the country and new resistance acquisition, these figures are underestimates of the true resistance burden due to transmission. Tuberculosis mathematical models have previously predicted transmission to be a major driver of observed resistance rates and have emphasised that drug resistant strain fitness is a key parameter dictating how the resistance epidemic will unfold.^26^ Our results support that some resistant isolates are fit and successfully transmitting patient-to-patient, sometimes uninterrupted, for more than 20 years. These data emphasise the need to contain resistance transmission through improved diagnosis, treatment, and other preventive strategies such as infection control and vaccine development.

In addition to transmission, we find evidence for recent resistance amplification, especially to second-line drugs mediating the transition from multidrug-resistant to extensively drug-resistant tuberculosis. Extensive drug resistance has considerably worse treatment outcomes than susceptible tuberculosis and incurs more than 25 times the cost.^27^ We estimate that half of fluoroquinolones and second-line injectable resistant isolates had acquired resistance within 4·7 years of isolation despite the promotion of directly observed therapy by WHO since 1994. As most fluoroquinolones and second-line injectable resistant isolates are also multidrug-resistant, our results also emphasise the need for better regimens to treat multidrug resistance that can prevent resistance amplification. By country, we found a significant correlation between the estimated age of resistance acquisition and per capita gross domestic product, with more affluent countries having older ages of resistance. This correlation is likely to be driven by a combination of factors, but the routine use of drug susceptibility tests and close patient monitoring in well resourced health systems are probably important contributors. Specifically, we found that China and the UK had the oldest resistance ages across the drugs. The Chinese national tuberculosis programme budget was increased from US$98 million in 2002 to $272 million in 2007^28^ and a new policy for free tuberculosis diagnostic tests and drug use was introduced in 2004.^29^ This increased investment can explain the observed low rates of recent resistance acquisition in China.^17^

Probably due to geographical differences in *M tuberculosis* lineage, transmission, and resistance acquisition rates, we found 10% of assayed resistance mutations to have high geographical variance. We also found commercial diagnostics to vary in sensitivity for second-line drugs. Given reports from 2018 confirming the accuracy of WGS for predicting *M tuberculosis* susceptibility,^8^ we measured improvements in resistance sensitivity offered by including mutations outside of regions targeted by commercial diagnostics through direct association. This approach offered modest improvements in sensitivity with little to no change in specificity. We found a considerable number of indeterminate mutations in resistance regions, which when included with simple direct association improve sensitivity, but at the expense of loss of specificity. The study of these variants through statistical models will probably further inform their diagnostic use in the future.^16^

Our study has several limitations including the oversampling of drug-resistant isolates as evidenced by our comparison with WHO reported multidrug-resistant rates. We tried to control for this factor by dating only in countries with at least 20% susceptible isolates and limiting dating of low diversity samples that represented unique outbreak settings and did not have long-term information about resistance acquisition. This method might have resulted in underestimation of rates of recent resistance acquisition (within 5 years of sample isolation), but despite this possibility we were able to document recent resistance acquisition in many countries. Molecular dating is also reliant on the accurate estimation of the phylogenetic tree of *M tuberculosis* isolates and the molecular clock assumption. We thus used a rigorous approach to phylogenetic estimation and dating despite its computational and time cost.^30^ Another limitation of our study is the scarcity of data for immigration or disease importation. For this reason, we avoided drawing conclusions concerning specific countries, and instead we comment on trends spanning multiple countries expected to have a range of tuberculosis burden due to importation. The absence of these data also limited our ability to assess disease transmission across borders. Our analysis also assumed accuracy of culture-based phenotypic drug susceptibility tests, even though test to test variability is known to exist. We justify this assumption as our data was curated from ReseqTB,^14^ and studies in which phenotypic testing was done in national or supranational laboratories with rigorous quality control.

In conclusion, this descriptive analysis in a large convenience sample of *M tuberculosis* WGS supports that resistance is fuelled by both recent acquisition and ongoing transmission, and suggest the need for better detection, treatment, and health system investment. In the future, the observed patterns can be reassessed further with isolate WGS data now systematically generated as a by-product of tuberculosis surveillance and resistance diagnosis.^8^

## Supplementary Material

1

## Figures and Tables

**Table:** Sensitivity and specificity of commercial and WGS-based tests for resistance diagnosis

	Commercial test		randomForests-select WGS test		Direct association-select WGS test		All WGS test*	
	Sensitivity	Specificity	Sensitivity	Specificity	Sensitivity	Specificity	Sensitivity	Specificity
Isoniazid	83% (2759/3306)	93% (4834/5201)	88% (2900/3306)	92% (4780/5201)	89% (2956/3306)	92% (4776/5201)	92% (3029/3306)	64% (3314/5201)
Rifamycins	90% (2354/2624)	93% (5361/5786)	91% (2385/2624)	92% (5341/5786)	91% (2395/2624)	92% (5338/5786)	92% (2405/2624)	89% (5178/5786)
Fluoroquinolones	53% (452/854)	94% (2626/2790)	51% (439/854)	95% (2637/2790)	52% (440/854)	95% (2641/2790)	57% (488/854)	86% (2406/2790)
Second-line injectables	56% (517/921)	86% (2179/2547)	58% (535/921)	84% (2136/2547)	57% (524/921)	86% (2185/2547)	64% (594/921)	80% (2027/2547)
Pyrazinamide	··	··	65% (862/1324)	95% (4660/4907)	75% (996/1324)	91% (4485/4907)	78% (1030/1324)	89% (4384/4907)
Ethambutol	··	··	79% (1476/1863)	86% (4702/5441)	75% (1389/1863)	88% (4772/5441)	85% (1589/1863)	74% (4042/5441)
Streptomycin	··	··	76% (1601/2112)	86% (3204/3726)	77% (1627/2112)	87% (3223/3726)	91% (1928/2112)	71% (2657/3726)

Sensitivity is the percentage of resistant isolates classified as resistant. Specificity is the percentage of susceptible isolates classified as susceptible. WGS=whole genome sequencing.

* Classifying fluoroquinolones and second-line injectables as susceptible if no isoniazid and no rifamycin resistance mutation found.

## Research in context

### Evidence before this study

Acquisition and spread of drug-resistance by *Mycobacterium tuberculosis* varies across countries. Local factors driving evolution of drug resistance in *M tuberculosis* are not well studied. We searched PubMed for the following MeSH terms: “tuberculosis”, “antibacterial drug resistance”, “communicable disease transmission”, and “clonal evolution” with no date restrictions up until Jan 5, 2021. The search yielded no results, which suggested that local factors driving evolution of drug resistance in *M tuberculosis* are not well studied.

### Added value of this study

We applied molecular dating to 6099 global *M tuberculosis* patient isolates and found the order of resistance acquisition to be consistent across the countries examined (ie, acquisition of isoniazid resistance first followed by rifampicin and streptomycin followed by resistance to other drugs). In all countries with data available, there was evidence for transmission of resistant strains from patient to patient and in the majority for extended periods of time (>20 years). Countries with lower gross wealth indices were found to have more recent resistance to the drug rifampicin. Based on the resistance patterns identified in our study, we estimate that commercial diagnostic tests vary considerably in sensitivity for second-line resistance diagnosis by country.

### Implications of all the available evidence

The longevity of resistant *M tuberculosis* in many parts of the world emphasises its fitness for transmission and its continued threat to public health. The association between country wealth and recent resistance acquisition emphasises the need for continued investment in tuberculosis care delivery and surveillance programmes. Geographically relevant diagnostics that take into account a country’s unique distribution of resistance are necessary.
