# Supplementary material for: Globally diverse Mycobacterium tuberculosis resistance acquisition: a retrospective geographical and temporal analysis of whole genome sequences
Source: Lancet Microbe. Author manuscript; Available in PMC 2021 Apr 27. (PMC8078851; doi:10.1016/s2666-5247(20)30195-6)
Supplement: 1 [file NIHMS1679671-supplement-1.pdf]

THE LANCET
Microbe

Supplementary appendix

This appendix formed part of the original submission and has been peer reviewed. We post it as supplied by the authors.

Supplement to: Ektefaie Y, Dixit A, Freschi L, et al. Globally diverse *Mycobacterium tuberculosis* resistance acquisition: a retrospective geographical and temporal analysis of whole genome sequences. *Lancet Microbe* 2021; published online January 27. https://doi.org/10.1016/S2666-5247(20)30195-6.

Supplementary Methods:

Data curation and quality control:

The isolate metadata including their geographic locations were downloaded using metatools_ncbi (https://github.com/farhat-lab/metatools_ncbi). We also performed literature curation to fill the gaps in the NCBI geographic location data. The resulting table of the geographic locations of the isolates is available in Supplementary File 1. We excluded isolates that did not meet WGS quality control criteria as detailed below, had no geographic information or were not tested for phenotypic resistance to one or more drugs.

Genomic analysis/variant calling

We used a previously validated genomic analysis pipeline for MTB described by Ezewudo *et al.* (1) with modifications as detailed in the supplement.

Genomic analysis/variant calling:

Briefly, reads were trimmed using PRINSEQ(2) setting average phred score threshold to 20. Raw read data was confirmed to belong to MTB complex using Kraken(3). Isolates with <90% mapping were excluded. Reads were aligned to H37Rv (GenBank NC000962.3) reference genome using BWA MEM(4). Duplicate reads were removed using PICARD(5). We excluded any isolates with coverage <95% of known drug resistance regions (*katG*, *inhA* & its promoter, *rpoB*, *embA, embB, embC* & *embB* promoter, *ethA*, *gyrA* and *gyrB*, *rrs*, *rpsL*, *gid*, *pncA*, *rpsA*, *eis* promoter) at 10x or higher. Variants were called using Pilon(6) that uses local assembly to increase indel (insertions and deletions) call accuracy. This deviates from Ezewudo *et al.* that uses Samtools for variant calling(1). The reference allele was implied if allele frequency was <75% or the Pilon filter was not PASS. Low confidence coordinates were filtered from all strains if >95% of strains did not have coverage of (trimmed reads) at least 10x at that site. Isolate lineage belonging to one of the seven main MTB lineages was confirmed using the Coll *et al.* SNP barcode(7).

Drug resistance definitions and comparison with WHO reported resistance proportions:

The 'Mono' resistant designation was given to isolates that were resistant to only one specific drug and susceptible to all others that were tested, with the exception of the INH-mono resistant category that encompassed any isolates that were resistant to INH and/or STR but not to others that were tested. The 'Other-R' category was reserved for isolates that were resistant to some drugs but were neither INH or STR mono resistant, nor MDR or XDR. Isolates were labelled susceptible if they were susceptible to all drugs tested.

To compute exact binomial confidence intervals for MDR proportions by country we used the python library statsmodel(8). To assess overlap with World Health Organization (WHO) estimates from the most recent year(9) we determined whether the confidence intervals of our proportion intersected with that of the WHO. We labelled our estimate as high if our confidence interval was higher than the WHO, low if it was lower, and the same if they intersected.

Estimating resistance acquisition dates and lower bounds of resistance transmission:

A maximum likelihood tree was generated for each group via RAxML 8.2.11(10) with H37Rv (NC000962.3) as the outgroup, starting from a neighbour-joining seed tree and assuming a generalized time reversible (GTR) nucleotide substitution model with the Γ distribution used to model site rate heterogeneity(11). We bootstrapped the maximum likelihood tree 1000 times. The maximum likelihood tree was dated using BEAST v1.10.4(12) assuming a relaxed molecular clock with a log normal distribution and a mean rate of 0.5 SNP per genome per year based on prior published data(13). Sumtrees.py from the DendroPy library(14) was then used to combine the output from the bootstrap analysis and that of BEAST to get our final dated phylogenetic tree with nodal bootstrap support.

We dated the most recent common ancestor between all the resistant isolates and their most closely related susceptible isolate. Accordingly, the dates of resistance acquisition will be referred to as the estimated age of the most recent *susceptible* common ancestor (MRSCA) in years prior to isolation of the clinical sample(s) throughout the text. We excluded resistant isolates with MRSCAs inferred at nodes with less than 50 bootstrap support.

We calculated the number of phylogenetically inferred resistance acquisition events (N_{aq}) per country and lineage as the number of unique MRSCAs identified. This was compared with the total resistant isolates that could be dated

(N_{td}). Phylogenetically inferred unique resistance acquisition events for a particular country may be related to either *in host* evolution of new resistance or due to human migration/importation from another country with the latter still possibly related to transmission elsewhere. Thus, the following quantity represents a lower bound on the burden of resistance due to transmission for a particular country:

$$(N_{td} - N_{aq})/N_{td}$$

To estimate the order of resistance acquisition for different drugs we pooled the MRSCA dates by drug across countries and lineages. We compared the medians of the MRSCA distributions and performed pairwise Wilcoxon Rank Sum tests to assess for statistical significance, correcting for multiple testing using the Bonferroni approach. Interquartile ranges were calculated using the python package numpy(15).

We correlated the median MRSCA date per drug pooled across countries against the date of drug introduction using linear regression as implemented in Microsoft Excel version 16.25.

Distribution of resistance mutations

We measured resistance mutations' geographic variance by calculating the proportion of resistant isolates with the resistance mutation per country, excluding countries with less than 10 resistant isolates for a particular drug. We computed the standard deviation of this distribution of proportions across countries. To test cross-country differences in the proportion of INH phenotypically resistant isolates that contained specific mutations, we used a Fisher-test using the python library scipy(16).

We used three resistance mutation lists to perform the resistance prediction. In each case, an isolate harboring one or more mutation from the resistance list was classified as resistant and otherwise as susceptible. "RF-Select: Random forest (RF) is a non-parametric classifier used to select variables most important for prediction. The method involves the construction of a multitude of decision trees and uses a majority vote across the predictions to create a classification. We have used this method previously to generate a list of mutations most important for resistance prediction(17). In contrast the "DA-select" list of mutations was determined based on their individual abundance among resistance and susceptible TB isolates. This list of mutation is also previously published and used in this present analysis as is(18),(19). The "All-WGS" list consists of any non-synonymous mutation or noncoding mutation in known DR regions.

GDP/Programmatic Spending

To correlate countries' gross domestic product (GDP) per capita against resistance acquisition dates, GDP per capita data for 2019 was gathered from the International Monetary Fund (IMF)(20) and plotted against the median MRSCA date for RIF using Microsoft Excel version 16.25. F-value and significance was calculated via Anova(21) in Excel.

Sensitivity analysis on diversity filter:

We conducted a sensitivity analysis dating resistance in the 20 groups that were excluded due to the diversity filter. Of the 20, 16 groups were of lineage 2 isolates in Russia (10 drugs) and Uzbekistan (6 drugs) likely representing recent chains of transmission. Beijing (Lineage 2) is thought to be a more transmissible TB lineage and is well documented to result in outbreaks in Central Asia and Russia among other places confirming our suspicion that removing the diversity filter will include data from outbreaks(22). After adding dates from the 20 groups, the overall median MRSCA age of INH, RIF, PZA, EMB, ETH, SLI, FLQ decreases as expected but modestly by -2.3, -1.5, -0.9, -1.5, -1.7, -0.5, -2.3 years respectively. This did not affect the order of resistance acquisition appreciably which remained INH>STR,RIF>EMB,PZA and other drugs. The one exception is that EMB dates are no longer statistically significantly different from PZA and other second line drug resistance. This may have resulted from including the groups representing MDR outbreak settings in Russia and Uzbekistan, and the small difference in median MRSCA between EMB and PZA.

Sensitivity analysis on molecular clock rate:

We reran dating in 142 groups of 179 country/lineage/drug groups with a mutation rate of 0.3 mutations/genome/year and investigated how MRSCA date distributions change. We were not able to run the

remaining 37 groups as these were the largest groups (in number of isolates) due to limitations in computational resources available.

Relative Drug Ordering: We investigated the relative ordering of drugs among the 142 groups that were rerun with a 0.3 mutation rate verses the original 0.5 mutation rate. Supplementary Table 17 shows the median MRSCA with IQR for various drugs. For every drug, changing the mutation rate to 0.3 led to an increase in median MRSCA.

We also investigated how changing the mutation rate lead to a change in the relation between the MRSCA distributions of different drugs. In Supplementary Table 18, we show the p-value for the Wilcoxon Rank-Sum Test between the MRSCA distributions of various drugs for each mutation rate. Changing the mutation rate did not change the inferred order drug resistance acquisition.

MRSCA Date Distribution: We visualized the MRSCA age distribution for PZA, EMB, FLQ, and SLIs in the three countries with the most MDR isolates (Peru, South Africa, and China) (Supplementary Figure 3). Changing the mutation rate shifted the distribution for MRSCA dates rightwards to older ages but preserved its shape in most cases. Below is the description of recent resistance acquisition among MDR-TB isolates under the 0.3 mutation rate sensitivity analysis and the original 0.5 mutation rate analysis for comparison. Note we have incomplete data for the 0.3 mutation rate as stated above, results from the United Kingdom for PZA and EMB and for Peru for SLIs and EMB and a few other drug-country combinations are missing.

For 0.3 Mutation Rate: Among the 9 countries with both MDR and pre-XDR/XDR isolates, we identified five countries (Mali, Peru, Russia, South Africa, Uzbekistan) with recent resistance amplification to PZA or EMB (>1% of MDR) among any lineage. Rates of recent amplification ranged from 10% (95% CI 6% - 16%) for South Africa in PZA to 20% (15% - 27%) for South Africa in EMB. Peru, Romania, South Africa, and Uzbekistan were measured to have recent resistance amplification to FLQs or SLIs. The median MRSCA age for FLQ or SLI resistance acquisition among MDR isolates was 7.9 years (IQR 3.3 – 16.2) prior to sample collection.

For 0.5 Mutation Rate: Among the 11 countries with both MDR and pre-XDR/XDR isolates, we identified eight countries (China, Mali, Peru, Russia, Sierra Leone, South Africa, United Kingdom, Uzbekistan) with recent resistance amplification to PZA or EMB (>1% of MDR) among any lineage. Rates of recent amplification ranged from 2% (95% CI 1% - 4%) for Russia in PZA to 33% (26% - 41%) for South Africa in EMB. China, Peru, Romania, Russia, South Africa, United Kingdom, and Uzbekistan were also measured to have recent resistance amplification to FLQs or SLIs. The median MRSCA age for FLQ or SLI resistance acquisition among MDR isolates was 5.0 years (IQR 1.9 – 10.0) prior to sample collection.

Although fewer countries were measured to have resistance acquisition (<5 years) to PZA, EMB, SLIs and FLQ as substantial proportion of recent resistance acquisition < 5 years was still measured and the median overall age of SLI or FLQ resistance amplification among MDR-TB only increased by 2.9 years from 5.0 years (IQR 1.9-10, 0.5 mutation rate) to 7.9 years (IQR 3.3-16.2, 0.3 mutation rate).

Geographic Differences: We investigated the relative difference in MRSCA distributions between China and Peru (drugs RIF and SLIs) with changing the mutation rate (Supplementary Table 19). For both mutation rates China had older MRSCA dates than Peru (Wilcoxon Rank Sum Test, P-value < 0.01).

GDP verses MRSCA: Using the 0.3 mutation rate we reproduced the relationship between GDP and MRSCA across the 142 groups (Linear Regression, F-test with 1 DF, 0.5 Mutation Rate P-value 0.006, 0.3 Mutation Rate P-value of 0.009) (Supplementary Table 20, Supplementary Figure 4).

Code

All code used in the analysis is publicly accessible at https://github.com/farhat-lab/geo_dist_tb.

Statistical analysis and significance thresholds

For testing we used Fisher exact tests (two binary variables), Wilcoxon Rank sum tests (binary vs continuous) and linear regression (two continuous variables). Across all tests we used a P-value threshold of 0.05 corrected for multiple testing using the Bonferroni method when multiple testing was performed.

Supplementary Material

Supplementary Table 1 Countries Present In Filtration

8550 Isolates passed sequence quality criteria
52 Countries

Azerbaijan, Bangladesh, Belarus, Brazil, Burma, Canada, China, Colombia, Democratic Republic of the Congo, Denmark, Djibouti, Dominican Republic, Estonia, Gambia, Georgia, Germany, Ghana, Guinea, India, Indonesia, Iran, Ireland, Kazakhstan, Malawi, Mali, Moldova, Morocco, Nepal, Netherlands, Nigeria, Pakistan, Peru, Philippines, Portugal, Romania, Russia, Rwanda, Sierra Leone, South Africa, South Korea, Spain, Swaziland, Sweden, Switzerland, Thailand, Turkmenistan, Uganda, United Kingdom, United Republic of Tanzania, United States of America, Uzbekistan, Vietnam

23 Countries with more than 10 isolates and phenotypic data for all isolates

Belarus, Canada, China, Germany, India, Iran, Malawi, Mali, Moldova, Netherlands, Peru, Romania, Russia, Sierra Leone, South Africa, South Korea, Swaziland, Thailand, Turkmenistan, Uganda, United Kingdom, United States of America, Uzbekistan

2451 in lineage-drug-country groups did not meet dating requirements

6099 Isolates included in molecular dating analysis
15 Countries

China, Germany, India, Malawi, Mali, Moldova, Netherlands, Peru, Romania, Russia, Sierra Leone, South Africa, South Korea, United Kingdom, Uzbekistan

Supplementary Table 2 Commercial Diagnostics(23),(24),(25),(26),(27):

Drug	Gene	Location
Isoniazid	katG codon	315
Isoniazid	inhA promoter	-15,-16,-8
Rifamycin	rpoB codons	424-454
Aminoglycosides	rrs	1401,1402
Aminoglycosides	eis	-10 to -14, -37
Fluoroquinolones	gyrA	89-94
Fluoroquinolones	gyrB	500-541

*E-coli 505-534

Supplementary Table 3 Genes Searched Per Drug.

Genes searched for resistant mutations in "All WGS Test" (AMK: amikacin, PAS: para-aminosalicylic acid, EMB: ethambutol, CAP: capreomycin, KAN: kanamycin, CIP: ciprofloxacin, INH: isoniazid, STR: streptomycin, RIF: rifampicin, LEVO: levofloxacin, ETH: ethionamide, OFLX: ofloxacin, PZA: pyrazinamide)

Drug	Genes Searched
AMK	rrs
PAS	thyA, inter-thyA-Rv2765, folC, inter-thyX-hsdS.1
EMB	embA, embB, embC, iniB, inter-embC-embA
CAP	rrs, tlyA
KAN	rrs, inter-eis-Rv2417c
CIP	gyrB, gyrA
INH	inhA, iniB, embB, inter-Rv1482c-fabG1, ahpC, inter-oxyR'-ahpC, inter-embC-embA, kasA, katG, fabG1
STR	gid, rpsL, rrs, inter-rrs-rrl
RIF	rpoB
LEVO	gyrB, gyrA
ETH	inhA, inter-Rv1482c-fabG1, ethA, inter-ethA-ethR
OFLX	gyrB, gyrA
PZA	inter-pncA-Rv2044c, pncA, rpsA

Supplementary Table 4 Global Lineage Distribution

	Lineage-1	Lineage-2	Lineage-3	Lineage-4	Lineage-5	Lineage-6	Lineage-7	Total
**Belarus**	0	87	0	46	0	0	0	135
**Canada**	0	0	0	17	0	0	0	165
**China**	0	132	2	35	0	0	0	170
**Germany**	0	22	0	1	0	0	0	857
**India**	5	9	5	0	0	0	0	52
**Iran**	0	16	3	1	0	0	0	22
**Malawi**	208	10	162	945	0	0	0	1427
**Mali**	0	0	0	37	0	0	0	37
**Moldova**	0	16	0	35	0	0	0	51
**Netherlands**	7	19	0	70	0	0	0	98
**Peru**	0	82	0	869	0	0	0	1098
**Portugal**	0	2	0	10	0	0	0	13
**Romania**	0	0	0	33	0	0	0	33
**Russia**	0	525	1	273	0	0	0	868
**Sierra Leone**	3	2	0	41	4	10	0	79
**South Africa**	8	144	23	427	0	0	0	974
**South Korea**	0	37	1	2	0	0	0	80
**Swaziland**	0	9	0	0	0	0	0	10
**Sweden**	2	11	4	11	0	0	0	28
**Thailand**	0	2	0	0	0	0	0	17
**Turkmenistan**	0	10	0	0	0	0	0	11
**Uganda**	2	3	18	50	0	0	0	80
**United Kingdom**	222	102	560	716	9	4	1	1873
**United States of America**	1	3	0	0	0	0	0	34
**Uzbekistan**	0	20	0	0	0	0	0	265
**Unknown**	37	81	35	792	1	0	0	1749
**Total**	495	1344	814	4411	14	14	1	10226

Supplementary Table 5 Global Resistance Distribution and Other Resistance Count

Abbreviations: Susceptible (S), Multi-drug Resistant (MDR), Extensively Drug Resistant (XDR), Isoniazid Mono Resistant (INH_MONO), Streptomycin Mono Resistant (STR_MONO), Other Resistant (OTHER_R), Sum With Data (SUM_W_DATA)

country	S	MDR	XDR	INH_MONO	STR_MONO	OTHER_R	NO_DATA	SUM	SUM_W_DATA	MDR/OTHER_R
**Belarus**	28	1	0	1	0	0	105	135	30	NA
**Canada**	163	0	0	2	0	0	0	165	165	NA
**China**	46	117	23	0	0	0	7	170	163	NA
**Germany**	749	13	0	40	34	14	7	857	850	0.9
**India**	22	13	0	2	2	0	13	52	39	NA
**Iran**	15	6	0	0	0	0	1	22	21	NA
**Malawi**	1302	7	0	90	1	3	24	1427	1403	2
**Mali**	20	9	0	6	0	0	2	37	35	NA
**Moldova**	38	2	0	0	0	0	11	51	40	NA
**Netherlands**	68	0	0	5	20	0	5	98	93	NA
**Not Provided**	179	646	138	90	6	109	719	1749	1030	6
**Peru**	94	674	86	34	5	134	157	1098	941	5
**Romania**	0	33	6	0	0	0	0	33	33	NA
**Russia**	261	420	42	80	17	76	14	868	854	5.5
**Sierra Leone**	41	8	0	7	13	10	0	79	79	0.8
**South Africa**	612	172	102	34	8	56	92	974	882	3
**South Korea**	16	25	21	0	0	0	39	80	41	NA
**Swaziland**	8	1	0	0	0	1	0	10	10	1
**Thailand**	1	15	0	0	0	0	1	17	16	NA
**Turkmenistan**	3	1	0	3	1	3	0	11	11	0.3
**Uganda**	10	45	0	1	0	2	22	80	58	23

**United Kingdom**	1525	83	4	187	5	50	23	1873	1850	1.7
**United States of America**	0	29	1	0	0	1	4	34	30	29
**Uzbekistan**	0	263	3	1	0	1	0	265	265	263
**Total**	5201	2583	426	583	112	460	1246	10185	8939	5.6

Abbreviations: RIF (Rifamycin), ETH (Ethionamide), FQ (Fluoroquinolones), SLIS (Second Line Injectable), "_" (AND—EX: RIF_ISONIAZID_ETH = Isolate resistant to Rifamycin, Isoniazid, and Ethionamide)

**Other Resistance Categories**	**Count**
RIF	78
PYRAZINAMIDE	65
ETHAMBUTOL	44
ISONIAZID_ETHAMBUTOL_STREPTOMYCIN	29
ISONIAZID_ETHAMBUTOL	28
ISONIAZID_PYRAZINAMIDE	21
ISONIAZID_PYRAZINAMIDE_STREPTOMYCIN	16
ISONIAZID_ETHAMBUTOL_PYRAZINAMIDE_STREPTOMYCIN	11
RIF_ETHAMBUTOL	11
RIF_STREPTOMYCIN	10
ISONIAZID_STREPTOMYCIN_ETH	9
ISONIAZID_ETHAMBUTOL_FQ	9
SLIS	8
ISONIAZID_STREPTOMYCIN_FQ	8
ETHAMBUTOL_FQ	7
FQ	7
ISONIAZID_ETHAMBUTOL_STREPTOMYCIN_FQ	7
ETHAMBUTOL_STREPTOMYCIN	6
ISONIAZID_ETH	6
STREPTOMYCIN_FQ	5
ISONIAZID_ETHAMBUTOL_PYRAZINAMIDE_ETH	5
ISONIAZID_FQ	4
ISONIAZID_ETHAMBUTOL_STREPTOMYCIN_ETH	4
ISONIAZID_ETHAMBUTOL_PYRAZINAMIDE_STREPTOMYCIN_SLIS_ETH	3
ISONIAZID_ETHAMBUTOL_PYRAZINAMIDE_STREPTOMYCIN_SLIS	3
ISONIAZID_PYRAZINAMIDE_ETH	3
ETHAMBUTOL_PYRAZINAMIDE	3
RIF_PYRAZINAMIDE_STREPTOMYCIN	2
RIF_ETHAMBUTOL_PYRAZINAMIDE_STREPTOMYCIN_FQ	2
ISONIAZID_ETHAMBUTOL_PYRAZINAMIDE_STREPTOMYCIN_ETH	2
PYRAZINAMIDE_STREPTOMYCIN	2
RIF_ETHAMBUTOL_STREPTOMYCIN	2
ISONIAZID_SLIS	2
PYRAZINAMIDE_SLIS	2
ISONIAZID_ETHAMBUTOL_PYRAZINAMIDE_SLIS_ETH	2
ISONIAZID_ETHAMBUTOL_PYRAZINAMIDE	2

PYRAZINAMIDE_FQ	1
ISONIAZID_PYRAZINAMIDE_FQ_SLIS	1
STREPTOMYCIN_SLIS	1
RIF_ETHAMBUTOL_FQ	1
ISONIAZID_ETHAMBUTOL_STREPTOMYCIN_SLIS_ETH	1
ISONIAZID_ETHAMBUTOL_STREPTOMYCIN_SLIS	1
RIF_ETHAMBUTOL_STREPTOMYCIN_SLIS_ETH	1
ETH	1
RIF_PYRAZINAMIDE_FQ	1
RIF_ETHAMBUTOL_PYRAZINAMIDE_FQ	1
RIF_ETHAMBUTOL_PYRAZINAMIDE_STREPTOMYCIN_ETH	1
RIF_PYRAZINAMIDE	1
ETHAMBUTOL_ETH	1
ISONIAZID_ETHAMBUTOL_ETH	1
RIF_ETHAMBUTOL_SLIS	1
ISONIAZID_PYRAZINAMIDE_STREPTOMYCIN_ETH	1
RIF_PYRAZINAMIDE_SLIS_ETH	1
RIF_ETHAMBUTOL_PYRAZINAMIDE	1
RIF_ETHAMBUTOL_PYRAZINAMIDE_STREPTOMYCIN	1
ISONIAZID_ETHAMBUTOL_FQ_SLIS	1
ETHAMBUTOL_STREPTOMYCIN_FQ	1
PYRAZINAMIDE_SLIS_ETH_PAS	1
ISONIAZID_STREPTOMYCIN_FQ_SLIS_ETH_PAS	1
ISONIAZID_STREPTOMYCIN_SLIS_PAS	1
ISONIAZID_ETHAMBUTOL_SLIS	1
RIF_ETHAMBUTOL_PYRAZINAMIDE_FQ_SLIS_ETH	1
RIF_FQ_SLIS	1
ISONIAZID_PYRAZINAMIDE_SLIS	1
ISONIAZID_ETHAMBUTOL_PYRAZINAMIDE_SLIS_ETH_PAS	1
RIF_ETHAMBUTOL_PYRAZINAMIDE_SLIS	1
RIF_PYRAZINAMIDE_STREPTOMYCIN_FQ_SLIS	1
ISONIAZID_ETHAMBUTOL_PYRAZINAMIDE_SLIS	1
RIF_SLIS	1
RIF_FQ	1
	462

Supplementary Table 6 MDR Frequency Comparison:

Comparison of MDR Frequency between our estimate based on WGS data (WGS MDR Frequency) and estimate based on WHO data (WHO MDR Frequency). Number of isolates with resistance data per country is provided in the "Total Number of Isolates" column.

	WGS MDR Frequency	WHO MDR Frequency	Total Number of Isolates
**Belarus**	0.03±0.07	0.7(0.67-0.75)	30
**Canada**	0	0.01(0.01-0.02)	165
**China**	0.7±0.07	0.08(0.07-0.09)	163
**Germany**	0.02±0.01	0.03(0.02-0.05)	850
**India**	0.3±0.15	0.05(0.04-0.06)	39
**Iran**	0.3±0.2	0.02(0.01-0.02)	21
**Malawi**	0.01±0.01	0.01(0.01-0.02)	1403
**Mali**	0.26±0.14	0.03(0.02-0.04)	35
**Moldova**	0.05±0.07	0.3(0.31-0.36)	40
**Netherlands**	0	0.02(<0.01-0.01)	93
**Not Provided**	0.6±0.03		1030
**Peru**	0.7±0.03	0.09 (0.09-0.1)	941
**Romania**	1	0.05(0.05-0.06)	33
**Russia**	0.5±0.03	0.4(0.43-0.44)	854
**Sierra Leone**	0.1±0.07	0.03(0.02-0.04)	79
**South Africa**	0.2±0.03	0.04(0.04-0.05)	882
**South Korea**	0.6±0.15	0.04(0.03-0.04)	41
**Swaziland**	0.1±0.2	0.1(0.09-0.11)	10
**Thailand**	0.9±0.1	0.03(0.03-0.04)	16
**Turkmenistan**	0.1±0.2	0.2(0.19-0.24)	11
**Uganda**	0.8±0.1	0.02(0.02-0.03)	58
**United Kingdom**	0.04±0.01	0.02(0.01-0.02)	1850
**United States of America**	1.0±0.06	0.02(0.01-0.02)	30
**Uzbekistan**	1.0±0.01	0.3(0.29-0.34)	265

Supplementary Figure 7: Number of dates, number of strains, minimum and maximum date for each group used in MRSCA date analysis.

Legend: FLQ (Moxifloxacin, Ciprofloxacin, Ofloxacin), SLIs (Kanamycin, Amikacin, Capreomycin), RIF (Rifampicin, Rifabutin), ETH (Ethionamide, Prothionamide), CYS (Cycloserine), EMB (Ethambutol), PAS (Para-Aminosalicylic Acid), STR (Streptomycin), INH (Isoniazid), NIC (Nicotinamide)

Country	Lineage	Drug	DateMin	DateMax	Ndates	Nstrains
China	lineage2	CAPREOMYCIN	3.45984093	15.982453	14	120
China	lineage2	ETHAMBUTOL	3.37731625	24.2409758	46	126
China	lineage2	ETHIONAMIDE	3.35009983	24.8530463	28	109
China	lineage2	ISONIAZID	5.51074158	25.0054562	81	126
China	lineage2	KANAMYCIN	3.37401405	23.8914778	14	117
China	lineage2	OFLOXACIN	3.58212906	24.8460839	37	120
China	lineage2	RIFAMPICIN	4.83828982	24.748597	81	126
China	lineage2	STREPTOMYCIN	3.22660519	24.9597243	62	126
China	lineage4	CAPREOMYCIN	2.95305776	23.3148212	4	35
China	lineage4	ETHAMBUTOL	2.39254698	23.7901886	12	35
China	lineage4	ETHIONAMIDE	13.0237521	17.5053894	2	34
China	lineage4	ISONIAZID	8.80960097	24.1444241	20	35
China	lineage4	KANAMYCIN	2.98842916	19.172193	4	35
China	lineage4	OFLOXACIN	2.57515411	22.5217921	7	35
China	lineage4	RIFAMPICIN	8.52590981	22.0241404	20	35
China	lineage4	STREPTOMYCIN	3.69504275	22.9709794	9	35
Germany	lineage1	ISONIAZID	11.2961235	11.2961235	1	47
Germany	lineage1	PYRAZINAMIDE	13.2689486	13.2689486	1	47
Germany	lineage1	STREPTOMYCIN	11.6075157	11.6075157	1	47
Germany	lineage2	ETHAMBUTOL	4.65720301	10.9511898	4	56
Germany	lineage2	ISONIAZID	2.38996003	17.1780776	14	56
Germany	lineage2	PROTHIONAMIDE	9.43255204	22.5274111	2	17
Germany	lineage2	PYRAZINAMIDE	6.32326996	15.1863115	4	55
Germany	lineage2	RIFAMPICIN	3.86662799	17.364989	7	56
Germany	lineage2	STREPTOMYCIN	2.79105154	22.866233	14	55
Germany	lineage3	ISONIAZID	4.51026625	35.3640257	6	55
Germany	lineage3	STREPTOMYCIN	4.75424235	36.307397	6	55
Germany	lineage4	ETHAMBUTOL	21.8805457	21.8805457	1	581
Germany	lineage4	ISONIAZID	2.03159091	55.1685886	20	580
Germany	lineage4	PYRAZINAMIDE	27.4700561	28.1184751	2	580
Germany	lineage4	RIFAMPICIN	8.63846225	8.63846225	1	582
Germany	lineage4	STREPTOMYCIN	1.71517382	51.570326	29	581
India	lineage2	ETHAMBUTOL	10.3346905	26.8143034	5	13

India	lineage2	RIFAMPICIN	10.0781515	25.1249811	9	13
India	lineage2	STREPTOMYCIN	12.0283797	25.9637928	6	13
Malawi	lineage1	ISONIAZID	13.396797	19.1215413	7	188
Malawi	lineage1	STREPTOMYCIN	4.19097854	16.765887	6	46
Malawi	lineage3	ISONIAZID	10.8912891	18.7337636	6	147
Malawi	lineage3	RIFAMPICIN	5.41310346	5.41310346	1	147
Malawi	lineage4	ISONIAZID	3.37549005	12.1101526	24	860
Malawi	lineage4	PYRAZINAMIDE	5.00451284	6.70174994	3	181
Malawi	lineage4	RIFAMPICIN	5.44611036	6.62057578	4	860
Malawi	lineage4	STREPTOMYCIN	4.6566478	55.6670933	17	210
Mali	lineage4	ETHAMBUTOL	2.72404297	34.6278714	5	22
Mali	lineage4	ISONIAZID	4.97300099	42.3524858	12	24
Mali	lineage4	RIFAMPICIN	2.46719771	33.7581211	5	23
Mali	lineage4	STREPTOMYCIN	2.46170816	42.8394983	8	21
Moldova	lineage4	ETHAMBUTOL	1.5340503	3.86485636	6	34
Moldova	lineage4	RIFAMPICIN	2.82848387	4.56603418	7	34
Netherlands	lineage4	ISONIAZID	0.75516493	15.7722802	3	60
Netherlands	lineage4	STREPTOMYCIN	7.31797932	15.7456994	5	60
Peru	lineage2	AMIKACIN	5.85239501	23.7060501	5	68
Peru	lineage2	CAPREOMYCIN	12.2962929	59.7518977	6	67
Peru	lineage2	ETHAMBUTOL	0.84233477	23.3368004	29	71
Peru	lineage2	ETHIONAMIDE	3.15412045	16.3944557	6	67
Peru	lineage2	ISONIAZID	3.96902846	37.049243	36	70
Peru	lineage2	KANAMYCIN	21.7752016	26.8889065	4	67
Peru	lineage2	MOXIFLOXACIN	6.40783111	11.9277359	6	67
Peru	lineage2	PYRAZINAMIDE	1.27516939	66.3620373	18	66
Peru	lineage2	RIFABUTIN	2.54931656	59.6284281	15	67
Peru	lineage2	RIFAMPICIN	2.15441269	35.4384595	29	70
Peru	lineage2	STREPTOMYCIN	1.65717543	27.040606	30	71
Peru	lineage4	AMIKACIN	0.71878918	21.235315	62	834
Peru	lineage4	CAPREOMYCIN	0.45620301	20.9603883	90	836
Peru	lineage4	ETHAMBUTOL	0.24556105	61.8951336	366	848
Peru	lineage4	ETHIONAMIDE	0.20541336	36.3621585	128	837
Peru	lineage4	KANAMYCIN	0.77529421	21.8857849	80	838
Peru	lineage4	MOXIFLOXACIN	0.1352287	24.5999574	62	838
Peru	lineage4	PYRAZINAMIDE	0.16785888	37.9068111	249	836
Peru	lineage4	RIFABUTIN	0.22769151	63.8170599	290	836
Peru	lineage4	RIFAMPICIN	0.23406283	65.1097268	380	846
Peru	lineage4	STREPTOMYCIN	0.29405066	40.4717248	217	849

Romania	lineage4	AMIKACIN	1.39730865	37.5979497	6	24
Romania	lineage4	KANAMYCIN	1.22541928	37.8051561	9	24
Romania	lineage4	OFLOXACIN	1.3867589	18.1948415	8	24
Romania	lineage4	STREPTOMYCIN	2.25843497	37.0637158	18	24
Russia	lineage4	AMIKACIN	2.86589749	12.3950523	6	81
Russia	lineage4	CAPREOMYCIN	2.09346181	6.81421611	6	81
Russia	lineage4	ETHAMBUTOL	1.92899389	20.3561089	34	272
Russia	lineage4	ISONIAZID	1.76618006	22.6826158	107	272
Russia	lineage4	MOXIFLOXACIN	1.47224284	26.8682661	8	72
Russia	lineage4	OFLOXACIN	2.24412437	25.4136502	11	81
Russia	lineage4	PROTHIONAMIDE	1.99531033	32.3899523	24	77
Russia	lineage4	PYRAZINAMIDE	2.01748677	28.0784826	35	271
Russia	lineage4	RIFAMPICIN	1.80284765	22.9829059	71	272
Russia	lineage4	STREPTOMYCIN	1.43352364	27.2493466	92	272
Sierra Leone	lineage4	ETHAMBUTOL	3.58661725	38.3431992	7	54
Sierra Leone	lineage4	ISONIAZID	3.58460162	37.579056	14	54
Sierra Leone	lineage4	PYRAZINAMIDE	3.24393546	8.26437787	3	54
Sierra Leone	lineage4	RIFAMPICIN	3.11704905	39.4228803	7	54
Sierra Leone	lineage4	STREPTOMYCIN	4.30714626	41.8355374	25	54
Sierra Leone	lineage6	ETHAMBUTOL	7.22084469	7.22084469	1	13
Sierra Leone	lineage6	ISONIAZID	5.63814912	15.2731798	3	13
Sierra Leone	lineage6	PYRAZINAMIDE	6.48117814	6.48117814	1	13
Sierra Leone	lineage6	RIFAMPICIN	6.80992989	6.80992989	1	13
Sierra Leone	lineage6	STREPTOMYCIN	6.97020683	7.65250547	2	13
South Africa	lineage1	KANAMYCIN	1.85113964	2.42645491	2	11
South Africa	lineage1	OFLOXACIN	2.69081132	2.69081132	1	11
South Africa	lineage1	RIFAMPICIN	11.4706731	13.1399868	6	11
South Africa	lineage1	STREPTOMYCIN	11.966305	11.966305	5	11
South Africa	lineage2	AMIKACIN	1.24686812	8.1096853	40	185
South Africa	lineage2	CAPREOMYCIN	0.51613006	5.33061692	36	204
South Africa	lineage2	CYCLOSERINE	0.88658318	3.73536405	14	94
South Africa	lineage2	ETHAMBUTOL	0.63614699	4.82426007	45	228
South Africa	lineage2	ETHIONAMIDE	0.98541416	4.688532	27	117
South Africa	lineage2	ISONIAZID	0.62310473	7.63656863	62	235
South Africa	lineage2	KANAMYCIN	0.61266545	8.12053365	46	222
South Africa	lineage2	MOXIFLOXACIN	0.66851319	6.26083091	28	197
South Africa	lineage2	OFLOXACIN	0.50956701	6.50664166	41	228
South Africa	lineage2	PARA-AMINOSALICYLIC_ACID	1.04542312	1.1426862	2	94

South Africa	lineage2	PYRAZINAMIDE	1.29778195	7.82642287	40	188
South Africa	lineage2	RIFAMPICIN	0.71101793	8.17285666	60	228
South Africa	lineage2	STREPTOMYCIN	0.92064293	7.12905176	39	233
South Africa	lineage3	ETHAMBUTOL	2.14135238	2.14135238	1	25
South Africa	lineage3	ISONIAZID	3.59255781	8.72534418	6	27
South Africa	lineage3	OFLOXACIN	3.2230357	3.2230357	1	24
South Africa	lineage3	PYRAZINAMIDE	5.46801635	5.46801635	1	20
South Africa	lineage3	RIFAMPICIN	3.9388676	4.52702298	5	27
South Africa	lineage3	STREPTOMYCIN	8.12275203	8.12275203	3	23
South Africa	lineage4	AMIKACIN	1.31297781	8.31201078	12	312
South Africa	lineage4	CAPREOMYCIN	0.63214955	12.5108026	25	397
South Africa	lineage4	CIPROFLOXACIN	1.61924361	2.04148373	5	24
South Africa	lineage4	CYCLOSERINE	1.26383208	13.813988	3	17
South Africa	lineage4	ETHAMBUTOL	0.57709825	51.7329068	40	433
South Africa	lineage4	ETHIONAMIDE	0.84700929	27.4506369	23	99
South Africa	lineage4	ISONIAZID	0.68075679	55.0883957	110	463
South Africa	lineage4	KANAMYCIN	0.41297531	7.99280861	31	438
South Africa	lineage4	MOXIFLOXACIN	1.28047981	37.829983	15	332
South Africa	lineage4	NICOTINAMIDE	1.19555431	25.6138377	14	58
South Africa	lineage4	OFLOXACIN	0.74263956	17.5183596	37	433
South Africa	lineage4	PYRAZINAMIDE	1.36093204	37.3084331	34	328
South Africa	lineage4	RIFAMPICIN	0.81200268	54.5208129	95	447
South Africa	lineage4	STREPTOMYCIN	0.48549215	46.1827541	73	441
South Korea	lineage2	AMIKACIN	7.07477744	54.0189436	12	20
South Korea	lineage2	CYCLOSERINE	1.79203541	25.6562634	6	19
South Korea	lineage2	ETHIONAMIDE	1.14922024	52.171675	16	20
South Korea	lineage2	KANAMYCIN	8.30862501	52.9876676	13	20
South Korea	lineage2	MOXIFLOXACIN	2.40581152	29.5529413	7	13
South Korea	lineage2	RIFABUTIN	7.09475867	25.5445206	14	19
South Korea	lineage2	STREPTOMYCIN	11.6923909	52.4422229	5	18
South Korea	lineage3	CAPREOMYCIN	16.3177658	38.5632113	5	15
South Korea	lineage3	ETHIONAMIDE	18.6648822	18.6648822	1	15
United Kingdom	lineage1	ETHAMBUTOL	18.1985203	32.0307606	6	256
United Kingdom	lineage1	ISONIAZID	2.92439445	35.1407718	54	256
United Kingdom	lineage1	PYRAZINAMIDE	12.474108	24.3064771	10	255
United Kingdom	lineage1	RIFAMPICIN	14.3194018	30.9861484	10	257
United Kingdom	lineage1	STREPTOMYCIN	7.14701312	23.8543679	5	64

United Kingdom	lineage2	AMIKACIN	3.2986758	27.5402301	5	21
United Kingdom	lineage2	CAPREOMYCIN	3.73214962	26.5622266	5	21
United Kingdom	lineage2	CIPROFLOXACIN	3.92334653	26.3210851	2	21
United Kingdom	lineage2	ETHAMBUTOL	1.94240671	15.3771182	19	109
United Kingdom	lineage2	ISONIAZID	4.06772187	28.5736922	30	112
United Kingdom	lineage2	KANAMYCIN	3.38774353	25.5457027	7	21
United Kingdom	lineage2	MOXIFLOXACIN	4.83390928	25.2953986	4	21
United Kingdom	lineage2	OFLOXACIN	4.88603615	23.0684328	5	20
United Kingdom	lineage2	PYRAZINAMIDE	4.29427743	8.43998683	10	113
United Kingdom	lineage2	RIFAMPICIN	4.32592129	24.4565159	26	110
United Kingdom	lineage2	STREPTOMYCIN	6.56750011	22.668357	13	49
United Kingdom	lineage3	CIPROFLOXACIN	14.49252	18.3543198	4	86
United Kingdom	lineage3	ETHAMBUTOL	16.1645946	21.5108466	2	629
United Kingdom	lineage3	ISONIAZID	5.30637527	29.6236332	73	630
United Kingdom	lineage3	MOXIFLOXACIN	22.3408648	22.3408648	2	24
United Kingdom	lineage3	OFLOXACIN	15.9337336	22.9495038	3	24
United Kingdom	lineage3	PYRAZINAMIDE	5.93852757	23.1971077	7	629
United Kingdom	lineage3	RIFAMPICIN	5.51932189	25.4676869	19	630
United Kingdom	lineage3	STREPTOMYCIN	4.36332444	17.2928121	4	94
United Kingdom	lineage4	CAPREOMYCIN	15.3044832	15.3044832	1	26
United Kingdom	lineage4	CIPROFLOXACIN	5.11353832	20.1491402	8	126
United Kingdom	lineage4	ETHAMBUTOL	5.65250433	20.0110555	8	753
United Kingdom	lineage4	ISONIAZID	5.46289596	24.0248095	27	756
United Kingdom	lineage4	MOXIFLOXACIN	4.70010935	8.36192806	5	24
United Kingdom	lineage4	OFLOXACIN	5.63610139	8.34281432	4	24
United Kingdom	lineage4	PYRAZINAMIDE	3.45300439	15.2102373	9	750
United Kingdom	lineage4	RIFAMPICIN	2.36193259	19.0450145	19	753

United Kingdom	lineage4	STREPTOMYCIN	2.71912994	15.2138273	11	155
Uzbekistan	lineage4	AMIKACIN	0.95802284	20.5554703	5	26
Uzbekistan	lineage4	CAPREOMYCIN	1.12546009	22.0902573	6	26
Uzbekistan	lineage4	ETHAMBUTOL	1.01437782	18.3039446	17	37
Uzbekistan	lineage4	PYRAZINAMIDE	1.03596132	18.2158075	8	37

Supplementary Table 8 Transmission rate estimates and association with GDP by country. Transmission rate X/Y indicates X unique MRSCA dates/Y number of MRSCA dates for a specific country and drug. Legend: unique = number of unique MRSCA dates for drug and country, total = total number of dated resistant isolates for drug and country, Lower Bound (LB) = (total-unique)/total, Note: Data for country fewer than 10 resistant isolates per drug not shown.

	**Pooled**			**INH**			**RIF**			**PZA**		
**Country**	**unique**	**total**	**LB Transmission**	**unique**	**total**	**LB Transmission**	**unique**	**total**	**LB Transmission**	**unique**	**total**	**LB Transmission**
**China**	296	441	33%	56	101	45%	56	101	45%			
**Germany**	90	113	20%	30	41	27%						
**India**	13	20	35%									
**Malawi**	44	68	35%	23	37	38%						
**Mali**	18	30	40%	5	12	58%						
**Moldova**	8	13	38%									
**Netherlands**												
**Peru**	1260	2116	40%	12	36	67%	382	715	47%	177	268	34%
**Romania**	29	41	29%									
**Russia**	302	394	23%	73	107	32%	56	71	21%	32	35	9%
**Sierra Leone**	48	64	25%	14	17	18%						
**South Africa**	491	1015	52%	69	178	61%	72	167	57%	34	75	55%
**South Korea**	43	79	46%				5	14	64%			
**United Kingdom**	299	416	28%	133	184	28%	49	74	34%	27	36	25%
**Uzbekistan**	31	36	14%									
**Total**	2972	4846	39%	415	713	42%	620	1142	46%	270	414	35%

	FLQ			STR			SLIs			EMB		
**Country**	**unique**	**total**	**LB Transmission**	**unique**	**total**	**LB Transmission**	**unique**	**total**	**LB Transmission**	**unique**	**total**	**LB Transmission**
**China**	32	44	27%	48	71	32%	33	36	8%	52	58	10%
**Germany**				40	50	20%						
**India**												
**Malawi**				15	23	35%						
**Mali**												
**Moldova**												
**Netherlands**												
**Peru**	59	68	13%	170	247	31%	194	251	23%	239	395	39%
**Romania**				9	18	50%	13	15	13%			
**Russia**	19	19	0%	69	92	25%	10	12	17%	31	34	9%
**Sierra Leone**				16	27	41%						
**South Africa**	79	128	38%	65	120	46%	86	192	55%	54	86	37%
**South Korea**							14	30	53%			
**United Kingdom**	27	37	27%	28	33	15%	14	17	18%	21	35	40%
**Uzbekistan**							9	11	18%	14	17	18%
**Total**	216	296	27%	460	681	32%	373	564	34%	411	625	34%

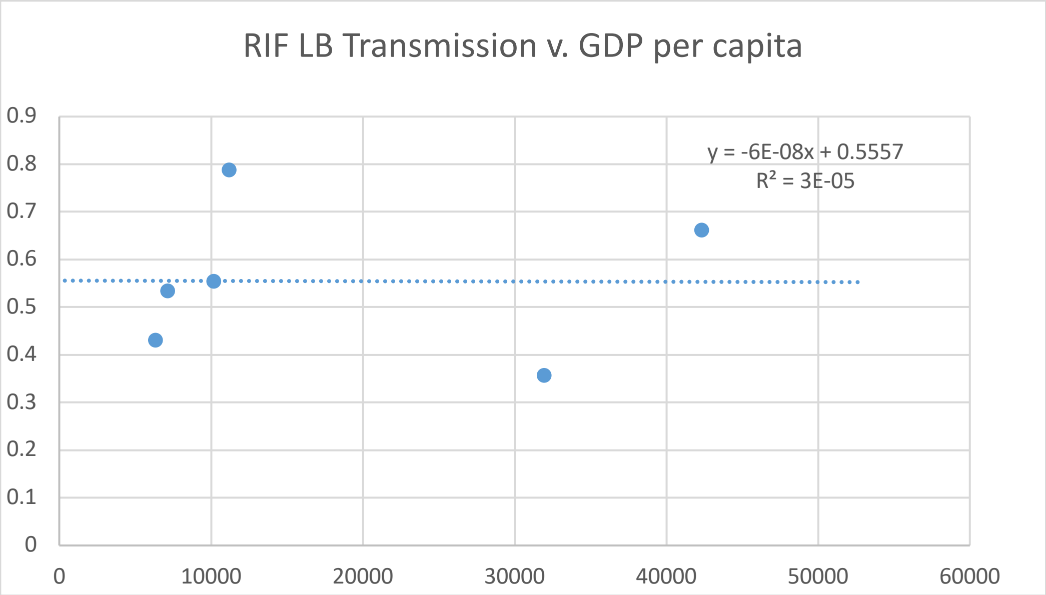
RIF LB Transmission v. GDP per capita
y = -6E-08x + 0.5557
R² = 3E-05
0.9
0.8
0.7
0.6
0.5
0.4
0.3
0.2
0.1
0
0
10000
20000
30000
40000
50000
60000

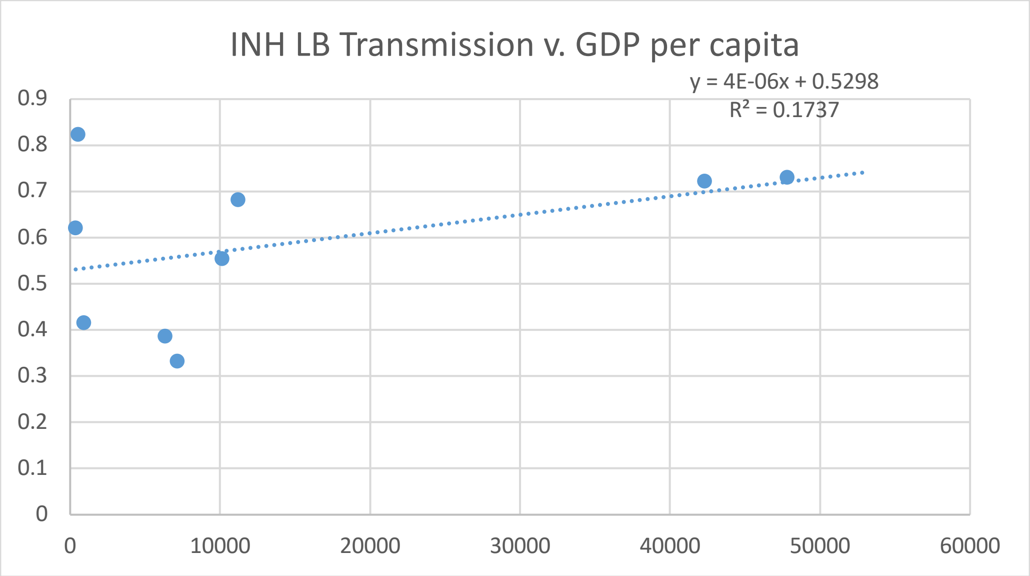
INH LB Transmission v. GDP per capita
y = 4E-06x + 0.5298
R² = 0.1737
0.9
0.8
0.7
0.6
0.5
0.4
0.3
0.2
0.1
0
0
10000
20000
30000
40000
50000
60000

Supplementary Table 9 Country, lineage, median MRSCA, and number of isolates for groups that provide evidence of RIF and ETH ordering (green highlighting indicates groups that provide evidence of RIF ordered before ETH)

country	lineage	RIF MRSCA (number of isolates)	ETH MRSCA (number of isolates)
China	2	15.5 (IQR 12.5 – 18.8, n=81)	10.4 (IQR 8.6 – 13.5, n=28)
China	4	19.4 (IQR 17.1 – 20.7, n=20)	15.3 (IQR 14.1 – 16.4, n=2)
Peru	2	12.9 (IQR 4.7 – 31.4, n=45)	16 (IQR 11.6 – 53.6, n=8)
South Africa	2	3.86 (IQR 2.4 – 5.2, n=60)	1.86 (IQR 1.3 – 2.6, n=27)
Russia	4	5.7 (IQR 3.9 – 10.2, n=71)	9.1 (IQR 6.4 – 14.5, n=24)
Peru	4	6 (IQR 2.3 – 15.4, n=670)	2.5 (IQR 1.5 – 7.8, n=128)
South Korea	2	17.7 (IQR 15.8 – 17.7, n=14)	15.5 (IQR 2.3 – 25.5, n=16)
Germany	2	16 (IQR 9.9 – 17.4, n=7)	16 (IQR 12.7 – 19.3, n=2)
South Africa	4	5 (IQR 2.0 – 7.8, n=96)	1.3 (IQR 1.0 – 9.7, n=23)

Supplementary Table 10 MRSCA age is not associated with the earliest date of drug introduction into clinical use.

Drug	Median MRSCA	Date Introduced Years ago
**INH**	11.4 (IQR 6.3 – 16.2)	67
**RIF**	7.61 (IQR 3.0 – 16.0)	54
**PZA**	3.56 (IQR 2.0 – 8.8)	67
**FLQ**	4.81 (IQR 1.8 – 10.5)	34
**STR**	7.67 (IQR 3.4 – 13.0)	75
**SLIs**	4.02 (IQR 1.9 – 8.3)	61
**EMB**	5.01 (IQR 2.1 – 12.5)	54
**ETH**	4.01 (IQR 1.6 – 12.2)	59
**CYS**	2.05 (IQR 1.5 – 9.0)	64

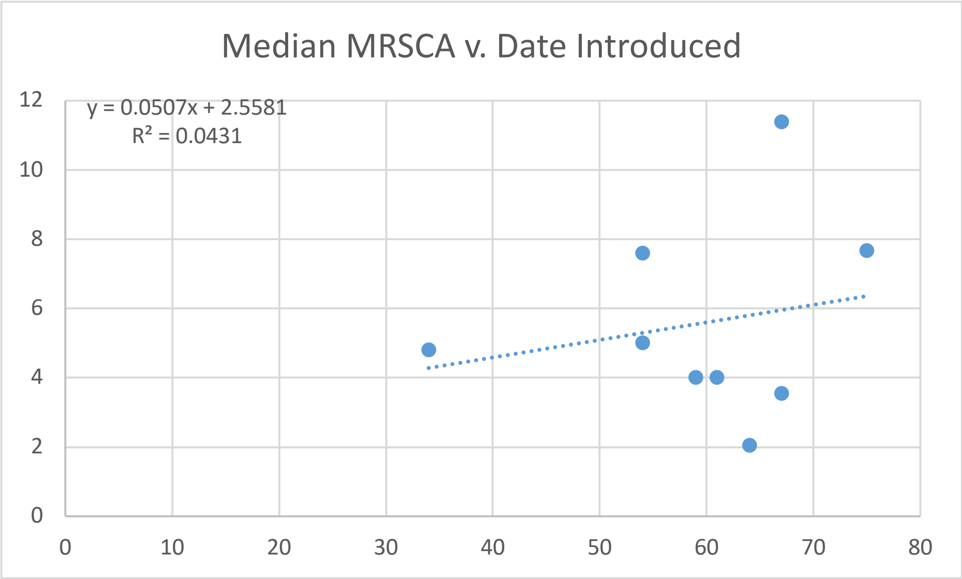

Supplementary Table 11 Dates Drugs Introduced

Drug	Year Introduced
Isoniazid(28)	1952
Rifampicin(28)	1965
Pyrazinamide(29)	1952
Streptomycin(28)	1944
Ethambutol(28)	1965
Ethionamide(28)	1960
Kanamycin/amikacin(28)	1958
Cycloserine(28)	1955
Capreomycin(28)	1967
PAS(28)	1944
Ofloxacin(30)	1985
Moxifloxacin(30)	1999
Ciprofloxacin(30)	1986

Supplementary Table 12 Pairwise Country MRSCA date comparison

Dark red indicates P-value <0.001 (Bonferroni threshold); pink indicates P-value <0.01; white indicates P-value ≥0.01. Number of datapoints in each comparison indicated in parenthesis. *No PZA resistance phenotypes was available on Chinese isolates.

**INH**	Peru	South Africa	China	UK	Russia
Peru		6 E-9 (214)	0.1 (137)	0.2 (220)	9 E-5 (143)
South Africa			7 E-29 (279)	2 E-32 (362)	3 E-7 (285)
China				0.2 (285)	4 E-17 (208)
UK					5 E-15 (291)
Russia					

**FLQ**	Peru	South Africa	China	UK	Russia
Peru		0.3 (196)	6 E-10 (112)	1 E-6 (105)	1 E-4 (87)
South Africa			2 E-19 (172)	1 E-15 (165)	2 E-7 (147)
China				0.04 (81)	0.6 (63)
UK					0.7 (56)
Russia					

**SLIs**	Peru	South Africa	China	UK	Russia
Peru		0.5 (443)	5 E-8 (287)	5 E-5 (269)	0.2 (263)
South Africa			5 E-12 (228)	9 E-8 (210)	0.5 (204)
China				0.8 (54)	2 E-4 (48)
UK					1 E-3 (30)
Russia					

**RIF**	Peru	South Africa	China	UK	Russia
Peru		4 E-4 (882)	1 E-14 (816)	2 E-8 (789)	0.9 (786)
South Africa			6 E-31 (268)	1 E-20 (241)	1 E-3 (238)
China				0.02 (175)	3 E-17 (172)
UK					3 E-11 (145)
Russia					

**PZA***	Peru	South Africa	China	UK	Russia
Peru		0.01 (343)		1 E-8 (304)	1 E-4 (303)
South Africa				2 E-11 (111)	4 E-3 (110)
China					
UK					3 E-4 (71)
Russia					

**EMB**	Peru	South Africa	China	UK	Russia
Peru		2 E-9 (481)	1 E-7 (453)	7 E-6 (430)	0.9 (429)
South Africa			2 E-17 (144)	1 E-12 (121)	1 E-6 (120)
China				0.4 (93)	3 E-7 (92)
UK					3 E-6 (69)
Russia					

Supplementary Table 13 GDP Per Capita Verses Median MRSCA date for INH, RIF, SLIs, and FLQ.

Countries	GDP per capita	Median MRSCA Date INH (Years Ago)	Median MRSCA Date RIF (Years Ago)	Median MRSCA Date SLIs (Years Ago)	Median MRSCA Date FLQ (Years Ago)
**China**	10150	16.2 (IQR 13.0 – 19.3)	16.1 (IQR 12.7-19.3)	9.9 (IQR 7.8 – 13.6)	12.8 (IQR 10.4 – 15.8)
**Peru**	7140	19.0 (IQR 7.8 – 25.8)	6.5 (IQR 2.4 – 16.0)	2.6 (IQR 1.6 – 7.6)	2.4 (IQR 1.2 – 8.2)
**Russia**	11190	9.3 (IQR 6.1 – 12.2)	5.7 (IQR 3.9 – 10.2)	3.0 (IQR 2.7 – 6.5)	12.2 (IQR 5.1 - 21.3)
**South Africa**	6330	5.6 (IQR 3.9 – 8.7)	4.6 (IQR 2.5 – 6.8)	4.0 (IQR 1.9 – 5.6)	2.1 (IQR 1.4 – 3.3)
**United Kingdom**	42310	14.8 (IQR 11.4 – 19.5)	13.0 (IQR 10.7 – 19.0)	10.6 (IQR 7.3 – 11.2)	8.4 (IQR 6.7 – 18.4)
**Uzbekistan**	1480			2.5 (IQR 1.1 – 14.0)	
**Romania**	12510			11.7 (IQR 3.1 – 18.3)	10.2 (IQR 3.1 – 13.9)
**Mali**	933.88	5.0 (IQR 5.0 – 10.5)	3.4 (IQR 3.2 – 3.4)		
**Netherlands**	53020	11.6 (IQR 6.2 – 13.7)			
**Moldova**	3400		3.8 (IQR 3.1 – 4.2)		
**Malawi**	366.53	9.7 (IQR 7.9 – 13.4)	6.6 (IQR 5.5 – 6.6)		
**South Korea**	31940		17.7 (IQR 15.8 – 17.7)	16.1 (IQR 16.0 – 19.7)	12.0 (IQR 8.9 – 21.0)
**Sierra Leone**	516.76	6.9 (IQR 5.3 – 14.6)	5.2 (IQR 3.6 – 11.3)		
**Germany**	47790	12.5 (IQR 6.1 – 16.1)	13.9 (IQR 8.5 – 17.4)		
**India**	2200		11.3 (IQR 11.2 – 13.8)		

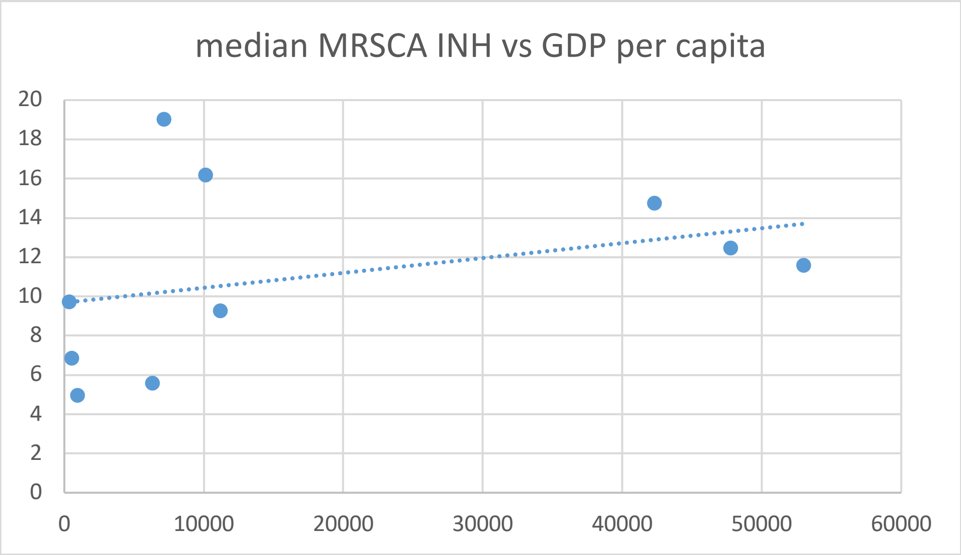

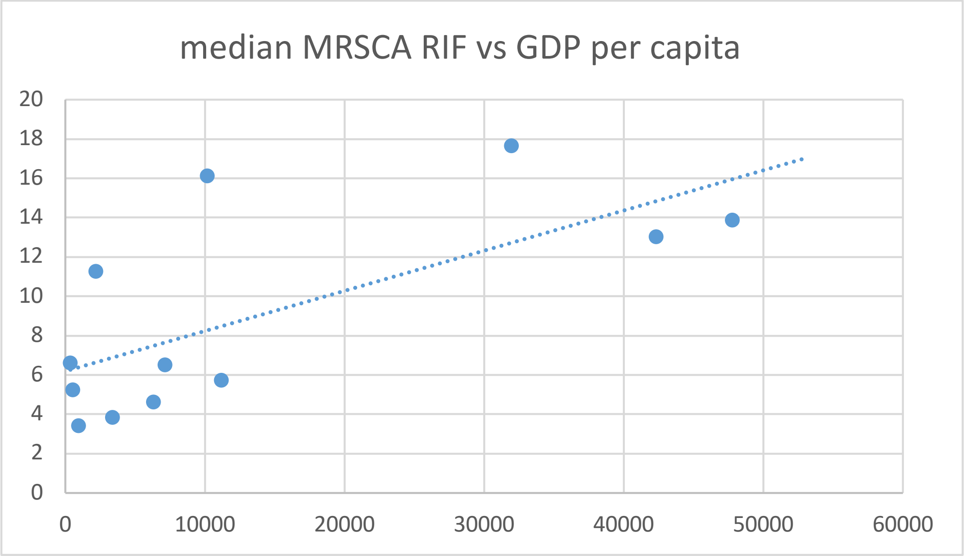
median MRSCA RIF vs GDP per capita
20
18
16
14
12
10
8
6
4
2
0
0
10000
20000
30000
40000
50000
60000

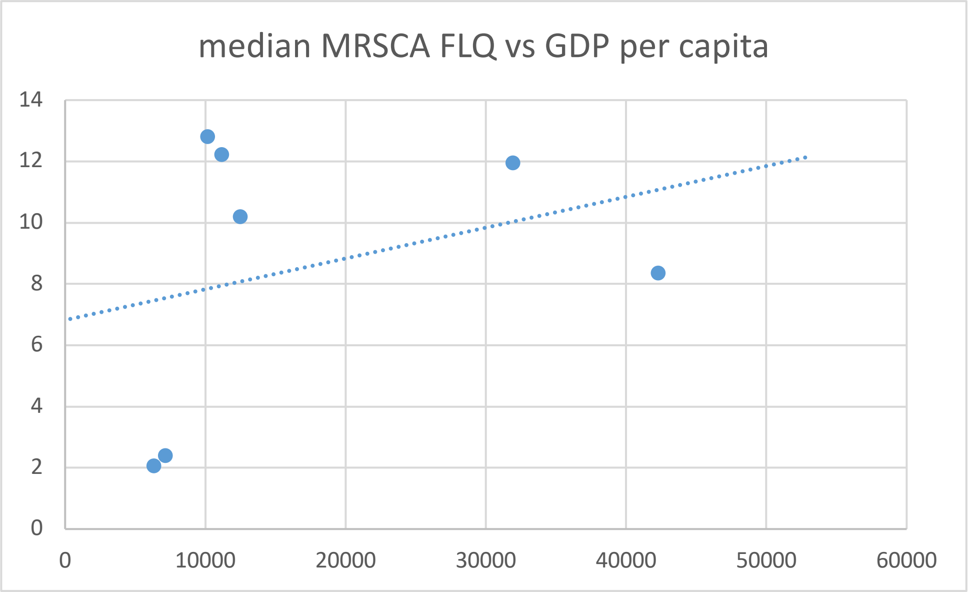
median MRSCA FLQ vs GDP per capita
14
12
10
8
6
4
2
0
0
10000
20000
30000
40000
50000
60000

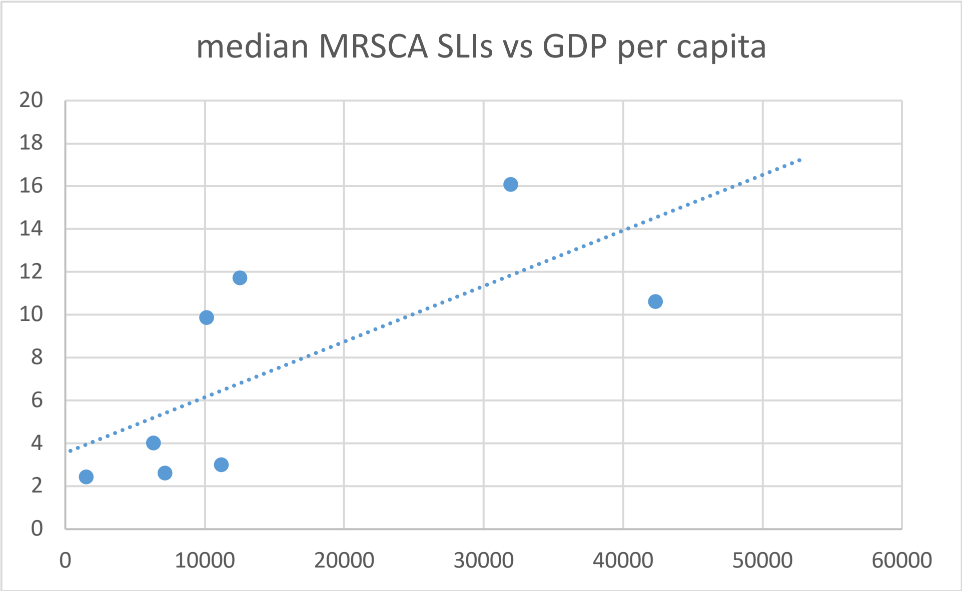

median MRSCA SLIs vs GDP per capita
20
18
16
14
12
10
8
6
4
2
0
0
10000
20000
30000
40000
50000
60000

Supplementary Table 14 Geographic variance of resistance mutations

A: *katG S315T* mutation prevalence among INH resistant isolates in Russia verses Peru (Fisher P-value $1*10^{-12}$)

	**Russia**	**Peru**
**Mutation**	444	510
**No Mutation**	82	250

B: *fabG/inhA*-15 C>T mutation prevalence among INH resistant isolates in Russia verses Peru (Fisher P-value $7*10^{-9}$)

	**Russia**	**Peru**
**Mutation**	43	149
**No Mutation**	483	611

C: 25 mutations that varied geographically to a larger extent than the mutation *fabG/inhA* promoter -15C>T (SD 10%, Range 0%-39%) , their standard deviations, and ranges.

**Mutation**	**Standard Deviation**	**Range of observed frequency across countries**
INH_SNP_CN_2155168_CG_katG_S315T	12.49%	54.55%-93.75%
INH_SNP_CN_4247429_AG_embB_M306V	12.03%	0.0%-43.56%
INH_SNP_CN_4247431_GA_embB_M306I	11.88%	0.0%-42.63%
INH_SNP_CN_4247431_GC_embB_M306I	11.88%	0.0%-42.63%
PZA_SNP_CN_2289073_GC_pncA_H57D	15.07%	0.0%-46.67%
EMB_SNP_CN_4247429_AG_embB_M306V	12.48%	8.33%-53.37%
EMB_SNP_CN_4247431_GA_embB_M306I	11.95%	9.02%-49.58%
EMB_SNP_CN_4247431_GC_embB_M306I	11.95%	9.02%-49.58%

EMB_SNP_CN_4247431_GT_embB_M306I	11.95%	9.02%-49.58%
STR_SNP_CN_781687_AG_rpsL_K43R	21.03%	0.0%-81.85%
STR_SNP_N_1472359_A514C_rrs	12.00%	0.0%-50.4%
STR_SNP_N_1473246_A1401G_rrs	17.90%	0.0%-60.0%
STR_SNP_CN_4407927_TG_gid_E92D	31.17%	0.0%-87.95%
STR_SNP_N_1472362_C517T_rrs	11.32%	0.0%-38.89%
STR_SNP_CN_4407967_AG_gid_L79S	20.81%	0.0%-72.73%
ETH_SNP_P_1673425_CT.15_fabG1.inhA	14.48%	27.78%-62.5%
ETH_SNP_CN_4326333_CG_ethA_A381P	19.24%	0.0%-48.44%
ETH_SNP_CN_1674481_TG_inhA_S94A	11.89%	0.0%-31.46%
KAN_SNP_N_1473246_A1401G_rrs	14.83%	45.65%-85.71%
CAP_SNP_N_1473246_A1401G_rrs	19.62%	23.08%-85.84%
AMK_SNP_N_1473246_A1401G_rrs	20.11%	46.43%-100.0%
AMK_SNP_N_1472359_A514C_rrs	20.90%	0.0%-64.0%
CIP_SNP_CN_7582_AG_gyrA_D94G	26.57%	16.67%-78.26%
OFLX_SNP_CN_7582_AG_gyrA_D94G	12.60%	8.33%-50.0%
OFLX_SNP_CN_7570_CT_gyrA_A90V	11.83%	5.45%-46.15%

D: Six mutations that varied by lineage to a larger extent than the mutation *fabG/inhA* promoter -15C>T (SD 9.3%, Range 8.8%-33%), their standard deviations, and ranges.

Mutation	Standard Deviation	Range of observed frequency across lineages
INH_SNP_CN_2155168_CG_katG_S315T	16.00%	40.28%-84.23%
INH_SNP_CN_2518919_GA_kasA_G269S	11.11%	0.0%-25.66%
PZA_SNP_CN_2288952_CT_pncA_G97D	11.74%	0.0%-27.27%
EMB_SNP_CN_4247429_AG_embB_M306V	13.65%	18.87%-53.85%
STR_SNP_CN_781687_AG_rpsL_K43R	20.23%	4.76%-57.36%
STR_SNP_CN_4407927_TG_gid_E92D	43.23%	0.0%-99.83%

Supplementary Table 15 Sensitivity and Specificity of commercial tests, RF-select, and DA-select for INH, RIF, SLIS, and FLQ in five countries with the largest number of phenotyped strained: Russia, South Africa, Peru, Uzbekistan, and United Kingdom

Legend: Sensitivity (Percent of resistant isolates classified as resistant), Specificity (Percent of susceptible isolates classified as susceptible)

Drug	commercial test-Peru		commercial test-Russia		commercial test-South Africa		commercial test-Uzbekistan		commercial test-UnitedKingdom	
	Sensitivity	Specificity	Sensitivity	Specificity	Sensitivity	Specificity	Sensitivity	Specificity	Sensitivity	Specificity
INH	84% (641/760)	90% (151/168)	87% (459/526)	91% (281/310)	88% (168/190)	72% (444/619)	90% (238/264)	NA 0/0	87% (245/283)	99% (1559/1567)
RIF	90% (623/692)	89% (209/236)	82% (347/425)	88% (363/412)	95% (158/166)	70% (433/616)	95% (248/262)	50% (1/2)	91% (86/95)	99% (1742/1750)
FLQ	38% (46/121)	98% (788/801)	38% (51/133)	94% (275/292)	77% (86/111)	87% (510/585)	0% (0/7)	100% (213/213)	90% (26/29)	98% (295/301)
SLI	47% (101/214)	98%(692/708)	77% (63/82)	48%(163/343)	77% (101/131)	91%(486/536)	79% (44/56)	66% (109/164)	90% (9/10)	100%(83/83)

Drug	RF-select WGS test - Peru		RF-select WGS test-Russia		RF-select WGS test -South Africa		RF-select WGS test-Uzbekistan		RF-select WGS test-UnitedKingdom	
	Sensitivity	Specificity	Sensitivity	Specificity	Sensitivity	Specificity	Sensitivity	Specificity	Sensitivity	Specificity
INH	91% (692/760)	86% (144/168)	88% (463/526)	91% (281/310)	90% (171/190)	71% (438/619)	94% (248/264)	NA 0/0	90% (255/283)	98% (1537/1567)
RIF	91% (629/692)	89% (210/236)	82% (350/425)	88% (363/412)	96% (159/166)	70% (432/616)	96% (252/262)	50% (1/2)	100% (95/95)	98% (1722/1750)
FLQ	35% (42/121)	99% (795/801)	36% (48/133)	95% (276/292)	78% (87/111)	88% (512/585)	0% (0/7)	100% (213/213)	90% (26/29)	98% (296/301)
SLI	50% (105/214)	97%(686/708)	77% (63/82)	45%(156/343)	78% (102/131)	90%(482/536)	77% (43/56)	65% (106/164)	90% (9/10)	94%(78/83)

Drug	DA-select WGS test - Peru		DA-select WGS test-Russia		DA-select WGS test -South Africa		DA-select WGS test-Uzbekistan		DA-select WGS test-UnitedKingdom	
	Sensitivity	Specificity	Sensitivity	Specificity	Sensitivity	Specificity	Sensitivity	Specificity	Sensitivity	Specificity
INH	92% (698/760)	88% (147/168)	88% (463/526)	90% (280/310)	92% (175/190)	71% (438/619)	98% (258/264)	NA 0/0	94% (267/283)	98% (1537/1567)
RIF	91% (633/692)	89% (210/236)	82% (350/425)	88% (363/412)	96% (159/166)	70% (431/616)	97% (253/262)	50% (1/2)	100% (95/95)	98% (1722/1750)
FLQ	33% (40/121)	99% (796/801)	36% (48/133)	95% (277/292)	78% (87/111)	88% (513/585)	0% (0/7)	100% (213/213)	97% (28/29)	98% (296/301)
SLI	46% (98/214)	96%(681/708)	70% (57/82)	51%(174/343)	79% (103/131)	90%(482/536)	73% (41/56)	85% (140/164)	80% (8/10)	90%(75/83)

Supplementary Table 16 Resistance Mutation Counts Per Country and Per Isolate Phenotype

Amikacin (AMK) Data, Abbreviations: counts = how many isolates had this mutation, no_data = how many isolates had this mutation but no data on whether they were phenotypically resistant or not to AMK, susceptible = how many isolates had this mutation but were phenotypically susceptible to AMK, resistant = how many isolates had this mutation but were resistant to AMK

country	AMK_SNP_N_1472359_A514C_rrs	AMK_SNP_N_1473246_A1401G_rrs	AMK_othersnp	AMK_rrs_A906G_u	AMK_rrs_C517T_u	AMK_rrs_G1484T_u	AMK_unknown
Azerbaijan	0	0	0	0	0	0	0
Bangladesh	0	0	0	0	0	0	0
Belarus	20	51	0	0	0	0	0
Brazil	0	0	0	0	0	0	0
Burma	0	0	0	0	0	0	0
Canada	0	0	0	0	0	0	0
China	10	14	0	0	0	0	0
Colombia	0	1	0	0	0	0	0
Democratic Republic of the Congo	0	0	0	0	0	0	0
Denmark	0	0	0	0	0	0	0
Djibouti	0	0	0	0	0	0	0
Dominican Republic	0	0	0	0	0	0	0
Estonia	0	0	0	0	0	0	0
Georgia	0	3	0	0	0	0	0
Germany	3	3	0	0	0	0	1
Guinea	0	0	0	0	0	0	0
India	0	0	0	0	0	0	0
Indonesia	0	0	0	0	0	0	0
Iran	1	3	0	0	0	0	0
Kazakhstan	0	0	0	0	0	0	0
Malawi	0	0	0	0	0	0	0
Mali	1	0	0	0	0	0	0
Moldova	0	0	0	0	0	0	0
Morocco	0	0	0	0	0	0	0
Nepal	1	0	0	0	0	0	0
Netherlands	1	0	0	0	0	0	0
Nigeria	0	0	0	0	0	0	0
Not Provided	39	160	5	0	8	4	100
Pakistan	0	0	0	0	0	0	0
Peru	14	110	6	3	0	0	48
Philippines	0	0	0	0	0	0	0
Portugal	0	0	0	0	0	0	0
Romania	2	12	0	0	0	0	0
Russia	17	35	0	0	14	1	15
Rwanda	0	2	0	0	0	0	0
Sierra Leone	0	0	0	0	0	0	0
South Africa	83	164	0	0	0	0	11
South Korea	2	26	0	1	0	0	23
Spain	0	0	0	0	0	0	0
Swaziland	0	0	0	0	0	0	0
Thailand	0	0	0	0	0	0	0
Turkmenistan	0	0	0	0	0	0	0
Uganda	0	1	0	0	0	0	0
United Kingdom	16	8	0	0	0	0	0
Uzbekistan	13	46	1	0	0	0	15
Vietnam	0	0	0	0	0	0	0

resistance mutation	counts	no_data	susceptible	resistant
AMK_SNP_N_1473246_A1401G_rrs	639	208	45	386
AMK_SNP_N_1472359_A514C_rrs	223	84	38	101
AMK_unknown	213	0	0	213
AMK_rrs_C517T_u	22	0	0	22
AMK_rrs_G1484T_u	5	0	0	5
AMK_rrs_A906G_u	4	0	0	4
AMK_rrs_C513T_u	4	0	0	4
AMK_rrs_A1461G_u	1	0	0	1
AMK_rrs_A908C_u	1	0	0	1
AMK_rrs_C1105G_u	1	0	0	1
AMK_rrs_C1402T_u	1	0	0	1
AMK_rrs_C799A_u	1	0	0	1
AMK_rrs_C905A_u	1	0	0	1
AMK_rrs_C905G_u	1	0	0	1
AMK_rrs_G878A_u	1	0	0	1

Capreomycin (CAP) Data, Abbreviations: counts = how many isolates had this mutation, no_data = how many isolates had this mutation but no data on whether they were phenotypically resistant or not to CAP, susceptible = how many isolates had this mutation but were phenotypically susceptible to CAP, resistant = how many isolates had this mutation but were resistant to CAP

country	CAP_SNP_N_1472753_A908C_rrs	CAP_SNP_N_1473246_A1401G_rrs	CAP_othersnp	CAP_rrs_A514C_u	CAP_rrs_C517T_u	CAP_rrs_G1484T_u	CAP_unknown
Azerbaijan	0	0	0	0	0	0	0
Bangladesh	0	0	0	0	0	0	0
Belarus	0	51	0	0	0	0	0
Brazil	0	0	0	0	0	0	0
Burma	0	0	0	0	0	0	0
Canada	0	0	0	0	0	0	0
China	0	14	0	1	1	0	9
Colombia	0	1	0	0	0	0	0
Democratic Republic of the Congo	0	0	0	0	0	0	0
Denmark	0	0	0	0	0	0	0
Djibouti	0	0	0	0	0	0	0
Dominican Republic	0	0	0	0	0	0	0
Estonia	0	0	0	0	0	0	0
Georgia	0	3	0	0	0	0	0
Germany	0	3	0	0	0	0	1
Guinea	0	0	0	0	0	0	0
India	0	0	0	0	0	0	0
Indonesia	0	0	0	0	0	0	0
Iran	0	3	0	0	0	0	0
Kazakhstan	0	0	0	0	0	0	0
Malawi	0	0	0	0	0	0	0
Mali	0	0	0	0	0	0	0
Moldova	0	0	0	0	0	0	0
Morocco	0	0	0	0	0	0	0
Nepal	0	0	0	0	0	0	0
Netherlands	0	0	0	0	0	0	0
Nigeria	0	0	0	0	0	0	0
Not Provided	2	160	7	1	0	7	53
Pakistan	0	0	0	0	0	0	0
Peru	3	110	13	4	0	0	87
Philippines	0	0	0	0	0	0	0
Portugal	0	0	0	0	0	0	0
Romania	1	12	0	0	0	0	0
Russia	1	35	0	0	21	1	20
Rwanda	0	2	0	0	0	0	0
Sierra Leone	0	0	0	0	0	0	0
South Africa	0	164	0	1	0	0	15
South Korea	0	26	1	0	0	0	9
Spain	0	0	0	0	0	0	0
Swaziland	0	0	0	0	0	0	0
Thailand	0	0	0	0	0	0	0
Turkmenistan	0	0	0	0	0	0	0
Uganda	4	1	0	0	0	0	0
United Kingdom	0	8	0	0	0	0	1
Uzbekistan	0	46	2	1	0	0	13
Vietnam	0	0	0	0	0	0	0

resistance mutation	counts	no_data	susceptible	resistant
CAP_SNP_N_1473246_A1401G_rrs	639	167	127	345
CAP_unknown	208	0	0	208
CAP_rrs_C517T_u	22	0	0	22
CAP_SNP_N_1472753_A908C_rrs	11	4	7	0
CAP_rrs_A514C_u	8	0	0	8
CAP_rrs_G1484T_u	8	0	0	8
CAP_rrs_A906G_u	5	0	0	5
CAP_rrs_C513T_u	5	0	0	5
CAP_rrs_C1402T_u	4	0	0	4
CAP_rrs_C1105G_u	1	0	0	1
CAP_rrs_C1390T_u	1	0	0	1
CAP_rrs_C1402A_u	1	0	0	1
CAP_rrs_C239A_u	1	0	0	1
CAP_rrs_C905A_u	1	0	0	1
CAP_rrs_T1322G_u	1	0	0	1
CAP_rrs_T16A_u	1	0	0	1
CAP_rrs_T16C_u	1	0	0	1
CAP_rrs_T556C_u	1	0	0	1
CAP_SNP_N_1473109_T1264G_rrs	0	0	0	0
CAP_SNP_N_1473160_G1315A_rrs	0	0	0	0
CAP_SNP_N_1473343_G1498T_rrs	0	0	0	0

Ciprofloxacin (CIP) Data, Abbreviations: counts = how many isolates had this mutation, no_data = how many isolates had this mutation but no data on whether they were phenotypically resistant or not to CIP, susceptible = how many isolates had this mutation but were phenotypically susceptible to CIP, resistant = how many isolates had this mutation but were resistant to CIP

country	CIP_SNP_CN_7570_CT_gyrA_A90V	CIP_SNP_CN_7572_TC_gyrA_S91P	CIP_SNP_CN_7581_GT_gyrA_D94Y	CIP_SNP_CN_7582_AC_gyrA_D94A	CIP_SNP_CN_7582_AG_gyrA_D94G	CIP_othersnp	CIP_unknown
Azerbaijan	0	0	0	0	0	0	0
Bangladesh	0	0	0	0	0	0	0
Belarus	23	6	3	11	21	2	0
Brazil	0	0	0	0	0	0	0
Burma	0	0	0	1	0	0	0
Canada	0	0	0	0	0	0	0
China	11	4	4	7	18	0	0
Colombia	0	0	0	0	0	0	0
Democratic Republic of the Congo	0	0	0	0	0	0	0
Denmark	0	0	0	0	0	0	0
Djibouti	0	0	0	0	0	0	0
Dominican Republic	0	0	0	0	0	0	0
Estonia	0	0	0	0	1	0	0
Georgia	0	0	0	0	0	0	0
Germany	0	2	0	1	2	0	0
Guinea	0	0	0	0	0	0	0
India	2	0	1	0	2	0	0
Indonesia	0	0	0	0	0	0	0
Iran	3	0	0	1	6	0	0
Kazakhstan	0	0	0	0	0	0	0
Malawi	0	0	0	0	0	0	0
Mali	1	0	0	0	0	0	0
Moldova	0	0	0	0	0	1	0
Morocco	0	0	0	0	0	0	0
Nepal	0	0	0	0	0	0	0
Netherlands	0	0	0	0	1	0	0
Nigeria	0	0	0	0	0	0	0
Not Provided	57	36	13	25	86	11	6
Pakistan	1	0	0	0	0	0	0
Peru	20	1	2	9	29	7	0
Philippines	0	0	0	0	0	0	0
Portugal	0	0	0	0	0	0	0
Romania	2	0	1	3	1	0	0
Russia	10	5	3	12	31	0	0
Rwanda	1	0	0	0	0	0	0
Sierra Leone	0	0	0	0	0	0	0
South Africa	78	4	6	21	44	1	0
South Korea	13	3	1	2	14	4	22
Spain	0	0	0	0	0	0	0
Swaziland	0	0	0	0	0	0	0
Thailand	0	0	0	0	0	0	0
Turkmenistan	0	0	0	0	0	0	0
Uganda	0	1	0	0	0	0	0
United Kingdom	7	7	0	1	30	2	0
Uzbekistan	0	0	0	0	0	0	0
Vietnam	0	0	0	0	0	0	0

resistance mutation	counts	no_data	susceptible	resistant
CIP_SNP_CN_7582_AG_gyrA_D94G	286	249	1	36
CIP_SNP_CN_7570_CT_gyrA_A90V	229	201	12	16
CIP_SNP_CN_7582_AC_gyrA_D94A	94	92	2	0
CIP_SNP_CN_7572_TC_gyrA_S91P	69	63	2	4
CIP_SNP_CN_7581_GT_gyrA_D94Y	34	28	0	6
CIP_unknown	28	0	0	28
CIP_SNP_CN_6735_AC_gyrB_N538T	10	10	0	0
CIP_SNP_CN_7566_GA_gyrA_D89N	6	6	0	0
CIP_gyrA_A74S_u	2	0	0	2
CIP_gyrA_D94N_u	2	0	0	2
CIP_gyrB_A471V_u	2	0	0	2
CIP_gyrB_S486F_u	2	0	0	2
CIP_gyrB_T539N_u	2	0	0	2
CIP_gyrA_A288D_u	1	0	0	1
CIP_gyrA_D94H_u	1	0	0	1

Ethambutol (EMB) Data, Abbreviations: counts = how many isolates had this mutation, no_data = how many isolates had this mutation but no data on whether they were phenotypically resistant or not to EMB, susceptible = how many isolates had this mutation but were phenotypically susceptible to EMB, resistant = how many isolates had this mutation but were resistant to EMB

country	EMB_SNP_CN_4242182_GT_embC_A774S	EMB_SNP_CN_4247429_AG_embB_M306V	EMB_SNP_CN_4247431_GA_embB_M306I	EMB_SNP_CN_4247431_GC_embB_M306I	EMB_SNP_CN_4247431_GT_embB_M306I	EMB_othersnp	EMB_unknown
Azerbaijan	0	0	0	0	0	1	0
Bangladesh	0	0	0	0	0	1	2
Belarus	0	16	46	46	46	30	0
Brazil	0	0	0	0	0	0	0
Burma	0	0	1	1	1	1	0
Canada	0	0	0	0	0	0	0
China	0	28	17	17	17	45	5
Colombia	0	0	0	0	0	0	0
Democratic Republic of the Congo	0	0	0	0	0	0	0
Denmark	0	0	0	0	0	0	0
Djibouti	0	0	0	0	0	0	0
Dominican Republic	0	0	0	0	0	0	0
Estonia	0	2	0	0	0	0	0
Georgia	0	0	0	0	0	4	0
Germany	3	4	4	4	4	12	1
Guinea	0	1	0	0	0	0	0
India	0	4	0	0	0	4	0
Indonesia	0	0	0	0	0	0	0
Iran	0	4	2	2	2	6	1
Kazakhstan	0	2	0	0	0	0	0
Malawi	2	1	0	0	0	5	0
Mali	0	8	4	4	4	3	0
Moldova	0	5	5	5	5	11	0
Morocco	0	0	0	0	0	1	0
Nepal	0	0	0	0	0	0	1
Netherlands	0	0	0	0	0	0	0
Nigeria	0	0	1	1	1	0	0
Not Provided	54	203	135	135	135	280	34
Pakistan	0	1	0	0	0	0	0
Peru	220	139	178	178	178	367	121
Philippines	0	0	0	0	0	0	1
Portugal	0	0	1	1	1	0	0
Romania	0	6	11	11	11	17	0
Russia	0	93	46	46	46	232	46
Rwanda	0	1	0	0	0	1	1
Sierra Leone	1	1	4	4	4	7	2
South Africa	0	139	132	132	132	56	6
South Korea	0	11	12	12	12	16	4
Spain	0	1	0	0	0	0	0
Swaziland	0	0	0	0	0	1	0
Thailand	0	0	0	0	0	0	0
Turkmenistan	0	1	1	1	1	0	0
Uganda	1	7	18	18	18	18	1
United Kingdom	10	21	45	45	45	31	1
Uzbekistan	0	115	56	56	56	60	7
Vietnam	0	0	0	0	0	0	0

resistance mutation	counts	no_data	susceptible	resistant
EMB_SNP_CN_4247429_AG_embB_M306V	814	123	185	506
EMB_SNP_CN_4247431_GA_embB_M306I	719	122	185	412
EMB_SNP_CN_4247431_GC_embB_M306I	719	122	185	412
EMB_SNP_CN_4247431_GT_embB_M306I	719	122	185	412
EMB_SNP_CN_4242182_GT_embC_A774S	291	33	89	169
EMB_SNP_CN_4247574_AC_embB_D354A	236	6	108	122
EMB_unknown	234	0	0	234
EMB_SNP_CN_4248003_AG_embB_Q497R	164	42	40	82
EMB_SNP_CN_4247730_GC_embB_G406A	129	20	33	76
EMB_SNP_CN_409569_GA_iniB_A70T	117	19	25	73
EMB_SNP_CN_4247729_GA_embB_G406S	71	9	14	48
EMB_SNP_CN_4249583_GA_embB_D1024N	56	7	17	32
EMB_SNP_CN_4243392_AG_embA_N54D	50	8	1	41
EMB_SNP_P_4243222_CA.11_embA.embB	41	11	7	23
EMB_SNP_CN_4247429_AC_embB_M306L	34	7	8	19
EMB_embB_G406D_u	31	0	0	31
EMB_SNP_P_4243225_CT.8_embA.embB	28	4	3	21
EMB_upstream_intergenic-embA-embB_C16G_u	22	0	0	22
EMB_SNP_CN_4249518_AG_embB_H1002R	19	0	4	15
EMB_SNP_CN_4247495_GT_embB_D328Y	17	3	2	12
EMB_upstream_intergenic-embA-embB_C12T_u	17	0	0	17
EMB_embB_D328G_u	12	0	0	12
EMB_embB_L74R_u	12	0	0	12
EMB_embB_Q497K_u	12	0	0	12
EMB_upstream_intergenic-embA-embB_C16T_u	10	0	0	10
EMB_embB_E405D_u	8	0	0	8
EMB_embB_N296H_u	8	0	0	8
EMB_embB_S297A_u	7	0	0	7
EMB_upstream_intergenic-embA-embB_G43C_u	7	0	0	7
EMB_SNP_CN_4247729_GT_embB_G406C	6	3	1	2
EMB_embB_A409P_u	5	0	0	5
EMB_upstream_intergenic-embA-embB_C16A_u	5	0	0	5
EMB_embB_Q445R_u	4	0	0	4
EMB_embB_Q497P_u	4	0	0	4
EMB_embB_Y319C_u	4	0	0	4
EMB_embB_Y319S_u	4	0	0	4
EMB_embB_G603R_u	3	0	0	3
EMB_embB_I450L_u	3	0	0	3
EMB_embB_T1082A_u	3	0	0	3
EMB_embB_T642A_u	3	0	0	3
EMB_embA_D808A_u	2	0	0	2
EMB_embA_I905V_u	2	0	0	2
EMB_embA_L535V_u	2	0	0	2
EMB_embA_S654L_u	2	0	0	2
EMB_embA_V534G_u	2	0	0	2
EMB_embB_E504D_u	2	0	0	2
EMB_embB_S347I_u	2	0	0	2
EMB_embB_V50A_u	2	0	0	2
EMB_embC_M351T_u	2	0	0	2
EMB_iniB_A182V_u	2	0	0	2
EMB_upstream_intergenic-embA-embB_G76C_u	2	0	0	2
EMB_embA_G554D_u	1	0	0	1
EMB_embA_G884D_u	1	0	0	1
EMB_embA_T652R_u	1	0	0	1
EMB_embA_V31I_u	1	0	0	1
EMB_embA_V391L_u	1	0	0	1
EMB_embA_V479M_u	1	0	0	1
EMB_embB_C361Y_u	1	0	0	1
EMB_embB_D1056A_u	1	0	0	1
EMB_embB_D300G_u	1	0	0	1
EMB_embB_F323L_u	1	0	0	1
EMB_embB_I1009L_u	1	0	0	1
EMB_embB_I72L_u	1	0	0	1
EMB_embB_I72S_u	1	0	0	1
EMB_embB_L402V_u	1	0	0	1
EMB_embB_M306T_u	1	0	0	1
EMB_embB_M396I_u	1	0	0	1
EMB_embB_N399T_u	1	0	0	1
EMB_embB_N644I_u	1	0	0	1
EMB_embB_P404S_u	1	0	0	1
EMB_embB_P731L_u	1	0	0	1
EMB_embB_S347T_u	1	0	0	1
EMB_embB_S412P_u	1	0	0	1
EMB_embB_S538P_u	1	0	0	1
EMB_embB_T546I_u	1	0	0	1
EMB_embB_T581A_u	1	0	0	1
EMB_embB_T643I_u	1	0	0	1
EMB_embB_W332R_u	1	0	0	1
EMB_embB_Y319D_u	1	0	0	1
EMB_embC_A68T_u	1	0	0	1
EMB_embC_C411Y_u	1	0	0	1
EMB_embC_I10T_u	1	0	0	1
EMB_iniB_G386D_u	1	0	0	1
EMB_upstream_intergenic-embA-embB_C15G_u	1	0	0	1
EMB_upstream_intergenic-embA-embB_d42CAT_u	1	0	0	1
EMB_upstream_intergenic-embA-embB_d43G_u	1	0	0	1
EMB_upstream_intergenic-embA-embB_i.14-5TACCATCGAG_u	1	0	0	1

Ethionamide (ETH) Data, Abbreviations: counts = how many isolates had this mutation, no_data = how many isolates had this mutation but no data on whether they were phenotypically resistant or not to ETH, susceptible = how many isolates had this mutation but were phenotypically susceptible to ETH, resistant = how many isolates had this mutation but were resistant to ETH

country	ETH_SNP_CN_1674263_TC_inhA_I21T	ETH_SNP_CN_1674481_TG_inhA_S94A	ETH_SNP_CN_4326333_CG_ethA_A381P	ETH_SNP_P_1673423_GT.17_fabG1.inhA	ETH_SNP_P_1673425_CT.15_fabG1.inhA	ETH_othersnp	ETH_unknown
Azerbaijan	0	0	0	0	0	0	0
Bangladesh	0	0	0	0	0	0	0
Belarus	0	0	0	0	30	0	0
Brazil	0	0	0	0	0	0	0
Burma	0	0	0	0	0	0	0
Canada	0	0	0	0	0	0	0
China	0	1	0	0	18	13	15
Colombia	0	0	0	1	0	0	0
Democratic Republic of the Congo	0	0	0	0	0	0	0
Denmark	0	0	0	0	0	0	0
Djibouti	0	0	0	0	0	0	0
Dominican Republic	0	0	0	0	0	0	0
Estonia	0	0	0	0	0	0	0
Georgia	0	0	0	0	1	0	0
Germany	0	3	0	0	21	0	0
Guinea	0	0	0	0	2	0	0
India	0	0	0	0	1	0	0
Indonesia	0	0	0	0	0	0	0
Iran	0	0	0	0	0	0	0
Kazakhstan	0	0	0	0	0	0	0
Malawi	1	11	0	0	15	0	0
Mali	0	1	0	0	2	0	0
Moldova	0	0	0	0	25	0	0
Morocco	0	0	0	0	0	0	0
Nepal	0	0	0	0	0	0	0
Netherlands	0	0	0	0	0	0	0
Nigeria	0	0	0	0	0	0	0
Not Provided	12	98	12	14	573	51	46
Pakistan	0	0	0	0	1	0	0
Peru	33	20	1	27	178	100	76
Philippines	0	0	0	0	0	0	0
Portugal	0	0	0	0	0	0	0
Romania	0	0	0	0	7	0	0
Russia	0	4	0	2	50	0	3
Rwanda	0	0	0	0	1	0	0
Sierra Leone	0	1	0	1	1	0	0
South Africa	8	2	76	37	113	11	6
South Korea	0	2	0	0	8	6	10
Spain	0	1	0	0	1	0	0
Swaziland	0	0	0	0	0	0	0
Thailand	0	0	0	0	0	0	0
Turkmenistan	0	0	0	0	0	0	0
Uganda	0	0	0	0	4	0	1
United Kingdom	2	4	1	6	76	1	0
Uzbekistan	1	0	0	1	9	0	1
Vietnam	0	0	0	0	1	0	0

resistance mutation	counts	no_data	susceptible	resistant
ETH_SNP_P_1673425_CT.15_fabG1.inhA	1138	716	157	265
ETH_unknown	158	0	0	158
ETH_SNP_CN_1674481_TG_inhA_S94A	148	52	13	83
ETH_SNP_CN_4326333_CG_ethA_A381P	90	21	35	34
ETH_SNP_P_1673423_GT.17_fabG1.inhA	89	32	38	19
ETH_SNP_CN_1674263_TC_inhA_I21T	57	18	10	29
ETH_SNP_CN_4326116_GA_ethA_T453I	35	6	14	15
ETH_SNP_CN_4327380_AC_ethA_Y32D	15	2	9	4
ETH_upstream_intergenic-fabG1-inhA_T8A_u	14	0	0	14
ETH_DEL_F_4326184_d1289G_ethA	12	5	4	3
ETH_SNP_CN_4327416_CA_ethA_A20S	12	5	4	3
ETH_ethA_P378L_u	9	0	0	9
ETH_INS_F_4326722_i751C_ethA	6	2	1	3
ETH_SNP_CN_4326305_GA_ethA_S390F	6	2	1	3
ETH_SNP_CN_4326713_TG_ethA_Q254P	6	1	0	5
ETH_upstream_intergenic-fabG1-inhA_T8C_u	6	0	0	6
ETH_upstream_intergenic-fabG1-inhA_T8G_u	4	0	0	4
ETH_ethA_S266R_u	3	0	0	3
ETH_ethA_C403R_u	2	0	0	2
ETH_ethA_E400D_u	2	0	0	2
ETH_ethA_F302L_u	2	0	0	2
ETH_ethA_S110W_u	2	0	0	2
ETH_ethA_Y50C_u	2	0	0	2
ETH_SNP_CN_4327311_AG_ethA_S55P	1	0	1	0
ETH_ethA_A76V_u	1	0	0	1
ETH_ethA_F320S_u	1	0	0	1
ETH_ethA_F431V_u	1	0	0	1
ETH_ethA_G139C_u	1	0	0	1
ETH_ethA_G139S_u	1	0	0	1
ETH_ethA_G139V_u	1	0	0	1
ETH_ethA_G182S_u	1	0	0	1
ETH_ethA_G423R_u	1	0	0	1
ETH_ethA_G43C_u	1	0	0	1
ETH_ethA_G450D_u	1	0	0	1
ETH_ethA_H166P_u	1	0	0	1
ETH_ethA_L134P_u	1	0	0	1
ETH_ethA_L194P_u	1	0	0	1
ETH_ethA_L205P_u	1	0	0	1
ETH_ethA_L374R_u	1	0	0	1
ETH_ethA_L397R_u	1	0	0	1
ETH_ethA_N379D_u	1	0	0	1
ETH_ethA_P149S_u	1	0	0	1
ETH_ethA_P257A_u	1	0	0	1
ETH_ethA_P257S_u	1	0	0	1
ETH_ethA_P51H_u	1	0	0	1
ETH_ethA_P51L_u	1	0	0	1
ETH_ethA_P51S_u	1	0	0	1
ETH_ethA_Q206E_u	1	0	0	1
ETH_ethA_Q246R_u	1	0	0	1
ETH_ethA_R38P_u	1	0	0	1
ETH_ethA_R404L_u	1	0	0	1
ETH_ethA_R54S_u	1	0	0	1
ETH_ethA_S18R_u	1	0	0	1
ETH_ethA_S208P_u	1	0	0	1
ETH_ethA_S57F_u	1	0	0	1
ETH_ethA_T189K_u	1	0	0	1
ETH_ethA_T342K_u	1	0	0	1
ETH_ethA_T383P_u	1	0	0	1
ETH_ethA_T392R_u	1	0	0	1
ETH_ethA_T44P_u	1	0	0	1
ETH_ethA_T61M_u	1	0	0	1
ETH_ethA_Y235D_u	1	0	0	1
ETH_fabG1_L85V_u	1	0	0	1
ETH_inhA_I194T_u	1	0	0	1
ETH_inhA_I21V_u	1	0	0	1
ETH_inhA_I95L_u	1	0	0	1
ETH_upstream_intergenic-fabG1-inhA_C34T_u	1	0	0	1
ETH_INS_F_4326141_i1332C_ethA	0	0	0	0
ETH_SNP_CN_1673449_AC_fabG1_T4P	0	0	0	0
ETH_SNP_CN_1674434_TG_inhA_V78G	0	0	0	0
ETH_SNP_CN_4326278_GT_ethA_S399.	0	0	0	0
ETH_SNP_CN_4326600_GA_ethA_R292.	0	0	0	0
ETH_SNP_CN_4326714_GA_ethA_Q254.	0	0	0	0
ETH_SNP_CN_4327148_CT_ethA_W109.	0	0	0	0

Isoniazid (INH) Data, Abbreviations: counts = how many isolates had this mutation, no_data = how many isolates had this mutation but no data on whether they were phenotypically resistant or not to INH, susceptible = how many isolates had this mutation but were phenotypically susceptible to INH, resistant = how many isolates had this mutation but were resistant to INH

country	INH_SNP_CN_1674481_TG_inhA_S94A	INH_SNP_CN_2155168_CG_katG_S315T	INH_SNP_CN_2518919_GA_kasA_G269S	INH_SNP_CN_409569_GA_iniB_A70T	INH_SNP_P_1673425_CT.15_fabG1.inhA	INH_othersnp	INH_unknown
Azerbaijan	0	1	0	0	0	0	0
Bangladesh	0	1	0	0	0	0	2
Belarus	0	101	23	0	30	43	1
Brazil	0	0	0	0	0	3	0
Burma	0	1	0	0	0	0	0
Canada	0	0	0	0	0	0	0
China	1	65	0	0	18	24	4
Colombia	0	0	0	0	0	1	0
Democratic Republic of the Congo	0	0	0	0	0	0	0
Denmark	0	0	0	0	0	0	0
Djibouti	0	0	0	0	0	0	0
Dominican Republic	0	0	0	0	0	0	0
Estonia	0	2	0	0	0	0	0
Georgia	0	5	0	0	1	0	0
Germany	3	43	24	2	21	4	4
Guinea	0	0	0	0	2	0	0
India	0	8	0	0	1	1	0
Indonesia	0	0	0	0	0	0	0
Iran	0	11	0	0	0	0	1
Kazakhstan	0	2	0	0	0	0	0
Malawi	11	50	24	0	15	4	12
Mali	1	23	0	0	2	5	0
Moldova	0	43	3	0	25	0	0
Morocco	0	1	0	0	0	0	0
Nepal	0	4	0	0	0	0	0
Netherlands	0	3	0	0	0	0	1
Nigeria	0	1	0	0	0	0	0
Not Provided	98	539	120	27	573	102	82
Pakistan	0	0	0	0	1	0	0
Peru	20	593	299	88	178	80	40
Philippines	0	0	0	0	0	0	2
Portugal	0	1	0	0	0	0	0
Romania	0	30	1	0	7	5	0
Russia	4	470	61	0	50	20	49
Rwanda	0	1	0	0	1	1	2
Sierra Leone	1	16	8	0	1	3	2
South Africa	2	299	98	0	113	57	13
South Korea	2	35	0	0	8	3	6
Spain	1	1	0	0	1	0	0
Swaziland	0	0	0	0	0	1	0
Thailand	0	0	0	0	0	0	0
Turkmenistan	0	6	0	0	0	0	0
Uganda	0	35	1	0	4	3	2
United Kingdom	4	173	34	0	76	26	13
Uzbekistan	0	226	11	0	9	24	5
Vietnam	0	0	0	0	1	0	0

resistance mutation	counts	no_data	susceptible	resistant
INH_SNP_CN_2155168_CG_katG_S315T	2790	210	310	2270
INH_SNP_P_1673425_CT.15_fabG1.inhA	1138	424	100	614
INH_SNP_CN_2518919_GA_kasA_G269S	707	58	248	401
INH_unknown	241	0	0	241
INH_SNP_CN_1674481_TG_inhA_S94A	148	25	10	113
INH_SNP_CN_409569_GA_iniB_A70T	117	19	9	89
INH_SNP_P_1673432_TC.8_fabG1.inhA	102	33	36	33
INH_SNP_P_1673423_GT.17_fabG1.inhA	89	8	12	69
INH_SNP_CN_2155168_CT_katG_S315N	58	5	5	48
INH_SNP_CN_2155167_GT_katG_S315R	56	3	5	48
INH_SNP_P_1673432_TG.8_fabG1.inhA	10	0	1	9
INH_inhA_I21T_u	3	0	0	3
INH_katG_G279D_u	3	0	0	3
INH_katG_R104Q_u	3	0	0	3
INH_katG_S315G_u	3	0	0	3
INH_katG_W161R_u	3	0	0	3
INH_katG_D735Y_u	2	0	0	2
INH_katG_L141F_u	2	0	0	2
INH_katG_L141S_u	2	0	0	2
INH_katG_N138H_u	2	0	0	2
INH_katG_W191R_u	2	0	0	2
INH_katG_Y337C_u	2	0	0	2
INH_katG_Y413C_u	2	0	0	2
INH_ahpC_P44R_u	1	0	0	1
INH_inhA_K118E_u	1	0	0	1
INH_iniB_A222T_u	1	0	0	1
INH_iniB_G131R_u	1	0	0	1
INH_iniB_G171D_u	1	0	0	1
INH_iniB_G386D_u	1	0	0	1
INH_iniB_S249R_u	1	0	0	1
INH_kasA_A45S_u	1	0	0	1
INH_kasA_R161S_u	1	0	0	1
INH_katG_A109V_u	1	0	0	1
INH_katG_A139P_u	1	0	0	1
INH_katG_A162E_u	1	0	0	1
INH_katG_A614E_u	1	0	0	1
INH_katG_D142G_u	1	0	0	1
INH_katG_D163N_u	1	0	0	1
INH_katG_D189A_u	1	0	0	1
INH_katG_D259Y_u	1	0	0	1
INH_katG_D282G_u	1	0	0	1
INH_katG_D419H_u	1	0	0	1
INH_katG_D487N_u	1	0	0	1
INH_katG_D656A_u	1	0	0	1
INH_katG_D695A_u	1	0	0	1
INH_katG_D94G_u	1	0	0	1
INH_katG_F408L_u	1	0	0	1
INH_katG_G120S_u	1	0	0	1
INH_katG_G182R_u	1	0	0	1
INH_katG_G273S_u	1	0	0	1
INH_katG_G285S_u	1	0	0	1
INH_katG_G297V_u	1	0	0	1
INH_katG_G299S_u	1	0	0	1
INH_katG_G630R_u	1	0	0	1
INH_katG_G699V_u	1	0	0	1
INH_katG_G99R_u	1	0	0	1
INH_katG_K537E_u	1	0	0	1
INH_katG_L159F_u	1	0	0	1
INH_katG_L159P_u	1	0	0	1
INH_katG_L598F_u	1	0	0	1
INH_katG_L76P_u	1	0	0	1
INH_katG_N138D_u	1	0	0	1
INH_katG_P232R_u	1	0	0	1
INH_katG_P325S_u	1	0	0	1
INH_katG_Q461P_u	1	0	0	1
INH_katG_R571H_u	1	0	0	1
INH_katG_S302R_u	1	0	0	1
INH_katG_S315I_u	1	0	0	1
INH_katG_T180K_u	1	0	0	1
INH_katG_T271I_u	1	0	0	1
INH_katG_T475I_u	1	0	0	1
INH_katG_V320L_u	1	0	0	1
INH_katG_V423I_u	1	0	0	1
INH_katG_V442A_u	1	0	0	1
INH_katG_V626E_u	1	0	0	1
INH_katG_V633A_u	1	0	0	1
INH_katG_W191G_u	1	0	0	1
INH_katG_W300C_u	1	0	0	1
INH_katG_W300R_u	1	0	0	1
INH_katG_W300S_u	1	0	0	1
INH_katG_W328L_u	1	0	0	1
INH_katG_W341G_u	1	0	0	1
INH_katG_W438G_u	1	0	0	1
INH_katG_W90R_u	1	0	0	1
INH_katG_Y413H_u	1	0	0	1
INH_katG_Y608F_u	1	0	0	1
INH_katG_Y98C_u	1	0	0	1
INH_upstream_intergenic-fabG1-inhA_G102A_u	1	0	0	1
INH_upstream_intergenic-fabG1-inhA_T8A_u	1	0	0	1
INH_SNP_CN_2726338_TG_ahpC_V49G	0	0	0	0

Kanamycin (KAN) Data, Abbreviations: counts = how many isolates had this mutation, no_data = how many isolates had this mutation but no data on whether they were phenotypically resistant or not to KAN, susceptible = how many isolates had this mutation but were phenotypically susceptible to KAN, resistant = how many isolates had this mutation but were resistant to KAN

country	KAN_SNP_N_1473246_A1401G_rrs	KAN_othersnp	KAN_rrs_A514C_u	KAN_rrs_A906G_u	KAN_rrs_C513T_u	KAN_rrs_G1484T_u	KAN_unknown
Azerbaijan	0	0	0	0	0	0	0
Bangladesh	0	0	0	0	0	0	0
Belarus	51	0	0	0	0	0	0
Brazil	0	0	0	0	0	0	0
Burma	0	0	0	0	0	0	0
Canada	0	0	0	0	0	0	0
China	14	1	2	0	0	0	6
Colombia	1	0	0	0	0	0	0
Democratic Republic of the Congo	0	0	0	0	0	0	0
Denmark	0	0	0	0	0	0	0
Djibouti	0	0	0	0	0	0	0
Dominican Republic	0	0	0	0	0	0	0
Estonia	0	0	0	0	0	0	0
Georgia	3	0	0	0	0	0	0
Germany	3	0	0	0	0	0	0
Guinea	0	0	0	0	0	0	0
India	0	0	0	0	0	0	0
Indonesia	0	0	0	0	0	0	0
Iran	3	0	0	0	0	0	0
Kazakhstan	0	0	0	0	0	0	0
Malawi	0	0	0	0	0	0	0
Mali	0	0	0	0	0	0	0
Moldova	0	0	0	0	0	0	0
Morocco	0	0	0	0	0	0	0
Nepal	0	0	0	0	0	0	0
Netherlands	0	0	0	0	0	0	0
Nigeria	0	0	0	0	0	0	0
Not Provided	160	6	3	0	3	7	53
Pakistan	0	0	0	0	0	0	0
Peru	110	4	1	5	4	0	59
Philippines	0	0	0	0	0	0	0
Portugal	0	0	0	0	0	0	0
Romania	12	0	0	0	0	0	2
Russia	35	0	0	0	0	0	0
Rwanda	2	0	0	0	0	0	0
Sierra Leone	0	0	0	0	0	0	0
South Africa	164	2	2	0	0	0	17
South Korea	26	0	0	1	0	0	24
Spain	0	0	0	0	0	0	0
Swaziland	0	0	0	0	0	0	0
Thailand	0	0	0	0	0	0	0
Turkmenistan	0	0	0	0	0	0	0
Uganda	1	0	0	0	0	0	0
United Kingdom	8	0	0	0	0	0	4
Uzbekistan	46	0	0	0	0	0	0
Vietnam	0	0	0	0	0	0	0

resistance mutation	counts	no_data	susceptible	resistant
KAN_SNP_N_1473246_A1401G_rrs	639	208	83	348
KAN_unknown	165	0	0	165
KAN_rrs_A514C_u	8	0	0	8
KAN_rrs_C513T_u	7	0	0	7
KAN_rrs_G1484T_u	7	0	0	7
KAN_rrs_A906G_u	6	0	0	6
KAN_rrs_C517T_u	4	0	0	4
KAN_rrs_C905A_u	2	0	0	2
KAN_rrs_A365G_u	1	0	0	1
KAN_rrs_A514T_u	1	0	0	1
KAN_rrs_A908C_u	1	0	0	1
KAN_rrs_A908G_u	1	0	0	1
KAN_rrs_C1105G_u	1	0	0	1
KAN_rrs_C1402A_u	1	0	0	1
KAN_rrs_G878A_u	1	0	0	1
KAN_SNP_CN_1918745_AG_tlyA_.269W	0	0	0	0

Levofloxacin (LEVO) Data, Abbreviations: counts = how many isolates had this mutation, no_data = how many isolates had this mutation but no data on whether they were phenotypically resistant or not to LEVO, susceptible = how many isolates had this mutation but were phenotypically susceptible to LEVO, resistant = how many isolates had this mutation but were resistant to LEVO

country	LEVO_SNP_CN_7570_CT_gyrA_A90V	LEVO_SNP_CN_7572_TC_gyrA_S91P	LEVO_SNP_CN_7581_GA_gyrA_D94N	LEVO_SNP_CN_7582_AC_gyrA_D94A	LEVO_SNP_CN_7582_AG_gyrA_D94G	LEVO_othersnp	LEVO_unknown
Azerbaijan	0	0	0	0	0	0	0
Bangladesh	0	0	0	0	0	0	0
Belarus	23	6	2	11	21	5	0
Brazil	0	0	0	0	0	0	0
Burma	0	0	0	1	0	0	0
Canada	0	0	0	0	0	0	0
China	11	4	1	7	18	4	0
Colombia	0	0	0	0	0	0	0
Democratic Republic of the Congo	0	0	0	0	0	0	0
Denmark	0	0	0	0	0	0	0
Djibouti	0	0	0	0	0	0	0
Dominican Republic	0	0	0	0	0	0	0
Estonia	0	0	0	0	1	0	0
Georgia	0	0	0	0	0	0	0
Germany	0	2	1	1	2	0	0
Guinea	0	0	0	0	0	0	0
India	2	0	0	0	2	1	0
Indonesia	0	0	0	0	0	0	0
Iran	3	0	0	1	6	0	0
Kazakhstan	0	0	0	0	0	0	0
Malawi	0	0	0	0	0	0	0
Mali	1	0	0	0	0	0	0
Moldova	0	0	0	0	0	1	0
Morocco	0	0	0	0	0	0	0
Nepal	0	0	0	0	0	0	0
Netherlands	0	0	0	0	1	0	0
Nigeria	0	0	0	0	0	0	0
Not Provided	57	36	12	25	86	19	10
Pakistan	1	0	0	0	0	0	0
Peru	20	1	8	9	29	10	3
Philippines	0	0	0	0	0	0	0
Portugal	0	0	0	0	0	0	0
Romania	2	0	0	3	1	1	0
Russia	10	5	7	12	31	4	0
Rwanda	1	0	0	0	0	0	0
Sierra Leone	0	0	0	0	0	0	0
South Africa	78	4	6	21	44	12	0
South Korea	13	3	2	2	14	1	0
Spain	0	0	0	0	0	0	0
Swaziland	0	0	0	0	0	0	0
Thailand	0	0	0	0	0	0	0
Turkmenistan	0	0	0	0	0	0	0
Uganda	0	1	0	0	0	0	0
United Kingdom	7	7	2	1	30	1	0
Uzbekistan	0	0	0	0	0	0	0
Vietnam	0	0	0	0	0	0	0

resistance mutation	counts	no_data	susceptible	resistant
LEVO_SNP_CN_7582_AG_gyrA_D94G	286	270	0	16
LEVO_SNP_CN_7570_CT_gyrA_A90V	229	214	2	13
LEVO_SNP_CN_7582_AC_gyrA_D94A	94	91	0	3
LEVO_SNP_CN_7572_TC_gyrA_S91P	69	66	0	3
LEVO_SNP_CN_7581_GA_gyrA_D94N	41	39	0	2
LEVO_SNP_CN_7581_GT_gyrA_D94Y	34	33	0	1
LEVO_unknown	13	0	0	13
LEVO_SNP_CN_6735_AC_gyrB_N538T	10	8	1	1
LEVO_SNP_CN_7563_GT_gyrA_G88C	8	7	0	1
LEVO_SNP_CN_7566_GA_gyrA_D89N	6	5	1	0
LEVO_gyrA_R128K_u	1	0	0	1

Ofloxacin (OFLX) Data, Abbreviations: counts = how many isolates had this mutation, no_data = how many isolates had this mutation but no data on whether they were phenotypically resistant or not to OFLX, susceptible = how many isolates had this mutation but were phenotypically susceptible to OFLX, resistant = how many isolates had this mutation but were resistant to OFLX

country	OFLX_SNP_CN_7570_CT_gyrA_A90V	OFLX_SNP_CN_7581_GA_gyrA_D94N	OFLX_SNP_CN_7582_AC_gyrA_D94A	OFLX_SNP_CN_7582_AG_gyrA_D94G	OFLX_gyrA_S91P_u	OFLX_othersnp	OFLX_unknown
Azerbaijan	0	0	0	0	0	0	0
Bangladesh	0	0	0	0	0	0	0
Belarus	23	2	11	21	0	3	0
Brazil	0	0	0	0	0	0	0
Burma	0	0	1	0	0	0	0
Canada	0	0	0	0	0	0	0
China	11	1	7	18	3	6	13
Colombia	0	0	0	0	0	0	0
Democratic Republic of the Congo	0	0	0	0	0	0	0
Denmark	0	0	0	0	0	0	0
Djibouti	0	0	0	0	0	0	0
Dominican Republic	0	0	0	0	0	0	0
Estonia	0	0	0	1	0	0	0
Georgia	0	0	0	0	0	0	0
Germany	0	1	1	2	0	1	0
Guinea	0	0	0	0	0	0	0
India	2	0	0	2	0	0	0
Indonesia	0	0	0	0	0	0	0
Iran	3	0	1	6	0	0	0
Kazakhstan	0	0	0	0	0	0	0
Malawi	0	0	0	0	0	0	0
Mali	1	0	0	0	0	0	0
Moldova	0	0	0	0	0	0	0
Morocco	0	0	0	0	0	0	0
Nepal	0	0	0	0	0	0	0
Netherlands	0	0	0	1	0	0	0
Nigeria	0	0	0	0	0	0	0
Not Provided	57	12	25	86	26	49	97
Pakistan	1	0	0	0	0	0	0
Peru	20	8	9	29	0	3	3
Philippines	0	0	0	0	0	0	0
Portugal	0	0	0	0	0	0	0
Romania	2	0	3	1	0	4	2
Russia	10	7	12	31	5	11	57
Rwanda	1	0	0	0	0	0	0
Sierra Leone	0	0	0	0	0	0	0
South Africa	78	6	21	44	1	22	16
South Korea	13	2	2	14	0	0	0
Spain	0	0	0	0	0	0	0
Swaziland	0	0	0	0	0	0	0
Thailand	0	0	0	0	0	0	0
Turkmenistan	0	0	0	0	0	0	0
Uganda	0	0	0	0	1	1	0
United Kingdom	7	2	1	30	3	1	0
Uzbekistan	0	0	0	0	0	0	7
Vietnam	0	0	0	0	0	0	0

resistance mutation	counts	no_data	susceptible	resistant
OFLX_SNP_CN_7582_AG_gyrA_D94G	286	89	30	167
OFLX_SNP_CN_7570_CT_gyrA_A90V	229	62	75	92
OFLX_unknown	195	0	0	195
OFLX_SNP_CN_7582_AC_gyrA_D94A	94	19	23	52
OFLX_SNP_CN_7581_GA_gyrA_D94N	41	14	5	22
OFLX_gyrA_S91P_u	39	0	0	39
OFLX_gyrA_D94Y_u	22	0	0	22
OFLX_SNP_CN_7581_GC_gyrA_D94H	14	3	5	6
OFLX_SNP_CN_6735_AC_gyrB_N538T	10	6	1	3
OFLX_gyrA_G88C_u	4	0	0	4
OFLX_gyrB_D500N_u	4	0	0	4
OFLX_gyrB_V340L_u	4	0	0	4
OFLX_gyrA_Q613E_u	3	0	0	3
OFLX_gyrB_D500H_u	3	0	0	3
OFLX_gyrA_A74S_u	2	0	0	2
OFLX_gyrA_N282K_u	2	0	0	2
OFLX_gyrA_T267I_u	2	0	0	2
OFLX_gyrB_E540D_u	2	0	0	2
OFLX_gyrB_P133L_u	2	0	0	2
OFLX_gyrB_S486F_u	2	0	0	2
OFLX_gyrA_A288D_u	1	0	0	1
OFLX_gyrA_A463S_u	1	0	0	1
OFLX_gyrA_A90G_u	1	0	0	1
OFLX_gyrA_D829E_u	1	0	0	1
OFLX_gyrA_D89N_u	1	0	0	1
OFLX_gyrA_D94V_u	1	0	0	1
OFLX_gyrA_E214D_u	1	0	0	1
OFLX_gyrA_G88A_u	1	0	0	1
OFLX_gyrA_P472S_u	1	0	0	1
OFLX_gyrA_R292G_u	1	0	0	1
OFLX_gyrA_R382L_u	1	0	0	1
OFLX_gyrA_R448H_u	1	0	0	1
OFLX_gyrA_R592S_u	1	0	0	1
OFLX_gyrA_T135S_u	1	0	0	1
OFLX_gyrB_A471V_u	1	0	0	1
OFLX_gyrB_A543T_u	1	0	0	1
OFLX_gyrB_H350Y_u	1	0	0	1
OFLX_gyrB_I39V_u	1	0	0	1
OFLX_gyrB_N538D_u	1	0	0	1
OFLX_gyrB_N538Y_u	1	0	0	1
OFLX_gyrB_P439R_u	1	0	0	1
OFLX_gyrB_R485C_u	1	0	0	1
OFLX_gyrB_T539N_u	1	0	0	1
OFLX_gyrB_V496L_u	1	0	0	1
OFLX_gyrB_V600A_u	1	0	0	1

Para-aminosalicylic acid (PAS) Data, Abbreviations: counts = how many isolates had this mutation, no_data = how many isolates had this mutation but no data on whether they were phenotypically resistant or not to PAS, susceptible = how many isolates had this mutation but were phenotypically susceptible to PAS, resistant = how many isolates had this mutation but were resistant to PAS

country	PAS_SNP_CN_3073852_TC_thyA_H207R	PAS_SNP_CN_3074182_TC_thyA_Q97R	PAS_SNP_CN_3074449_AT_thyA_L8Q	PAS_inter-thyX-hsdS.1_G228A_u	PAS_othersnp	PAS_thyA_T202A_u	PAS_unknown
Azerbaijan	0	0	0	0	0	0	0
Bangladesh	0	0	0	0	0	0	0
Belarus	0	0	0	0	0	0	0
Brazil	0	0	0	0	0	0	0
Burma	0	0	0	0	0	0	0
Canada	0	0	0	0	0	0	0
China	1	0	0	0	0	0	0
Colombia	0	0	0	0	0	0	0
Democratic Republic of the Congo	0	0	0	0	0	0	0
Denmark	0	0	0	0	0	0	0
Djibouti	0	0	0	0	0	0	0
Dominican Republic	0	0	0	0	0	0	0
Estonia	0	0	0	0	0	0	0
Georgia	0	0	0	0	0	0	0
Germany	0	0	0	0	0	0	0
Guinea	0	0	0	0	0	0	0
India	0	0	0	0	0	0	0
Indonesia	0	0	0	0	0	0	0
Iran	0	0	0	0	0	0	0
Kazakhstan	0	0	0	0	0	0	0
Malawi	0	0	0	0	0	0	0
Mali	0	0	0	0	0	0	0
Moldova	0	0	0	0	0	0	0
Morocco	0	0	0	0	0	0	0
Nepal	0	0	0	0	0	0	0
Netherlands	0	0	0	0	0	0	0
Nigeria	0	0	0	0	0	0	0
Not Provided	5	2	4	3	15	18	5
Pakistan	0	0	0	0	0	0	0
Peru	10	0	4	0	1	0	0
Philippines	0	0	0	0	0	0	0
Portugal	0	0	0	0	0	0	0
Romania	0	0	0	0	0	0	0
Russia	0	1	0	0	1	0	0
Rwanda	0	0	0	0	0	0	0
Sierra Leone	0	0	0	0	0	0	0
South Africa	0	0	0	1	2	0	3
South Korea	0	0	0	6	6	0	3
Spain	0	0	0	0	0	0	0
Swaziland	0	0	0	0	0	0	0
Thailand	0	0	0	0	0	0	0
Turkmenistan	0	0	0	0	0	0	0
Uganda	0	0	0	0	0	0	0
United Kingdom	0	1	0	0	0	0	0
Uzbekistan	0	0	0	0	0	0	0
Vietnam	0	0	0	0	0	0	0

resistance mutation	counts	no_data	susceptible	resistant
PAS_thyA_T202A_u	18	0	0	18
PAS_SNP_CN_3073852_TC_thyA_H207R	16	14	0	2
PAS_unknown	11	0	0	11
PAS_upstream_intergenic-thyX-hsdS.1_G228A_u	10	0	0	10
PAS_SNP_CN_3074449_AT_thyA_L8Q	8	7	0	1
PAS_SNP_CN_3074182_TC_thyA_Q97R	4	3	0	1
PAS_folC_R49Q_u	2	0	0	2
PAS_folC_R49W_u	2	0	0	2
PAS_upstream_intergenic-thyX-hsdS.1_C235T_u	2	0	0	2
PAS_upstream_intergenic-thyA_G24A_u	2	0	0	2
PAS_folC_E153G_u	1	0	0	1
PAS_folC_I43S_u	1	0	0	1
PAS_folC_I43T_u	1	0	0	1
PAS_folC_I43V_u	1	0	0	1
PAS_folC_S98G_u	1	0	0	1
PAS_upstream_intergenic-thyX-hsdS.1_C226A_u	1	0	0	1
PAS_upstream_intergenic-thyX-hsdS.1_G240A_u	1	0	0	1
PAS_upstream_intergenicr-thyA_T117C_u	1	0	0	1
PAS_upstream_intergenic-thyA_d49CCGCAGCGACTCGCCGCCAAACAAACCCAGCGGGCGATCGCAAGCGCGGCGAAGCCG_u	1	0	0	1
PAS_thyA_A262V_u	1	0	0	1
PAS_thyA_D6G_u	1	0	0	1
PAS_thyA_D81A_u	1	0	0	1
PAS_thyA_E137G_u	1	0	0	1
PAS_thyA_E58V_u	1	0	0	1
PAS_thyA_F152V_u	1	0	0	1
PAS_thyA_P145L_u	1	0	0	1
PAS_thyA_T22P_u	1	0	0	1
PAS_SNP_P_3074479_AG.157_thyA	0	0	0	0

Pyrazinamide (PZA) Data, Abbreviations: counts = how many isolates had this mutation, no_data = how many isolates had this mutation but no data on whether they were phenotypically resistant or not to PZA, susceptible = how many isolates had this mutation but were phenotypically susceptible to PZA, resistant = how many isolates had this mutation but were resistant to PZA

country	PZA_SNP_CN_2289090_TC_pncA_H51R	PZA_SNP_CN_2289099_TG_pncA_K48T	PZA_SNP_CN_2289213_TC_pncA_Q10R	PZA_SNP_CN_2289213_TG_pncA_Q10P	PZA_othersnp	PZA_promoter-pncA_T11C_u	PZA_unknown
Azerbaijan	0	0	0	1	0	0	0
Bangladesh	0	0	0	0	0	0	0
Belarus	0	0	2	0	56	0	0
Brazil	0	0	0	0	0	0	0
Burma	0	0	0	0	1	0	0
Canada	0	0	0	0	0	0	0
China	0	0	0	2	22	0	0
Colombia	0	0	0	0	0	0	0
Democratic Republic of the Congo	0	0	0	0	0	0	0
Denmark	0	0	0	0	0	0	0
Djibouti	0	0	0	0	0	0	0
Dominican Republic	0	0	0	0	0	0	0
Estonia	0	0	0	0	0	0	0
Georgia	0	0	0	0	0	0	0
Germany	0	0	0	0	14	0	5
Guinea	0	0	0	0	1	0	0
India	0	0	0	0	2	0	0
Indonesia	0	0	0	0	0	0	0
Iran	0	0	0	0	4	0	1
Kazakhstan	0	0	0	0	0	0	0
Malawi	0	0	0	0	4	0	0
Mali	0	0	0	0	3	0	0
Moldova	0	0	0	2	6	0	0
Morocco	0	0	0	0	0	0	0
Nepal	0	0	0	0	0	0	0
Netherlands	0	0	0	0	0	0	0
Nigeria	0	0	0	0	0	0	0
Not Provided	10	12	6	6	260	15	78
Pakistan	0	0	0	0	0	0	0
Peru	79	35	45	9	296	8	150
Philippines	0	0	0	0	0	0	0
Portugal	0	0	0	0	0	0	0
Romania	0	0	0	0	20	0	0
Russia	2	0	0	1	121	6	71
Rwanda	0	0	0	0	0	0	1
Sierra Leone	0	0	0	0	4	1	3
South Africa	0	0	0	3	112	0	66
South Korea	0	0	0	0	23	0	24
Spain	0	0	0	0	0	0	0
Swaziland	0	0	0	0	0	0	0
Thailand	0	0	0	0	0	0	0
Turkmenistan	0	0	0	0	0	1	2
Uganda	0	0	0	0	12	1	10
United Kingdom	0	0	0	0	41	2	19
Uzbekistan	1	0	2	39	69	17	40
Vietnam	0	0	0	0	0	0	0

resistance mutation	counts	no_data	susceptible	resistant
PZA_unknown	470	0	0	470
PZA_SNP_CN_2289090_TC_pncA_H51R	92	12	9	71
PZA_SNP_CN_2289213_TG_pncA_Q10P	63	11	4	48
PZA_SNP_CN_2289213_TC_pncA_Q10R	55	4	5	46
PZA_upstream_intergenic-pncA_T11C_u	51	0	0	51
PZA_SNP_CN_2289099_TG_pncA_K48T	47	9	22	16
PZA_SNP_CN_2289202_AG_pncA_C14R	46	12	0	34
PZA_SNP_CN_2288844_AG_pncA_I133T	40	1	16	23
PZA_SNP_CN_2289016_TG_pncA_T76P	38	23	2	13
PZA_SNP_CN_2288883_AG_pncA_L120P	35	15	0	20
PZA_SNP_CN_2288839_TG_pncA_T135P	34	12	2	20
PZA_SNP_CN_2289040_AC_pncA_W68G	31	22	5	4
PZA_SNP_CN_2289073_GC_pncA_H57D	26	5	0	21
PZA_SNP_CN_2289231_AG_pncA_L4S	25	8	0	17
PZA_SNP_CN_2288826_AG_pncA_V139A	23	5	7	11
PZA_SNP_CN_2289142_AC_pncA_Y34D	22	19	1	2
PZA_SNP_CN_2288820_TG_pncA_Q141P	20	7	2	11
PZA_SNP_CN_2289081_GA_pncA_P54L	20	4	4	12
PZA_SNP_CN_2289180_AC_pncA_V21G	20	5	4	11
PZA_pncA_V125G_u	20	0	0	20
PZA_SNP_CN_2289207_TG_pncA_D12A	19	6	4	9
PZA_SNP_CN_2288727_AG_pncA_L172P	17	11	2	4
PZA_SNP_CN_2289097_CT_pncA_D49N	16	3	1	12
PZA_SNP_CN_2288952_CT_pncA_G97D	15	9	0	6
PZA_SNP_CN_2288953_CT_pncA_G97S	15	3	1	11
PZA_SNP_CN_2289040_AG_pncA_W68R	15	4	2	9
PZA_DEL_F_2288939_d302TCCGGTGTAG_pncA	13	9	0	4
PZA_SNP_CN_2288805_GA_pncA_A146V	13	9	0	4
PZA_SNP_CN_2289054_TG_pncA_D63A	12	2	3	7
PZA_SNP_CN_2289070_AG_pncA_F58L	12	4	3	5
PZA_SNP_CN_2288805_GT_pncA_A146E	11	0	4	7
PZA_SNP_CN_2289207_TC_pncA_D12G	11	1	2	8
PZA_pncA_I6L_u	11	0	0	11
PZA_DEL_F_2289069_d172A_pncA_F58L	10	6	0	4
PZA_SNP_CN_2288703_AC_pncA_V180G	10	2	0	8
PZA_SNP_CN_2288764_TC_pncA_T160A	10	3	0	7
PZA_SNP_CN_2288827_CT_pncA_V139M	10	3	1	6
PZA_SNP_CN_2289220_CT_pncA_D8N	10	6	0	4
PZA_SNP_CN_2288704_CA_pncA_V180F	9	0	2	7
PZA_INS_F_2289009_i232C_pncA_G78G	8	3	1	4
PZA_INS_F_2289050_i191T_pncA_Y64.	8	3	1	4
PZA_SNP_CN_2288850_AC_pncA_V131G	8	3	1	4
PZA_SNP_CN_2288859_AC_pncA_V128G	8	1	2	5
PZA_upstream_intergenic-pncA_T11G_u	8	0	0	8
PZA_SNP_CN_2288778_AC_pncA_V155G	7	3	1	3
PZA_SNP_CN_2288943_GA_pncA_T100I	7	0	1	6
PZA_SNP_CN_2288988_AG_pncA_L85P	7	2	1	4
PZA_SNP_CN_2289043_AG_pncA_S67P	7	1	0	6
PZA_SNP_CN_2289072_TC_pncA_H57R	7	2	0	5
PZA_SNP_CN_2288696_CA_pncA_L182F	6	0	5	1
PZA_SNP_CN_2288835_TC_pncA_D136G	6	3	1	2
PZA_SNP_CN_2288965_CA_pncA_V93L	6	0	1	5
PZA_SNP_CN_2289072_TA_pncA_H57L	6	0	1	5
PZA_SNP_CN_2289103_TC_pncA_T47A	6	2	3	1
PZA_DEL_F_2288923_d318C_pncA	5	2	1	2
PZA_INS_F_2288825_i416C_pncA	5	1	0	4
PZA_INS_F_2288851_i390CC_pncA	5	1	1	3
PZA_INS_F_2288942_i299T_pncA	5	2	1	2
PZA_SNP_CN_2288697_AG_pncA_L182S	5	2	2	1
PZA_SNP_CN_2288718_AG_pncA_M175T	5	2	0	3
PZA_SNP_CN_2288938_CG_pncA_A102P	5	2	2	1
PZA_SNP_CN_2288944_TG_pncA_T100P	5	1	0	4
PZA_SNP_CN_2288956_TC_pncA_K96E	5	1	0	4
PZA_SNP_CN_2288973_AG_pncA_I90T	5	1	1	3
PZA_SNP_CN_2289028_AG_pncA_C72R	5	2	1	2
PZA_SNP_CN_2289030_TC_pncA_H71R	5	0	3	2
PZA_SNP_CN_2289162_AG_pncA_L27P	5	1	2	2
PZA_SNP_CN_2289219_TC_pncA_D8G	5	2	1	2
PZA_pncA_C138R_u	5	0	0	5
PZA_pncA_D8E_u	5	0	0	5
PZA_pncA_S164P_u	5	0	0	5
PZA_INS_F_2288851_i390C_pncA	4	2	0	2
PZA_INS_F_2288887_i354A_pncA	4	2	0	2
PZA_SNP_CN_2288766_AC_pncA_L159R	4	0	0	4
PZA_SNP_CN_2288818_TC_pncA_T142A	4	2	0	2
PZA_SNP_CN_2288841_GA_pncA_A134V	4	2	0	2
PZA_SNP_CN_2288955_TG_pncA_K96T	4	1	0	3
PZA_SNP_CN_2289037_GA_pncA_P69S	4	0	4	0
PZA_pncA_F13L_u	4	0	0	4
PZA_pncA_M1T_u	4	0	0	4
PZA_pncA_R140P_u	4	0	0	4
PZA_pncA_V7G_u	4	0	0	4
PZA_DEL_F_2288776_d465GCACCCTG_pncA	3	1	1	1
PZA_INS_F_2288725_i516C_pncA	3	1	0	2
PZA_SNP_CN_2288775_AG_pncA_L156P	3	2	0	1
PZA_SNP_CN_2288883_AC_pncA_L120R	3	1	0	2
PZA_SNP_CN_2289001_AC_pncA_F81V	3	0	2	1
PZA_SNP_CN_2289015_GA_pncA_T76I	3	1	1	1
PZA_SNP_CN_2289054_TC_pncA_D63G	3	0	1	2
PZA_SNP_CN_2289073_GA_pncA_H57Y	3	0	0	3
PZA_pncA_C14G_u	3	0	0	3
PZA_pncA_D136Y_u	3	0	0	3
PZA_pncA_H51Q_u	3	0	0	3
PZA_pncA_H82R_u	3	0	0	3
PZA_pncA_L116R_u	3	0	0	3
PZA_pncA_L4W_u	3	0	0	3
PZA_pncA_P54Q_u	3	0	0	3
PZA_pncA_P62S_u	3	0	0	3
PZA_upstream_intergenic-pncA_A12G_u	3	0	0	3

PZA_upstream_intergenic-pncA_A12G_u	3	0	0	3
PZA_SNP_CN_2288718_AC_pncA_M175R	2	1	0	1
PZA_SNP_CN_2288742_GA_pncA_T167I	2	0	1	1
PZA_SNP_CN_2288818_TG_pncA_T142P	2	1	0	1
PZA_SNP_CN_2288826_AC_pncA_V139G	2	0	0	2
PZA_SNP_CN_2288869_CA_pncA_V125F	2	0	1	1
PZA_SNP_CN_2288887_AC_pncA_W119G	2	0	0	2
PZA_SNP_CN_2288919_CT_pncA_G108E	2	0	1	1
PZA_SNP_CN_2288925_AG_pncA_F106S	2	1	0	1
PZA_SNP_CN_2288964_AC_pncA_V93G	2	0	1	1
PZA_SNP_CN_2289009_CA_pncA_G78V	2	0	0	2
PZA_SNP_CN_2289030_TG_pncA_H71P	2	1	0	1
PZA_SNP_CN_2289046_AG_pncA_S66P	2	1	0	1
PZA_SNP_CN_2289090_TG_pncA_H51P	2	0	1	1
PZA_SNP_CN_2289091_GA_pncA_H51Y	2	2	0	0
PZA_SNP_CN_2289095_GC_pncA_D49E	2	1	0	1
PZA_SNP_CN_2289212_CG_pncA_Q10H	2	0	0	2
PZA_SNP_CN_2289216_AC_pncA_V9G	2	1	0	1
PZA_SNP_CN_2289228_AG_pncA_I5T	2	0	0	2
PZA_pncA_A46V_u	2	0	0	2
PZA_pncA_D63H_u	2	0	0	2
PZA_pncA_D8A_u	2	0	0	2
PZA_pncA_F94L_u	2	0	0	2
PZA_pncA_G97C_u	2	0	0	2
PZA_pncA_H71Q_u	2	0	0	2
PZA_pncA_H71Y_u	2	0	0	2
PZA_pncA_H82D_u	2	0	0	2
PZA_pncA_I6M_u	2	0	0	2
PZA_pncA_K48E_u	2	0	0	2
PZA_pncA_L35R_u	2	0	0	2
PZA_pncA_M175V_u	2	0	0	2
PZA_pncA_P62L_u	2	0	0	2
PZA_pncA_P69R_u	2	0	0	2
PZA_pncA_S104R_u	2	0	0	2
PZA_pncA_V130M_u	2	0	0	2
PZA_pncA_V155M_u	2	0	0	2
PZA_pncA_V44G_u	2	0	0	2
PZA_pncA_Y103C_u	2	0	0	2
PZA_pncA_Y95S_u	2	0	0	2
PZA_upstream_intergenic-pncA_A7G_u	2	0	0	2
PZA_INS_F_2288835_i406T_pncA	1	0	0	1
PZA_SNP_CN_2288697_AC_pncA_L182W	1	1	0	0
PZA_SNP_CN_2288730_GA_pncA_A171V	1	1	0	0
PZA_SNP_CN_2288817_GA_pncA_T142M	1	1	0	0
PZA_SNP_CN_2288847_CT_pncA_G132D	1	0	0	1
PZA_SNP_CN_2288935_AG_pncA_Y103H	1	0	0	1
PZA_SNP_CN_2289042_GC_pncA_S67W	1	0	0	1
PZA_SNP_CN_2289069_AC_pncA_F58C	1	0	0	1
PZA_SNP_CN_2289186_AG_pncA_L19P	1	0	0	1
PZA_pncA_A102T_u	1	0	0	1
PZA_pncA_A146P_u	1	0	0	1
PZA_pncA_A171E_u	1	0	0	1
PZA_pncA_A171T_u	1	0	0	1
PZA_pncA_A3E_u	1	0	0	1
PZA_pncA_A46E_u	1	0	0	1
PZA_pncA_A46P_u	1	0	0	1
PZA_pncA_C14W_u	1	0	0	1
PZA_pncA_D129Y_u	1	0	0	1
PZA_pncA_D12N_u	1	0	0	1
PZA_pncA_D136N_u	1	0	0	1
PZA_pncA_D49G_u	1	0	0	1
PZA_pncA_F13I_u	1	0	0	1
PZA_pncA_F58S_u	1	0	0	1
PZA_pncA_F81C_u	1	0	0	1
PZA_pncA_G105D_u	1	0	0	1
PZA_pncA_G108R_u	1	0	0	1
PZA_pncA_G132A_u	1	0	0	1
PZA_pncA_G162D_u	1	0	0	1
PZA_pncA_G17D_u	1	0	0	1
PZA_pncA_G24D_u	1	0	0	1
PZA_pncA_G78C_u	1	0	0	1
PZA_pncA_G78S_u	1	0	0	1
PZA_pncA_G97R_u	1	0	0	1
PZA_pncA_H43P_u	1	0	0	1
PZA_pncA_I133S_u	1	0	0	1
PZA_pncA_I6T_u	1	0	0	1
PZA_pncA_K96R_u	1	0	0	1
PZA_pncA_L151S_u	1	0	0	1
PZA_pncA_L156Q_u	1	0	0	1
PZA_pncA_L159V_u	1	0	0	1
PZA_pncA_L85R_u	1	0	0	1
PZA_pncA_M175I_u	1	0	0	1
PZA_pncA_M175K_u	1	0	0	1
PZA_pncA_M1I_u	1	0	0	1
PZA_pncA_P62R_u	1	0	0	1
PZA_pncA_R154G_u	1	0	0	1
PZA_pncA_R2W_u	1	0	0	1
PZA_pncA_S104G_u	1	0	0	1
PZA_pncA_S32I_u	1	0	0	1
PZA_pncA_T100A_u	1	0	0	1
PZA_pncA_T114P_u	1	0	0	1
PZA_pncA_T160K_u	1	0	0	1
PZA_pncA_T160P_u	1	0	0	1
PZA_pncA_T168P_u	1	0	0	1
PZA_pncA_V139L_u	1	0	0	1
PZA_pncA_V155A_u	1	0	0	1
PZA_pncA_V163A_u	1	0	0	1
PZA_pncA_V7F_u	1	0	0	1
PZA_pncA_V7L_u	1	0	0	1
PZA_pncA_W119R_u	1	0	0	1
PZA_pncA_W68C_u	1	0	0	1
PZA_pncA_W68L_u	1	0	0	1
PZA_pncA_Y103D_u	1	0	0	1
PZA_pncA_Y64D_u	1	0	0	1
PZA_upstream_intergenic-pncA_T11A_u	1	0	0	1
PZA_upstream_intergenic-pncA_T15C_u	1	0	0	1
PZA_upstream_intergenic-pncA_T16C_u	1	0	0	1
PZA_upstream_intergenic-pncA_d10A_u	1	0	0	1
PZA_upstream_intergenic-pncA_i14T_u	1	0	0	1
PZA_DEL_F_2288697_d544AACT_pncA	0	0	0	0
PZA_DEL_F_2289060_d181GTGCCGGA_pncA	0	0	0	0
PZA_DEL_N_2288942_d299GGTGTA_pncA	0	0	0	0
PZA_SNP_CN_2288784_GT_pncA_T153N	0	0	0	0
PZA_SNP_CN_2288848_CT_pncA_G132S	0	0	0	0
PZA_SNP_CN_2288853_AC_pncA_V130G	0	0	0	0
PZA_SNP_CN_2288853_AT_pncA_V130E	0	0	0	0
PZA_SNP_CN_2288878_GA_pncA_Q122.	0	0	0	0
PZA_SNP_CN_2288933_GC_pncA_Y103.	0	0	0	0
PZA_SNP_CN_2288956_TG_pncA_K96Q	0	0	0	0
PZA_SNP_CN_2289042_GT_pncA_S67.	0	0	0	0
PZA_SNP_CN_2289050_AT_pncA_Y64.	0	0	0	0
PZA_SNP_CN_2289150_AC_pncA_I31S	0	0	0	0
PZA_SNP_CN_2289206_GC_pncA_D12E	0	0	0	0
PZA_SNP_CN_2289214_GA_pncA_Q10.	0	0	0	0
PZA_SNP_P_2289245_TA.37_pncA	0	0	0	0
PZA_SNP_P_2289251_AC.31_pncA	0	0	0	0
PZA_SNP_P_2289252_TC.30_pncA	0	0	0	0
PZA_SNP_P_2289252_TG.30_pncA	0	0	0	0

Rifamycin (RIF) Data, Abbreviations: counts = how many isolates had this mutation, no_data = how many isolates had this mutation but no data on whether they were phenotypically resistant or not to RIF, susceptible = how many isolates had this mutation but were phenotypically susceptible to RIF, resistant = how many isolates had this mutation but were resistant to RIF

country	RIF_SNP_CN_761109_GT_rpoB_D435Y	RIF_SNP_CN_761110_AT_rpoB_D435V	RIF_SNP_CN_761139_CT_rpoB_H445Y	RIF_SNP_CN_761155_CT_rpoB_S450L	RIF_SNP_CN_761161_TC_rpoB_L452P	RIF_othersnp	RIF_unknown
Azerbaijan	0	0	0	1	0	0	0
Bangladesh	0	0	0	0	0	0	1
Belarus	3	0	1	77	0	20	0
Brazil	0	0	0	0	0	0	0
Burma	0	0	0	1	0	0	0
Canada	0	0	0	0	0	0	0
China	3	4	11	57	9	36	5
Colombia	0	0	0	0	0	0	1
Democratic Republic of the Congo	0	0	0	0	0	0	0
Denmark	0	0	0	0	0	0	0
Djibouti	0	0	0	0	0	0	0
Dominican Republic	0	0	0	0	0	0	0
Estonia	0	0	0	2	0	0	0
Georgia	0	0	0	4	1	0	0
Germany	0	1	1	13	1	5	1
Guinea	0	0	0	1	0	0	0
India	0	2	0	8	0	0	0
Indonesia	0	0	0	0	0	0	0
Iran	0	0	0	10	0	2	1
Kazakhstan	0	0	0	2	0	0	0
Malawi	0	1	0	4	0	1	1
Mali	0	8	0	4	3	5	0
Moldova	1	0	0	34	0	4	0
Morocco	0	0	0	0	0	1	0
Nepal	0	0	0	1	0	1	0
Netherlands	0	0	0	0	0	0	0
Nigeria	0	0	0	1	0	0	0
Not Provided	42	73	49	515	18	123	57
Pakistan	0	0	0	1	0	0	0
Peru	22	134	28	491	10	110	57
Philippines	0	0	0	1	0	0	1
Portugal	0	0	0	1	0	0	0
Romania	6	4	2	19	0	3	0
Russia	6	7	3	350	6	28	74
Rwanda	0	1	0	3	0	0	1
Sierra Leone	2	0	3	6	1	2	1
South Africa	10	55	15	188	62	36	8
South Korea	4	3	2	35	1	1	3
Spain	0	0	0	1	1	0	0
Swaziland	0	0	0	2	0	0	0
Thailand	0	0	0	0	0	0	0
Turkmenistan	0	0	0	1	0	0	0
Uganda	1	3	2	25	0	14	4
United Kingdom	3	5	7	54	6	65	3
Uzbekistan	3	8	7	213	4	18	13
Vietnam	0	0	0	0	0	0	0

resistance mutation	counts	no_data	susceptible	resistant
RIF_SNP_CN_761155_CT_rpoB_S450L	2126	232	228	1666
RIF_SNP_CN_761110_AT_rpoB_D435V	309	19	27	263
RIF_unknown	232	0	0	232
RIF_SNP_CN_761139_CT_rpoB_H445Y	131	16	10	105
RIF_SNP_CN_761161_TC_rpoB_L452P	123	5	75	43
RIF_SNP_CN_761109_GT_rpoB_D435Y	106	18	22	66
RIF_SNP_CN_761139_CG_rpoB_H445D	99	21	10	68
RIF_SNP_CN_761140_AG_rpoB_H445R	59	9	3	47
RIF_SNP_CN_761155_CG_rpoB_S450W	50	3	15	32
RIF_SNP_CN_761140_AT_rpoB_H445L	40	7	4	29
RIF_SNP_CN_761095_TC_rpoB_L430P	37	3	20	14
RIF_SNP_CN_761140_AT_rpoB_I491F	36	8	18	10
RIF_SNP_CN_761277_AT_rpoB_I491F	36	8	18	10
RIF_SNP_CN_760314_GT_rpoB_V170F	29	13	1	15
RIF_rpoB_S450P_u	9	0	0	9
RIF_rpoB_Q432P_u	7	0	0	7
RIF_rpoB_S441L_u	7	0	0	7
RIF_rpoB_D435G_u	6	0	0	6
RIF_rpoB_Q432K_u	6	0	0	6
RIF_rpoB_R448Q_u	6	0	0	6
RIF_rpoB_E250G_u	4	0	0	4
RIF_rpoB_H445N_u	4	0	0	4
RIF_rpoB_V695L_u	4	0	0	4
RIF_rpoB_P454L_u	3	0	0	3
RIF_rpoB_Q432L_u	3	0	0	3
RIF_rpoB_D435N_u	2	0	0	2
RIF_rpoB_H445P_u	2	0	0	2
RIF_rpoB_L494P_u	2	0	0	2
RIF_rpoB_M434I_u	2	0	0	2
RIF_rpoB_Q432E_u	2	0	0	2
RIF_rpoB_A451G_u	1	0	0	1
RIF_rpoB_D435A_u	1	0	0	1
RIF_rpoB_G675D_u	1	0	0	1
RIF_rpoB_G981D_u	1	0	0	1
RIF_rpoB_H835Q_u	1	0	0	1
RIF_rpoB_H835R_u	1	0	0	1
RIF_rpoB_I925V_u	1	0	0	1
RIF_rpoB_K446Q_u	1	0	0	1
RIF_rpoB_L430R_u	1	0	0	1
RIF_rpoB_L443W_u	1	0	0	1
RIF_rpoB_M707T_u	1	0	0	1
RIF_rpoB_P280L_u	1	0	0	1
RIF_rpoB_P439S_u	1	0	0	1
RIF_rpoB_S428R_u	1	0	0	1
RIF_rpoB_S441T_u	1	0	0	1
RIF_rpoB_T400A_u	1	0	0	1
RIF_rpoB_T400P_u	1	0	0	1
RIF_rpoB_T444P_u	1	0	0	1
RIF_rpoB_V113I_u	1	0	0	1
RIF_rpoB_V469L_u	1	0	0	1
RIF_SNP_CN_761102_AC_rpoB_Q432H	0	0	0	0
RIF_SNP_CN_761155_CA_rpoB_S450.	0	0	0	0

Streptomycin (STR) Data, Abbreviations: counts = how many isolates had this mutation, no_data = how many isolates had this mutation but no data on whether they were phenotypically resistant or not to RIF, susceptible = how many isolates had this mutation but were phenotypically susceptible to STR, resistant = how many isolates had this mutation but were resistant to STR

country	STR_SNP_CN_4407967_AG_gid_L79S	STR_SNP_CN_781687_AG_rpsL_K43R	STR_SNP_CN_781822_AG_rpsL_K88R	STR_SNP_N_1472359_A514C_rrs	STR_SNP_N_1472362_C517T_rrs	STR_othersnp	STR_unknown
Azerbaijan	0	0	0	0	1	0	0
Bangladesh	0	0	1	0	0	0	1
Belarus	0	63	6	20	10	1	0
Brazil	0	1	0	0	0	0	1
Burma	0	1	0	0	0	0	0
Canada	0	0	0	0	0	0	0
China	0	46	16	10	2	4	7
Colombia	0	0	0	0	0	0	0
Democratic Republic of the Congo	0	0	0	0	0	0	0
Denmark	0	0	0	0	0	0	0
Djibouti	0	0	0	0	0	0	0
Dominican Republic	0	0	0	0	0	0	0
Estonia	0	2	0	0	0	0	0
Georgia	0	5	0	0	0	0	0
Germany	3	29	3	3	2	38	11
Guinea	0	0	0	0	1	0	0
India	0	9	1	0	1	2	2
Indonesia	0	0	0	0	0	0	0
Iran	0	8	0	1	3	0	1
Kazakhstan	0	2	0	0	0	0	0
Malawi	0	4	6	0	0	18	3
Mali	17	0	1	1	0	1	1
Moldova	0	13	27	0	1	2	0
Morocco	0	0	0	0	0	0	1
Nepal	0	2	0	1	0	0	0
Netherlands	0	9	1	1	7	0	0
Nigeria	0	0	0	0	0	0	1
Not Provided	15	239	29	39	20	244	50
Pakistan	0	0	0	0	1	0	0
Peru	2	71	8	14	7	340	96
Philippines	0	0	0	0	0	0	1
Portugal	0	1	0	0	0	0	0
Romania	0	9	2	2	0	6	5
Russia	0	221	43	17	184	19	47
Rwanda	0	0	0	0	0	0	1
Sierra Leone	0	9	7	0	0	13	4
South Africa	74	72	15	83	7	9	15
South Korea	0	12	1	2	0	3	3
Spain	0	0	0	0	0	0	1
Swaziland	0	0	1	0	0	0	0
Thailand	0	0	0	0	0	0	0
Turkmenistan	0	5	1	0	0	0	0
Uganda	0	2	3	0	2	13	9
United Kingdom	5	53	10	16	5	55	5
Uzbekistan	0	212	12	13	3	13	5
Vietnam	0	2	0	0	0	0	0

resistance mutation	counts	no_data	susceptible	resistant
STR_SNP_CN_781687_AG_rpsL_K43R	1102	172	112	818
STR_unknown	271	0	0	271
STR_SNP_N_1472362_C517T_rrs	257	14	54	189
STR_SNP_N_1472359_A514C_rrs	223	54	25	144
STR_SNP_CN_781822_AG_rpsL_K88R	194	22	44	128
STR_SNP_CN_4407967_AG_gid_L79S	116	27	13	76
STR_SNP_CN_4407967_AC_gid_L79W	73	15	26	32
STR_SNP_CN_781822_AC_rpsL_K88T	55	8	6	41
STR_SNP_CN_4407934_AC_gid_L90R	35	6	10	19
STR_SNP_CN_4407768_CA_gid_L145F	34	3	22	9
STR_SNP_N_1472358_C513T_rrs	34	6	2	26
STR_SNP_N_1472751_A906G_rrs	29	3	8	18
STR_DEL_F_4407640_d562A_gid	28	0	17	11
STR_SNP_CN_4408094_CT_gid_G37R	28	0	17	11
STR_DEL_F_4407852_d350C_gid	27	19	5	3
STR_SNP_CN_4407995_TG_gid_S70R	27	19	5	3
STR_SNP_CN_4407985_CG_gid_G73A	23	0	8	15
STR_SNP_CN_4407809_CA_gid_D132Y	21	5	1	15
STR_SNP_CN_4407916_CA_gid_R96L	16	3	5	8
STR_gid_L50R_u	14	0	0	14
STR_SNP_N_1472753_A908C_rrs	11	0	3	8
STR_SNP_CN_4407748_AG_gid_L152S	10	1	5	4
STR_gid_P84L_u	10	0	0	10
STR_SNP_CN_4408064_GA_gid_R47W	9	2	5	2
STR_rrs_A514T_u	8	0	0	8
STR_SNP_CN_4407832_AG_gid_V124A	7	0	2	5
STR_SNP_CN_781822_AT_rpsL_K88M	7	1	2	4
STR_gid_G76C_u	7	0	0	7
STR_gid_A138V_u	6	0	0	6
STR_gid_D67G_u	6	0	0	6
STR_gid_L26F_u	6	0	0	6
STR_SNP_CN_4408148_CG_gid_A19P	5	0	2	3
STR_gid_H48Y_u	5	0	0	5
STR_gid_P75R_u	5	0	0	5
STR_gid_R137P_u	5	0	0	5
STR_gid_V77G_u	5	0	0	5
STR_SNP_CN_4408102_CT_gid_G34E	4	2	0	2
STR_gid_A10P_u	4	0	0	4
STR_gid_S149R_u	4	0	0	4
STR_SNP_CN_4407947_GA_gid_L86F	3	0	3	0
STR_SNP_CN_4408060_TG_gid_H48P	3	1	1	1
STR_gid_A134E_u	3	0	0	3
STR_gid_A138E_u	3	0	0	3
STR_gid_A80P_u	3	0	0	3
STR_gid_D85A_u	3	0	0	3
STR_gid_E92Q_u	3	0	0	3
STR_gid_G69D_u	3	0	0	3
STR_gid_L86P_u	3	0	0	3
STR_gid_P75S_u	3	0	0	3
STR_gid_R118S_u	3	0	0	3
STR_gid_T146K_u	3	0	0	3
STR_gid_V77A_u	3	0	0	3
STR_rrs_C905A_u	3	0	0	3
STR_SNP_N_1473167_T1322G_rrs	2	0	0	2
STR_gid_A200E_u	2	0	0	2
STR_gid_E170G_u	2	0	0	2
STR_gid_E40A_u	2	0	0	2
STR_gid_G117E_u	2	0	0	2
STR_gid_G117V_u	2	0	0	2
STR_gid_G30R_u	2	0	0	2
STR_gid_G34A_u	2	0	0	2
STR_gid_G34V_u	2	0	0	2
STR_gid_G37E_u	2	0	0	2
STR_gid_H48N_u	2	0	0	2
STR_gid_L101F_u	2	0	0	2
STR_gid_L108R_u	2	0	0	2
STR_gid_L18H_u	2	0	0	2
STR_gid_L59R_u	2	0	0	2
STR_gid_P75L_u	2	0	0	2
STR_gid_R137W_u	2	0	0	2
STR_gid_R83G_u	2	0	0	2
STR_gid_R83W_u	2	0	0	2
STR_gid_V110A_u	2	0	0	2
STR_gid_V65G_u	2	0	0	2
STR_gid_W45S_u	2	0	0	2
STR_rpsL_K88Q_u	2	0	0	2
STR_SNP_CN_4408138_TC_gid_Y22C	1	0	0	1
STR_gid_A10V_u	1	0	0	1
STR_gid_A119D_u	1	0	0	1
STR_gid_A133P_u	1	0	0	1
STR_gid_A138P_u	1	0	0	1
STR_gid_A140S_u	1	0	0	1
STR_gid_A19G_u	1	0	0	1
STR_gid_A205E_u	1	0	0	1
STR_gid_A205T_u	1	0	0	1
STR_gid_A27P_u	1	0	0	1
STR_gid_A72V_u	1	0	0	1
STR_gid_A82E_u	1	0	0	1
STR_gid_C52F_u	1	0	0	1
STR_gid_D67A_u	1	0	0	1
STR_gid_E120K_u	1	0	0	1
STR_gid_E121D_u	1	0	0	1
STR_gid_G117R_u	1	0	0	1
STR_gid_G117W_u	1	0	0	1
STR_gid_G130A_u	1	0	0	1
STR_gid_G182R_u	1	0	0	1
STR_gid_G30D_u	1	0	0	1
STR_gid_G30V_u	1	0	0	1
STR_gid_G34R_u	1	0	0	1

STR_gid_G34W_u	1	0	0	1
STR_gid_G71R_u	1	0	0	1
STR_gid_H48D_u	1	0	0	1
STR_gid_H48Q_u	1	0	0	1
STR_gid_H48R_u	1	0	0	1
STR_gid_I11N_u	1	0	0	1
STR_gid_I162S_u	1	0	0	1
STR_gid_I81R_u	1	0	0	1
STR_gid_I81T_u	1	0	0	1
STR_gid_L108P_u	1	0	0	1
STR_gid_L142W_u	1	0	0	1
STR_gid_L160P_u	1	0	0	1
STR_gid_L79F_u	1	0	0	1
STR_gid_L86R_u	1	0	0	1
STR_gid_L91P_u	1	0	0	1
STR_gid_M150R_u	1	0	0	1
STR_gid_P78L_u	1	0	0	1
STR_gid_P93L_u	1	0	0	1
STR_gid_P93Q_u	1	0	0	1
STR_gid_P93R_u	1	0	0	1
STR_gid_R116W_u	1	0	0	1
STR_gid_R118C_u	1	0	0	1
STR_gid_R118L_u	1	0	0	1
STR_gid_R137L_u	1	0	0	1
STR_gid_R175P_u	1	0	0	1
STR_gid_R20P_u	1	0	0	1
STR_gid_R21W_u	1	0	0	1
STR_gid_R33P_u	1	0	0	1
STR_gid_R47G_u	1	0	0	1
STR_gid_R47P_u	1	0	0	1
STR_gid_R64W_u	1	0	0	1
STR_gid_R83P_u	1	0	0	1
STR_gid_R96C_u	1	0	0	1
STR_gid_S131T_u	1	0	0	1
STR_gid_S70N_u	1	0	0	1
STR_gid_T98I_u	1	0	0	1
STR_gid_V105E_u	1	0	0	1
STR_gid_V115G_u	1	0	0	1
STR_gid_V203L_u	1	0	0	1
STR_gid_V41I_u	1	0	0	1
STR_gid_V66A_u	1	0	0	1
STR_gid_V88A_u	1	0	0	1
STR_gid_V89G_u	1	0	0	1
STR_gid_Y195C_u	1	0	0	1
STR_gid_Y22S_u	1	0	0	1
STR_rpsL_E70A_u	1	0	0	1
STR_rpsL_E76A_u	1	0	0	1
STR_rrs_A1012G_u	1	0	0	1
STR_rrs_A1223G_u	1	0	0	1
STR_rrs_A1325C_u	1	0	0	1
STR_rrs_A170G_u	1	0	0	1
STR_rrs_A504C_u	1	0	0	1
STR_rrs_A700T_u	1	0	0	1
STR_rrs_A703G_u	1	0	0	1
STR_rrs_A753T_u	1	0	0	1
STR_rrs_A907C_u	1	0	0	1
STR_rrs_A907T_u	1	0	0	1
STR_rrs_A908G_u	1	0	0	1
STR_rrs_A948T_u	1	0	0	1
STR_rrs_C1067T_u	1	0	0	1
STR_rrs_C1199G_u	1	0	0	1
STR_rrs_C1390T_u	1	0	0	1
STR_rrs_C1402T_u	1	0	0	1
STR_rrs_C239A_u	1	0	0	1
STR_rrs_C244T_u	1	0	0	1
STR_rrs_C270T_u	1	0	0	1
STR_rrs_C332T_u	1	0	0	1
STR_rrs_C397T_u	1	0	0	1
STR_rrs_C414A_u	1	0	0	1
STR_rrs_C499T_u	1	0	0	1
STR_rrs_C546T_u	1	0	0	1
STR_rrs_C692T_u	1	0	0	1
STR_rrs_C708T_u	1	0	0	1
STR_rrs_C897T_u	1	0	0	1
STR_rrs_C905G_u	1	0	0	1
STR_rrs_C936T_u	1	0	0	1
STR_rrs_G1042C_u	1	0	0	1
STR_rrs_G1072A_u	1	0	0	1
STR_rrs_G1415T_u	1	0	0	1
STR_rrs_G319A_u	1	0	0	1
STR_rrs_G395C_u	1	0	0	1
STR_rrs_G407A_u	1	0	0	1
STR_rrs_G408T_u	1	0	0	1
STR_rrs_G537A_u	1	0	0	1
STR_rrs_G544A_u	1	0	0	1
STR_rrs_G685A_u	1	0	0	1
STR_rrs_G749C_u	1	0	0	1
STR_rrs_G771A_u	1	0	0	1
STR_rrs_G878A_u	1	0	0	1
STR_rrs_G887T_u	1	0	0	1
STR_rrs_G909T_u	1	0	0	1
STR_rrs_T1206C_u	1	0	0	1
STR_rrs_T1208A_u	1	0	0	1
STR_rrs_T1217A_u	1	0	0	1
STR_rrs_T16C_u	1	0	0	1
STR_rrs_T327C_u	1	0	0	1
STR_rrs_T411A_u	1	0	0	1
STR_rrs_T529G_u	1	0	0	1
STR_rrs_T545A_u	1	0	0	1
STR_rrs_T580C_u	1	0	0	1
STR_rrs_T672A_u	1	0	0	1
STR_rrs_T696G_u	1	0	0	1
STR_DEL_F_4408023_d179T_gid	0	0	0	0
STR_DEL_F_4408116_d86G_gid	0	0	0	0
STR_SNP_CN_4408091_GT_gid_P38T	0	0	0	0
STR_SNP_I_1473637_A.21_rrs.rrl	0	0	0	0
STR_SNP_N_1473109_T1264G_rrs	0	0	0	0
STR_SNP_N_1473343_G1498T_rrs	0	0	0	0

Supplementary Table 17: Median MRSCA with IQR for INH, STR, RIF, EMB, PZA, SLIs, ETH, FLQ, and CYS for 0.5 and 0.3 mutation rate.

Drug	Median MRSCA 0.5 Mutation Rate	Median MRSCA 0.3 Mutation Rate
INH	13.9 IQR 11.0 – 17.5	23.3 IQR 17.4 – 29.3
STR	7.9 IQR 4.1 – 12.9	13.0 IQR 6.4 – 20.7
RIF	6.6 IQR 2.9 – 14.4	10.4 IQR 4.8 – 24.0
EMB	5.9 IQR 2.5 – 13.5	8.1 IQR 3.7 – 19.3
PZA	3.2 IQR 1.9 – 7.8	5.4 IQR 3.2 – 11.7
SLIs	5.0 IQR 2.0 – 9.1	7.9 IQR 3.4 – 15.5
ETH	2.3 IQR 1.3 – 12.8	4.1 IQR 2.0 – 21.5
FLQ	4.4 IQR 1.8 – 10.4	6.5 IQR 2.7 – 16.9
CYS	1.6 IQR 1.2-2.0	2.7 IQR 2.1-3.5

Supplementary Table 18: P-value for Wilcoxon Rank-Sum Test between the MRSCA distributions of INH, RIF, STR, PZA, SLIs, ETH, EMB, FLQ, and CYS for 0.5 and 0.3 mutation rate.

P-value <= 0.01, highlighted in red

0.5 MR	INH	RIF	STR	PZA	SLIs	ETH	EMB	FLQ	CYS
INH		3 E-20	1 E-20	2 E-38	2 E-34	6 E-13	2E-19	4E-30	3E-8
RIF			0.06	9E-15	4 E-7	1 E-5	0.10	2 E-8	2E-5
STR				1E-21	1E-9	2 E-6	5 E-5	1 E-11	2 E-3
PZA					1 E-3	0.2	1E-4	0.31	4E-3
SLIs						0.03	0.01	0.25	1E-3
ETH							9 E-3	0.15	0.11
EMB								8E-4	1E-4
FLQ									0.01
CYS									

P-value <= 0.01, highlighted in red

0.3 MR	INH	RIF	STR	PZA	SLIs	ETH	EMB	FLQ	CYS
INH		7 E-14	1 E-9	9 E-31	5 E-25	0.22	2E-9	2E-12	6 E-6
RIF			0.08	1E-13	4 E-7	3E-5	0.15	9E-8	7 E-3
STR				1E-21	1E-11	2E-6	4 E-3	1 E-11	5 E-6
PZA					4E-3	0.20	2E-6	0.32	4E-3
SLIs						0.06	8E-3	0.55	2E-3
ETH							2E-3	0.16	0.13
EMB								8E-4	2E-4
FLQ									6E-3
CYS									

Supplementary Table 19: Median and IQR for MRSCA distribution for RIF and SLIs in China and Peru for 0.5 and 0.3 Mutation Rate.

Country	Drug	Median MRSCA 0.5 Mutation Rate	Median MRSCA 0.3 Mutation Rate
China	RIF	16.1 (IQR 12.7 – 19.3)	27.7 (IQR 21.1 – 30.7)
Peru	RIF	4.6 (IQR 2.1 – 13.7)	8.3 (IQR 3.5 – 21.8)
China	SLIs	9.9 (IQR 8.4 – 14.4)	17.0 (IQR 13.5 – 24.6)
Peru	SLIs	3.4 (IQR 1.7 – 8.8)	5.4 (IQR 2.6 – 14.4)

Supplementary Table 20: GDP per capita and Median MRSCA Date for RIF for 12 countries for 0.3 and 0.5 Mutation Rate.

Countries	GDP per capita	0.5 Mutation Rate	0.3 Mutation Rate
**China**	10150	16.1 (IQR 12.7 – 19.3)	27.7 (IQR 21.1 – 30.7)
**Peru**	7140	4.6 (IQR 2.1 – 13.7)	8.3 (IQR 3.5 – 21.8)
**Russia**	11190	5.7 (IQR 3.9 – 10.2)	8.8 (IQR 5.4 – 15.8)
**Mali**	933.88	3.4 (IQR 3.2 – 3.4)	5.2 (IQR 4.4 – 5.2)
**Moldova**	3400	3.8 (IQR 3.1 – 4.2)	6.3 (IQR 4.8 – 7.1)
**Malawi**	366.53	5.4 (IQR 5.4 – 5.4)	7.9 (IQR 7.9 – 7.9)
**Germany**	47790	16 (IQR 10.0 – 17.4)	28 (IQR 24.5 – 28.5)
**India**	2200	11 (IQR 11.2 – 13.8)	21 (IQR 18.7 – 23.6)
**Russia**	11190	5.7 (IQR 3.9 – 10.2)	8.8 (IQR 5.4 – 15.8)
**South Africa**	6330	4.53 (IQR 2.4 – 6.1)	7.1 (IQR 4.0 – 10.7)
**United Kingdom**	42310	11.9 (IQR 11.4 – 14.4)	19.5 (IQR 18.2 – 24.0)
**South Korea**	31940	17.7 (IQR 15.8 – 17.7)	28.5 (IQR 24.5 – 28.5)
**Sierra Leone**	516.76	5.2 (IQR 3.6 – 11.3)	8.4 (IQR 6.0 – 19.1)

Supplementary Figure 1: Distribution of STR MRSCA dates across all countries and lineages.

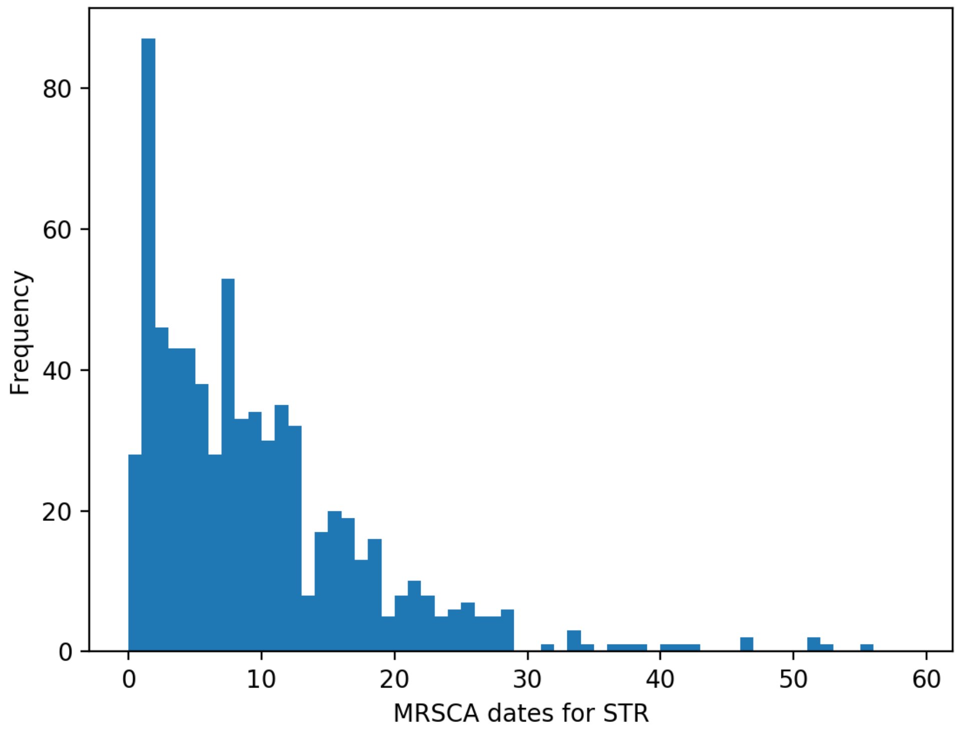

Supplementary Figure 2 Distribution of resistance mutations for six drugs (n=9385). Pie chart size is proportional isolate number from each country. Mutations listed in order of frequency.

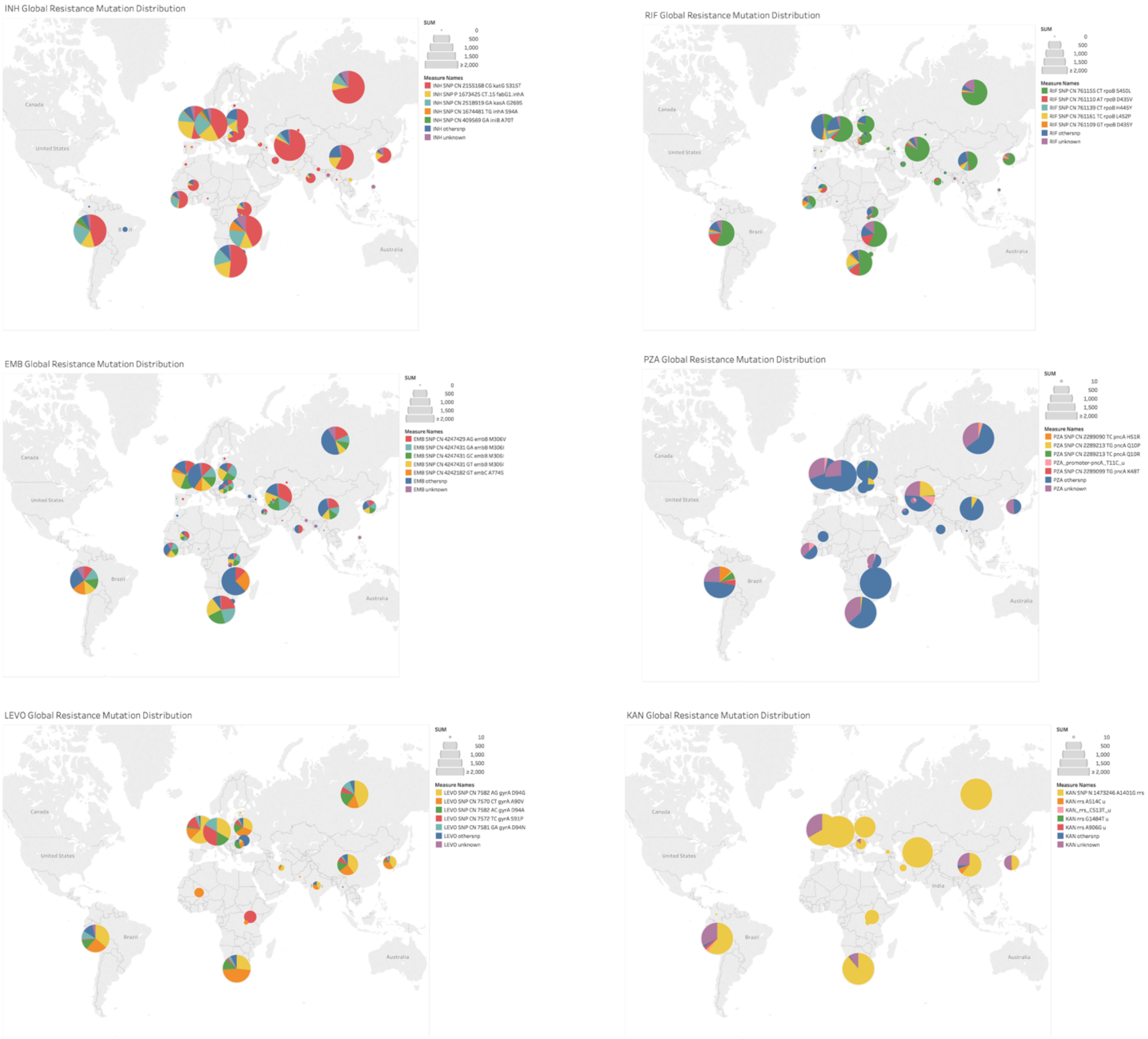

Supplementary Figure 3: MRSCA date distribution for PZA, EMB, FLQ, and SLIs for Peru, South Africa, and China for 0.3 and 0.5 mutation rate.

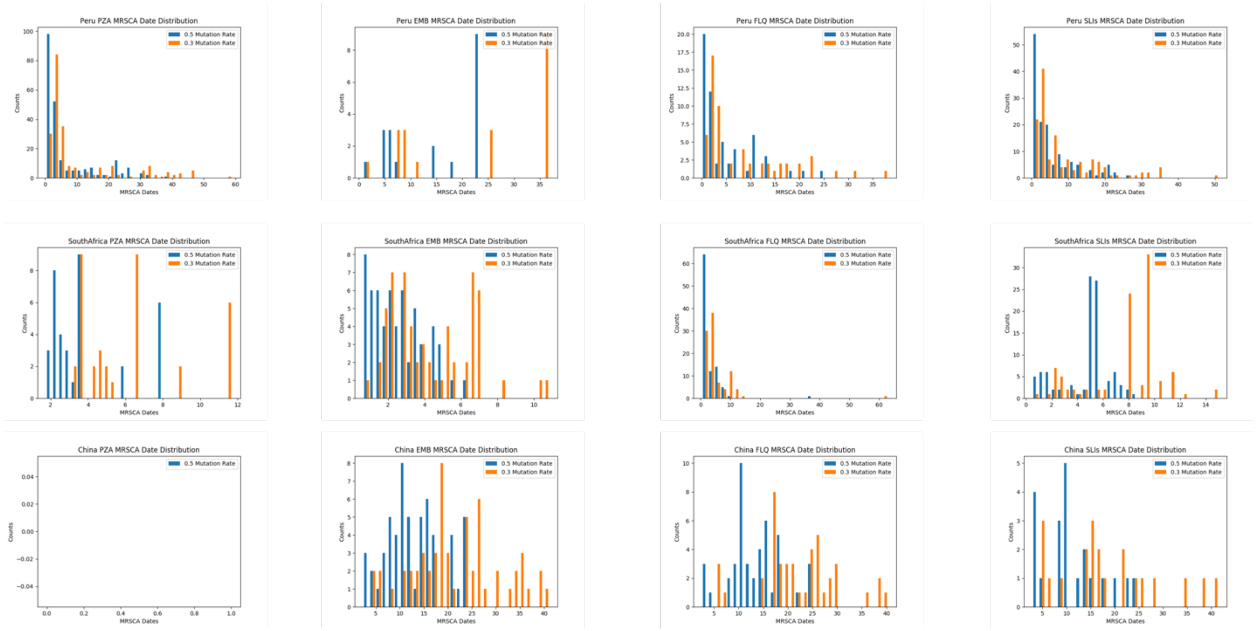

Supplementary Figure 4: Median RIF MRSCA date vs Gross Domestic Product (GDP) per capita for 12 countries for 0.3 and 0.5 Mutation Rate. Data plotted is provided in Supplementary Table 20.

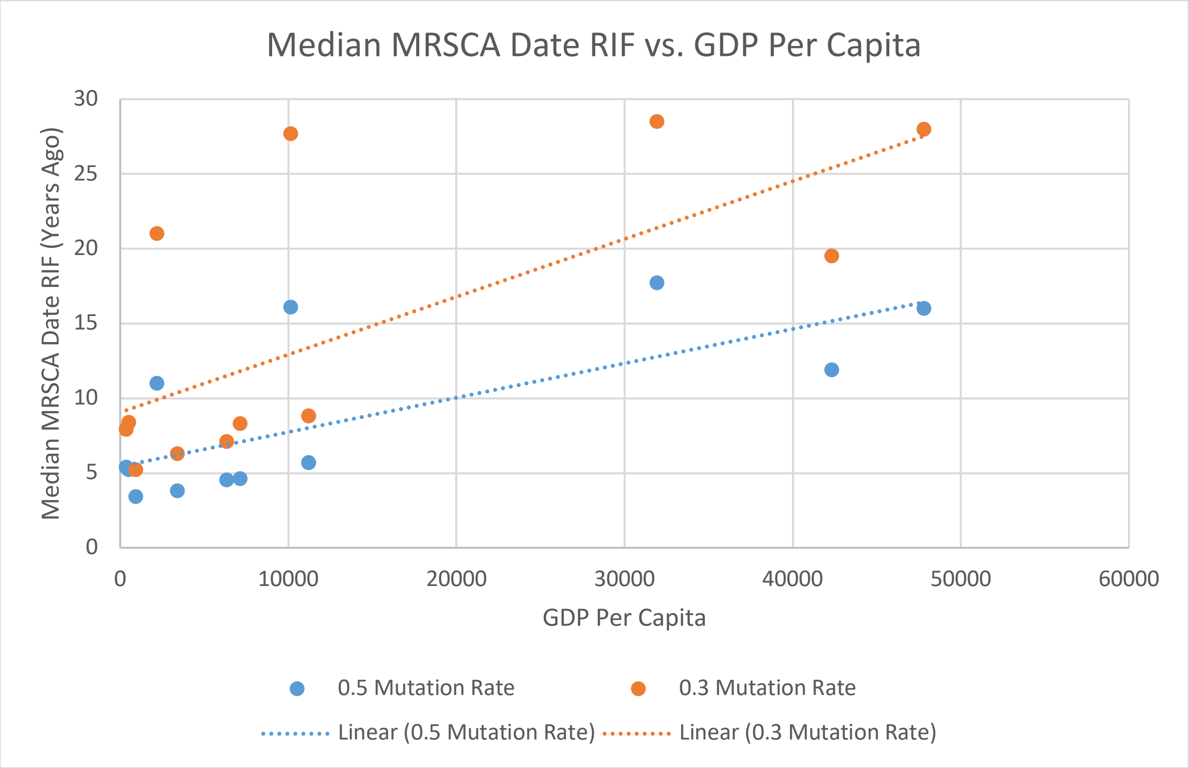

Supplementary References:

1. Ezewudo M, Borens A, Chiner-Oms Á, Miotto P, Chindelevitch L, Starks AM, et al. Integrating standardized whole genome sequence analysis with a global Mycobacterium tuberculosis antibiotic resistance knowledgebase. Scientific Reports. 2018 Oct 18;8(1):15382.

2. Schmieder R, Edwards R. Quality control and preprocessing of metagenomic datasets. Bioinformatics. 2011 Mar 15;27(6):863–4.

3. Wood DE, Salzberg SL. Kraken: ultrafast metagenomic sequence classification using exact alignments. Genome Biology. 2014 Mar 3;15(3):R46.

4. Li H. Aligning sequence reads, clone sequences and assembly contigs with BWA-MEM. arXiv:13033997 [q-bio] [Internet]. 2013 Mar 16 [cited 2019 Apr 11]; Available from: http://arxiv.org/abs/1303.3997

5. Picard toolkit [Internet]. Broad Institue, GitHub repository: Broad Institute; 2019. Available from: http://broadinstitute.github.io/picard/

6. Walker BJ, Abeel T, Shea T, Priest M, Abouelliel A, Sakthikumar S, et al. Pilon: An Integrated Tool for Comprehensive Microbial Variant Detection and Genome Assembly Improvement. PLOS ONE. 2014 Nov 19;9(11):e112963.

7. Coll F, McNerney R, Guerra-Assunção JA, Glynn JR, Perdigão J, Viveiros M, et al. A robust SNP barcode for typing *Mycobacterium tuberculosis* complex strains. Nature Communications. 2014 Sep 1;5:4812.

8. Seabold S, Perktold J. Statsmodels: Econometric and Statistical Modeling with Python. 2010;5.

9. WHO | Tuberculosis country profiles [Internet]. WHO. [cited 2019 May 9]. Available from: http://www.who.int/tb/country/data/profiles/en/

10. Stamatakis A. RAxML version 8: a tool for phylogenetic analysis and post-analysis of large phylogenies. Bioinformatics. 2014 May 1;30(9):1312–3.

11. Yang Z. Maximum likelihood phylogenetic estimation from DNA sequences with variable rates over sites: Approximate methods. J Mol Evol. 1994 Sep 1;39(3):306–14.

12. Suchard MA, Lemey P, Baele G, Ayres DL, Drummond AJ, Rambaut A. Bayesian phylogenetic and phylodynamic data integration using BEAST 1.10. Virus Evol [Internet]. 2018 Jan 1 [cited 2019 Apr 11];4(1). Available from: https://academic.oup.com/ve/article/4/1/vey016/5035211

13. Dixit A, Freschi L, Vargas R, Calderon R, Sacchettini J, Drobniewski F, et al. Whole genome sequencing identifies bacterial factors affecting transmission of multidrug-resistant tuberculosis in a high-prevalence setting. Scientific Reports. 2019 Apr 3;9(1):5602.

14. Sukumaran J, Holder MT. DendroPy: a Python library for phylogenetic computing. Bioinformatics. 2010 Jun 15;26(12):1569–71.

15. Oliphant T. NumPy: A guide to NumPy [Internet]. USA: Trelgol Publishing; 2006. Available from: http://www.numpy.org/

16. Jones E, Oliphant T, Peterson P. SciPy: Open Source Scientific Tools for Python. 2001 Jan 1;

17. Farhat MR, Sultana R, Iartchouk O, Bozeman S, Galagan J, Sisk P, et al. Genetic Determinants of Drug Resistance in Mycobacterium tuberculosis and Their Diagnostic Value. Am J Respir Crit Care Med. 2016 Feb 24;194(5):621–30.

18. Walker TM, Kohl TA, Omar SV, Hedge J, Del Ojo Elias C, Bradley P, et al. Whole-genome sequencing for prediction of Mycobacterium tuberculosis drug susceptibility and resistance: a retrospective cohort study. Lancet Infect Dis. 2015 Oct;15(10):1193–202.

19. Antibiotic resistance prediction for... | Wellcome Open Research [Internet]. [cited 2020 Aug 26]. Available from: https://wellcomeopenresearch.org/articles/4-191

20. World Economic Outlook, April 2019: Growth Slowdown, Precarious Recovery [Internet]. IMF. [cited 2019 Apr 11]. Available from: https://www.imf.org/en/Publications/WEO/Issues/2019/03/28/world-economic-outlook-april-2019

21. Kaufmann J, Schering AG. Analysis of Variance ANOVA. In: Wiley StatsRef: Statistics Reference Online [Internet]. American Cancer Society; 2014 [cited 2019 May 8]. Available from: https://onlinelibrary.wiley.com/doi/abs/10.1002/9781118445112.stat06938

22. Casali N, Nikolayevskyy V, Balabanova Y, Harris SR, Ignatyeva O, Kontsevaya I, et al. Evolution and transmission of drug-resistant tuberculosis in a Russian population. Nat Genet. 2014 Mar;46(3):279–86.

23. Rahman A, Sahrin M, Afrin S, Earley K, Ahmed S, Rahman SMM, et al. Comparison of Xpert MTB/RIF Assay and GenoType MTBDRplus DNA Probes for Detection of Mutations Associated with Rifampicin Resistance in Mycobacterium tuberculosis. PLOS ONE. 2016 Apr 7;11(4):e0152694.

24. policy_statement.pdf [Internet]. [cited 2019 May 25]. Available from: https://www.who.int/tb/features_archive/policy_statement.pdf

25. Chen H-Y, Yu M-C, Huang W-L, Wu M-H, Chang Y-L, Che C-R, et al. Molecular Detection of Rifabutin-Susceptible Mycobacterium tuberculosis. Journal of Clinical Microbiology. 2012 Jun 1;50(6):2085–8.

26. Tagliani E, Cabibbe AM, Miotto P, Borroni E, Toro JC, Mansjö M, et al. Diagnostic Performance of the New Version (v2.0) of GenoType MTBDRsl Assay for Detection of Resistance to Fluoroquinolones and Second-Line Injectable Drugs: a Multicenter Study. Journal of Clinical Microbiology. 2015 Sep 1;53(9):2961–9.

27. Xie YL, Chakravorty S, Armstrong DT, Hall SL, Via LE, Song T, et al. Evaluation of a Rapid Molecular Drug-Susceptibility Test for Tuberculosis. New England Journal of Medicine. 2017 Sep 14;377(11):1043–54.

28. Murray JF, Schraufnagel DE, Hopewell PC. Treatment of Tuberculosis. A Historical Perspective. Annals ATS. 2015 Dec 1;12(12):1749–59.

29. Zhang Y, Shi W, Zhang W, Mitchison D. Mechanisms of Pyrazinamide Action and Resistance. Microbiology Spectrum [Internet]. 2014 Jul 18 [cited 2019 Jun 29];2(4). Available from: http://www.asmscience.org/content/journal/microbiolspec/10.1128/microbiolspec.MGM2-0023-2013

30. Alapi EM, Fischer J. Table of Selected Analogue Classes. In: Analogue-based Drug Discovery [Internet]. John Wiley & Sons, Ltd; 2006 [cited 2019 Jun 29]. p. 441–552. Available from: https://onlinelibrary.wiley.com/doi/abs/10.1002/3527608001.ch23
